# Supplementary material for: Association of C-reactive protein with future development of diabetes: a population-based 7-year cohort study among Norwegian adults aged 30 and older in the Tromsø Study 2007–2016
Source: BMJ Open. 2023 Sep 29;13(9):e070284. doi: 10.1136/bmjopen-2022-070284 (PMC10546179; doi:10.1136/bmjopen-2022-070284)
Supplement: Author's manuscript [file bmjopen-2022-070284.draft_revisions.pdf]

BMJ Open is committed to open peer review. As part of this commitment we make the peer review history of every article we publish publicly available.

When an article is published we post the peer reviewers' comments and the authors' responses online. We also post the versions of the paper that were used during peer review. These are the versions that the peer review comments apply to.

The versions of the paper that follow are the versions that were submitted during the peer review process. They are not the versions of record or the final published versions. They should not be cited or distributed as the published version of this manuscript.

BMJ Open is an open access journal and the full, final, typeset and author-corrected version of record of the manuscript is available on our site with no access controls, subscription charges or pay-per-view fees (http://bmjopen.bmj.com).

If you have any questions on BMJ Open's open peer review process please email info.bmjopen@bmj.com

BMJ Open

The predictive value of C-reactive protein for future development of diabetes in a general population: The Tromsø Study 2007–2016

Journal:	*BMJ Open*
Manuscript ID	bmjopen-2022-070284
Article Type:	Original research
Date Submitted by the Author:	17-Nov-2022
Complete List of Authors:	Tong, Kit I.; University Health Network<br>Hopstock, Laila ; UiT The Arctic University of Norway<br>Cook, Sarah; Imperial College London; London School of Hygiene & Tropical Medicine
Keywords:	Epidemiology < TROPICAL MEDICINE, DIABETES & ENDOCRINOLOGY, General diabetes < DIABETES & ENDOCRINOLOGY

SCHOLARONE™
Manuscripts

I, the Submitting Author has the right to grant and does grant on behalf of all authors of the Work (as defined in the below author licence), an exclusive licence and/or a non-exclusive licence for contributions from authors who are: i) UK Crown employees; ii) where BMJ has agreed a CC-BY licence shall apply, and/or iii) in accordance with the terms applicable for US Federal Government officers or employees acting as part of their official duties; on a worldwide, perpetual, irrevocable, royalty-free basis to BMJ Publishing Group Ltd ("BMJ") its licensees and where the relevant Journal is co-owned by BMJ to the co-owners of the Journal, to publish the Work in this journal and any other BMJ products and to exploit all rights, as set out in our licence.

The Submitting Author accepts and understands that any supply made under these terms is made by BMJ to the Submitting Author unless you are acting as an employee on behalf of your employer or a postgraduate student of an affiliated institution which is paying any applicable article publishing charge ("APC") for Open Access articles. Where the Submitting Author wishes to make the Work available on an Open Access basis (and intends to pay the relevant APC), the terms of reuse of such Open Access shall be governed by a Creative Commons licence – details of these licences and which Creative Commons licence will apply to this Work are set out in our licence referred to above.

Other than as permitted in any relevant BMJ Author's Self Archiving Policies, I confirm this Work has not been accepted for publication elsewhere, is not being considered for publication elsewhere and does not duplicate material already published. I confirm all authors consent to publication of this Work and authorise the granting of this licence.

For peer review only

The predictive value of C-reactive protein for future development of diabetes in a general population: The Tromsø Study 2007–2016

Kit I. Tong,[1] Laila Arnesdatter Hopstock,[2] Sarah Cook,[3,4]

[1]Princess Margaret Cancer Centre, University Health Network, Toronto, Ontario, Canada. kit.tong@uhnresearch.ca

[2]Department of Health and Care Sciences, UiT The Arctic University of Norway, Tromsø, Norway. laila.hopstock@uit.no

[3]Faculty of Epidemiology and Population Health, London School of Hygiene & Tropical Medicine, London, UK. sarah.cook@lshtm.ac.uk

[4]School of Public Health, Imperial College London, London, UK.

Short running title: hs-CRP predicts incident diabetes

Correspondence to:

Dr Sarah Cook; sarah.cook@imperial.ac.uk and Dr Kit I. Tong; kit.tong@uhnresearch.ca

Word count: 3573

Number of tables: 2

Number of figures: 2

For peer review only

ABSTRACT

Introduction The extent to which the association of high-sensitivity C-reactive protein (hs-CRP) with incident diabetes is explained by obesity and hypertension remains unclear. This study aimed to investigate the predictive value of hs-CRP for diabetes development in a Norwegian general population.

Research Design and Methods In this cohort study 8,067 women and men aged 30 – 87 years participating in the sixth survey of the Tromsø Study 2007-2008 were followed for incident diabetes at the seventh survey 2015-2016. Diabetes defined by self-reported diabetes, medication use, and/or HbA1c ≥6.5% (≥ 48 mmol/mol) was modelled by logistic regression for the association with baseline hs-CRP, either stratified into 3-quantiles or as continuous variable, adjusted for demographic factors, behavioural and cardiovascular risk factors, lipid-lowering medication, and hypertension. The interaction by sex, body mass index (BMI), hypertension, or abdominal obesity were assessed by adding interaction terms in the fully adjusted model.

Results There were 320 (4.0%) diabetes cases after 7 years. After multivariate adjustment including covariates of obesity and hypertension, individuals in the highest hs-CRP tertile 3 had 73% higher odds of developing diabetes (OR = 1.73; p-value, 0.004; 95% CI, 1.20 – 2.49) when compared with the lowest tertile or 28% higher odds of incidence per one-log of hs-CRP increment (OR = 1.28; p-value, 0.003; 95% CI 1.09 – 1.50). There was no evidence for interaction between hs-CRP and sex, hypertension, BMI, or abdominal obesity.

Conclusions Raised hs-CRP is predictive for future diabetes in a Norwegian adult population. The CRP-diabetes association cannot be fully explained by obesity nor hypertension.

For peer review only - http://bmjopen.bmj.com/site/about/guidelines.xhtml

What is already known on this topic

⇒ High-sensitivity C-reactive protein (hs-CRP), a stable systemic inflammation biomarker, is associated with risk of diabetes.

⇒ Heterogeneous conclusions on whether the CRP-diabetes association can be explained by obesity and hypertension.

What this study adds

⇒ Elevated hs-CRP predicts future diabetes which cannot be fully explained by obesity nor hypertension.

How this study might affect research, practice, or policy

⇒ Elevated hs-CRP can be utilized as a predictive biomarker for future diabetes development to complement other current glycemic diagnostic parameters such as HbA1c.

⇒ Early prediction can inform preventive measures or innovation of anti-inflammatory therapeutics for disease intervention and management.

For peer review only

INTRODUCTION

In 2014 there were approximately 422 million adults with diabetes worldwide and the number of patients is constantly increasing.[1] Standardization of laboratory-based tests such as those that measure C-peptide, low-grade inflammation, β-cells mass, insulin resistance, and β-cells autoantibodies may facilitate better subtype diagnosis and subsequent precision care of diabetes.[1 2] Systemic inflammation has etiological implications to the pathogenesis of type 2 diabetes.[3] Inflammation is a cellular defense system against infections and is an essential mechanism for normal tissue homeostasis.[4] Inflammatory cells produce cytokines leading to the production of acute phase proteins in the liver to promote tissue repair.[5] When timely resolution reinstating cellular homeostasis fails, the resulting chronic inflammation can also lead to tissue damage.[6] Several risk factors for diabetes - aging, obesity, smoking and physical inactivity - have been found to be positively associated with low-grade inflammation.[7-10] Excessive energy intake and sedentary lifestyle contribute to fat accumulation in tissues including muscles, liver, and pancreas, which may trigger chronic low-grade inflammation through adipokine-induced production of pro-inflammatory cytokines and chemokines, ultimately leading to insulin resistance and β-cell dysfunction.[11] Indeed, the elevation of acute-phase proteins such as C-reactive protein (CRP), a marker for systemic inflammation, has previously been shown to be associated with development of type 1 and type 2 diabetes.[12]

There are conflicting conclusions in the literature on whether the association between high sensitivity (hs)-CRP and diabetes development is modified by sex[12-14] and whether the association is truly independent of hypertension,[15] general obesity,[16-18] and abdominal obesity.[13 19]

Lipid-lowering drugs such as statins, fibrates, nicotinic acid, and some other lipid modifying agents have both a lipid-lowering capacity and anti-inflammatory effect.[20] The use of these

For peer review only - http://bmjopen.bmj.com/site/about/guidelines.xhtml

medications has been found able to lower hs-CRP levels[21] while, conversely, the recipients of the drugs are inherently at risk of diabetes development due to indications for the medication use to reduce blood cholesterol and triglyceride levels.[22 23] Some, such as statins, are associated with elevated risk of new onset of diabetes while nicotinic acid is associated with impaired blood glucose control suggesting potential drug-induced side effects.[24] Some previous studies were not able to or did not adjust for the parameter of medication use[12 13 16] leading to the questions of potential residual confounding.

Population-based studies assessing the predictive value of CRP on future diabetes incidences has not been reported for the Scandinavian countries. We aimed to investigate the predictive values of hs-CRP on the development of diabetes after a 7-year follow-up among adults in Norway, a Nordic country with an increasing proportion of middle-aged and older adults and with a high prevalence of overweight, obesity, and hypertension.

For peer review only

RESEARCH DESIGN AND METHODS

Study Population

The Tromsø Study[25] is a population-based health study in the Tromsø municipality, Norway. Seven surveys (Tromsø1 – Tromsø7) have been conducted during 1974 – 2016, inviting whole birth cohorts and random sample of the Tromsø residents. Data collection include questionnaires, biological samples, and clinical examinations.

Study Sample

In Tromsø6 2007 – 2008, all residents aged 40 – 42 and 60 – 87 years (n=12,578), a 10% random sample of residents aged 30 – 39 years (n=1,056), a 40% random sample of residents aged 43 – 59 years (n=5,787) and all previous participants who attended Tromsø4 second visit (if not already included in the three groups above) (n=341) were invited.[26] In total, 12,984 individuals attended Tromsø6 (participation 66%). In Tromsø7 2015 – 2016, all residents aged 40 years and older were invited (N=32,591).[27] In total, 21,083 attended Tromsø7 (participation 65%).

In the current analysis, participants without prevalent diabetes in Tromsø6 who also attended Tromsø7 were included for this 7-year longitudinal analysis (figure 1). In total, 8,067 participants were included in the final analysis.

Diabetes was defined by self-reported diabetes diagnosis and/or the use of blood sugar lowering drugs (International WHO Anatomical Therapeutic Chemical (ATC)-code A10B) or insulin (ATC-code A10A),[28] and/or glycated haemoglobin (HbA1c) ≥ 6.5% (i.e., ≥ 48 mmol/mol).

Participants with baseline hs-CRP level ≥ 10mg/L were excluded to eliminate individuals with an acute inflammatory condition at the time of examination. Participants who had missing data for the baseline hs-CRP, HbA1c, self-reported diabetes diagnosis and use of insulin and/or blood sugar

For peer review only - http://bmjopen.bmj.com/site/about/guidelines.xhtml

lowering drugs; as well as participants with missing data for HbA1c, self-reported diabetes diagnosis and use of insulin and/or blood sugar lowering drugs at follow-up were excluded (figure 1).

Study Procedures

Data obtained by questionnaires, laboratory analyses, and clinical examinations have been described previously.[26 27 29] Briefly, blood samples without mandatory fasting were collected by EDTA anticoagulation tubes to measure HbA1c levels by high-performance liquid chromatography with Variant II (Bio-Rad Laboratories, Hercules, CA, USA) in Tromsø6 and by Tosoh G8 (Tosoh Bioscience, San Francisco, USA) in Tromsø7. Serum hs-CRP, total cholesterol, and high-density lipoprotein (HDL) cholesterol were measured with immunoturbidimetric and enzymatic colorimetric methods, respectively, by Modal PPE autoanalyzer (Roche Diagnostics Norway AS) in Tromsø6. Height in centimeters (cm) and weight in kilograms (kg) were measured by a Jenix DS 102 stadiometer (Dong Sahn Jenix, Seoul, Korea). Waist circumference was measured in cm by a Seca measuring tape at the level of the umbilicus. Three separate readings of systolic and diastolic blood pressure were taken, using a Dinamap Pro care 300 monitor (GE Healthcare, Norway), with 1-minute of intervals between each reading after a 2-minute seated rest. The mean systolic and diastolic blood pressure of readings 2 and 3 were used in this study.

Exposure (hs-CRP)

The baseline hs-CRP at Tromsø6, stratified into 3 quantiles of ascending values, was used as laboratory-based proxy measurement for chronic inflammation to deduce its impact on the odds

For peer review only - http://bmjopen.bmj.com/site/about/guidelines.xhtml

of developing diabetes over 7 years in Tromsø7. Additionally, in a sensitivity analysis, the log of hs-CRP was used as a continuous variable.

Outcome Definition (Incident Diabetes)

Known and unknown incident diabetes cases at Tromsø7 were identified by HbA1c ≥ 6.5% (i.e., ≥ 48 mmol/mol), and/or self-reported diabetes diagnosis ("Do you, or have you had diabetes?), and/or self-reported use of blood sugar lowering (ATC-code A10B) and/or insulin-containing medication (ATC-code A10A). Self-reported previous diabetes only was not defined as diabetes.

Confounders and Effect Modifiers

A priori confounders were demographic factors including age, sex, and education; behavioural risk factors including alcohol consumption frequency, daily smoking status, and physical activity; cardiovascular risk factors including body mass index (BMI), abdominal obesity, non-high-density lipoprotein (non-HDL) cholesterol; use of lipid-lowering medications (ATC-code C10); and hypertension. Age was divided into 5-year (40 – 74 years), 10-year (30 – 39 years) and 12-year (75 – 87 years) age groups, due to fewer participants in the lower and higher age ranges. Self-reported education, as a proxy for socioeconomic status, was defined by 5 levels (primary/secondary school, 1 – 2 years senior high school, high school diploma, < 4 years university, or ≥ 4 years university). Alcohol consumption frequency was defined by 5 levels (never drinker, monthly or less frequent drinker, 2 – 4 times per month, 2 – 3 times per week, or ≥ 4 times per week). Daily smoking was defined as current, previous or never. Physical activity was defined as sedentary, low, moderate, or vigorous. BMI was categorized as normal (< 25 kg/m^2), overweight (25 – 29.9 kg/m^2), or obese (≥ 30 kg/m^2). Abdominal obesity was categorized as abdominal obese

For peer review only - http://bmjopen.bmj.com/site/about/guidelines.xhtml

when waist-to-height ratio ≥ 0.5. Non-HDL cholesterol was derived by negating HDL cholesterol from total serum cholesterol. Hypertension was defined by systolic blood pressure ≥ 140 mmHg and/or diastolic blood pressure ≥ 90 mmHg, and/or self-reported use of blood pressure lowering drugs (ATC-code C02, antihypertensives; C03, diuretics; C07, beta blocking agents; C08, calcium channel blockers; C09, renin-angiotensin acting agents).[28]

Potential effect modifications were sex, BMI-categories, abdominal obesity, and hypertension defined above.

Statistical Analysis

The hs-CRP levels of the study participants (table 1) were stratified into three quantiles, 0.14 – 0.77 mg/L (tertile 1), 0.78 – 1.69 mg/L (tertile 2), and 1.70 – 9.98 mg/L (tertile 3). Age-standardized cumulative diabetes incidence rate across baseline hs-CRP tertiles was calculated using direct standardization to the 2013 European Standard Population.[30] Baseline characteristics of the sample population including demographic factors (age, sex, education), behavioural risk factors (frequency of alcohol consumption, daily smoking, and physical activity), cardiovascular risk factors (systolic/diastolic blood pressure, waist-to-height ratio/abdominal obesity, BMI, non-HDL cholesterol); medication use (blood pressure lowering and lipid-lowering drugs); and hypertension across hs-CRP tertiles were examined by Pearson's chi-squared test for categorical variables, by Kruskal-Wallis test for continuous variables with non-normal distribution, or by one-way ANOVA for continuous variables with normal distribution. Logistic regression models were built for the association of hs-CRP tertile with diabetes incidence and adjusted for a set of a *priori* determined covariates (sex, age, BMI, hypertension, abdominal obesity, non-HDL cholesterol, lipid-lowering drug use, alcohol consumption frequency, daily smoking status, physical activity,

For peer review only - http://bmjopen.bmj.com/site/about/guidelines.xhtml

and education). Interaction terms of sex (hs-CRP tertile x sex), anthropometric factors (hs-CRP tertile x BMI or hs-CRP tertile x abdominal obesity), or hypertension (hs-CRP tertile x hypertension) was added to the final multivariate regression model individually to test for interactions with hs-CRP on the association with incident diabetes using likelihood ratio tests.

Due to the skewed distribution, hs-CRP at mg/L was firstly log transformed to assess again its association as a continuous independent variable with diabetes incidence at Tromsø7. Logistic regression, with or without multivariate adjusted for *a priori* covariates described above, was used to evaluate the odds of diabetes cases by one-log increment of hs-CRP among all study sample.

All analysis was performed using STATA v.14.2 (StataCorp. 2015. Stata Statistical Software: Release 14. College Station, TX: StataCorp LP).

Patients or the public were not involved in the design, or conduct, or reporting, or dissemination plans of our research

For peer review only

For peer review only - http://bmjopen.bmj.com/site/about/guidelines.xhtml

RESULTS

In total 8,067 participants were included in the study. The number of participants excluded and the reasons for this are shown in figure 1. Five participants did not consent to medical research, 965 individuals had baseline diabetes, 418 individuals had baseline hs-CRP ≥10mg/L, 144 participants did not have baseline hs-CRP data, and 1 participant who did not have HbA1c nor self-reported diabetes diagnosis data with negative response to the use of insulin and blood sugar lowering drugs for determining baseline diabetes status were excluded. Among the 11,451 participants without baseline diabetes in Tromsø6 with hs-CRP <10mg/L and valid measurements, 8,071 also attended Tromsø7. Additionally, 4 individuals were excluded due to inadequate information in Tromsø7 to determine diabetes status. Hence, 8,067 (3,704 men, 45.9%; 4,363 women, 54.1%) participants without baseline diabetes nor acute inflammation in Tromsø6 aged 30 – 87 years were included in the final analysis (figure 1).

Baseline characteristics

The baseline characteristics (waist-to-height ratio, abdominal obesity, non-HDL cholesterol, systolic/diastolic blood pressure, hypertension, blood pressure lowering drugs, daily smoking, alcohol consumption frequency, BMI, physical activity, and education) of the participants exhibited statistical evidence for a difference across the 3 tertiles with increasing values of hs-CRP (p-values <0.001 – <0.0001) (table 1). Conversely, lipid-lowering drug usage only showed modest impact (p-value = 0.03). In tertiles 2 and 3, 55% and 60.5% of the participants were aged ≥ 55 years, respectively, while 82% and 90% of these participants were categorized as abdominal obesity. In addition, participants in tertiles 2 and 3 had higher percentage of overweight or obesity,

For peer review only - http://bmjopen.bmj.com/site/about/guidelines.xhtml

hypertension, physically inactive, daily smokers, lower education, and never or infrequent drinking when compared with those in tertile 1.

Age-standardized cumulative diabetes incidence

There was a more than 3-fold increase of overall age-standardized cumulative diabetes incidence in the highest hs-CRP tertile 3 (6.3%) when compared with the reference tertile 1 (2.0%). This trend was apparent in both women (1.5% vs 5.8%, 3.9-fold increase) and men (2.8% vs 7.2%, 2.6-fold increase) (figure 2).

Baseline hs-CRP predicts future diabetes development

There were 320 (4.0%) known and unknown incident cases of diabetes during follow-up (table 2). The odds of diabetes incidence were almost 2 times (crude OR=1.93; p-value <0.001; 95% CI 1.37 – 2.71) and 3.5 times (crude OR=3.49; p-value <0.001; 95% CI 2.55 – 4.79) higher among participants of hs-CRP tertile 2 and tertile 3, respectively. Although the strength of the association reduced in the fully adjusted model, positive association remained and the participants in hs-CRP tertile 3 had 73% higher odds of future diabetes (OR = 1.73; p-value, 0.004; 95% CI, 1.20 – 2.49). The addition of the individual interaction term of hs-CRP tertile x sex (Likelihood-ratio tests (LRT) p-value, 0.902), hs-CRP tertile x BMI (LRT p-value, 0.807), hs-CRP tertile x hypertension (LRT p-value, 0.773), or hs-CRP tertile x abdominal obesity (LRT p-value, 0.632) to the multivariate model did not support any compound effects of these covariates.

Consistent with the analyses using hs-CRP tertiles, a positive association between log transformed hs-CRP and diabetes development was observed (table 2). For every one-log hs-CRP increment, there was 75% increase in odds of diabetes cases (crude OR = 1.75; p-value, <0.001; 95% CI 1.53 – 1.99). Positive association maintained after multivariate adjustment with 28% higher odds of

For peer review only

For peer review only - http://bmjopen.bmj.com/site/about/guidelines.xhtml

diabetes development (OR = 1.28; p-value, 0.003; 95% CI 1.09 – 1.50) per one-log increase of hs-CRP.

For peer review only

For peer review only - http://bmjopen.bmj.com/site/about/guidelines.xhtml

CONCLUSIONS

We found a marked association between high hs-CRP and future diabetes incidence at 7-year follow-up in this study of adults from a general Norwegian population.

Comparison to other studies

A prospective study conducted in Spain demonstrated a positive association between elevated hs-CRP with future diabetes development after adjusting for age, sex, and obesity[16] but a study in UK found that the association was attenuated and no longer significant after adjusting for BMI and waist-to-hip ratio.[19] While some others showed that the association became insignificant in men but maintained in women after adjusting for BMI, suggesting evidence of interaction between hs-CRP and sex on the association.[14 31] However, the present study did not observe evidence for interaction between hs-CRP and sex, BMI, abdominal obesity, or hypertension. The association was also maintained after adjusting for multiple factors including BMI and abdominal obesity. Whereas some other studies[14 16] did not exclude individuals with elevated baseline CRP levels above 10 mg/L, which might have uplifted the exposure level due to the potential underlying issues of acute inflammatory condition other than the risk associated with diabetes development. This heightened exposure threshold likely attenuated the true association of the exposure and outcome. A Finnish cohort study[31] relied primarily on the diagnosed diabetes data through medical registries and self-reported surveys, whereas a German case-control study[14] identified baseline diabetes solely by self-reported information. There might be limitation of misclassifying baseline diabetes due to bias on self-reported diabetes. The lack of validated laboratory-based tests such as HbA1c levels or fasting-blood sugar content in these studies might fail to capture any unknown diabetes at baseline, which might have led to non-differential misclassification and could have diluted the strength of the association. Whereas, in the German study, the differential misclassification of the

For peer review only - http://bmjopen.bmj.com/site/about/guidelines.xhtml

baseline unknown diabetes among the controls might have biased the association leaning towards null due to the case-control study design,[14] which in part might be explaining the attenuation in the strength of association after adjusting for BMI. Lee and colleagues,[19] which on the other hand, had used a HbA1c level cutoff of >7.0% (i.e., >53 mmol/mol) for identifying unknown diabetes status at baseline and during follow-up. While this higher cutoff had been used for defining diabetes status, this deviation from the cutoff of 6.5% (i.e., 48 mmol/mol) recommended by the World Health Organization (WHO)[32] could introduce inconsistencies with the findings of other studies which chose to follow the WHO recommended standards.

There is increasing awareness that elevated hs-CRP levels and diabetes incidences are associated with adiposity.[33] The association of elevated hs-CRP with central adiposity could be independent of BMI.[34] Some European studies adjusted their models for BMI but not for central adiposity.[14 16 31] Waist-to-hip ratio have been indicated having various sex-specific thresholds[35] and was found not significantly associated with the changes of HbA1c levels.[36] Although Lee and colleagues had adjusted for both BMI and waist-to-hip ratio,[19] the inherent limitation of the waist-to-hip ratio might have led to residual confounding in some of these published analyses. Indeed, Lee and colleagues suggested that the differential results found in a meta-analysis of 16 published studies might be due to, in part, the adjustment for different indexes of central adiposity.[19] The waist-to-height ratio had been found to be sex-, age-, and ethnicity-independent;[35] and the use of this index in the current study might have addressed more adequately the confounding impact of the fat storage profile for both men and women.

Aging-associated chronic inflammation has been proposed as playing a part in the etiology of type 2 diabetes.[37] Although aging has long been identified as a risk factor and positively correlates with pathogenesis, it would be challenging to pinpoint a particular age cutoff to inform clinical practice.

For peer review only - http://bmjopen.bmj.com/site/about/guidelines.xhtml

Previous research have suggested multiple sources of aging-associated chronic inflammation including age-related accumulation of damaged cells, changes in gut microbiota over age, increasing cellular senescence and the subsequent secretion of proinflammatory cytokines namely the senescence-associated secretory phenotype or SASP, and age-associated modification of the immune system among others.[37] Although fat is a good source of inflammatory cytokines, obesity alone may not represent the entire spectrum of mechanisms underlying the development of diabetes via chronic systemic inflammation.

Prediabetes state, such as defined by HbA1c between 5.7 – 6.4% or 39 – 47 mmol/ml, has been considered as risk for diabetes transformation.[38] Although positive association had been previously implicated,[39 40] the temporal relationship between elevated hs-CRP and prediabetes remained unclear. Glycemic parameters such as HbA1c could provide estimates to reflect the average blood glucose concentration of an individual over the preceding 2 to 3 months. Nevertheless, the method also elicits certain extent of inaccuracies due to some glucose-independent variation, although corrective modeling has recently been proposed.[41]

Strengths of the study

This study has several strengths. The 7-year follow-up study allowed us to investigate the temporal association of hs-CRP on diabetes development. The study population was recruited and sampled from all residents of the Tromsø municipality with validated systematic data collection for each of the repetitive surveys, including the questionnaires, the clinical examinations and the lab assessments. The data being analyzed were of high quality with high participation in the study, and low levels of missing data in general (<11%) – which garners high internal validity. No findings from similar population-based studies conducted in the Scandinavian countries investigating the association between CRP and incident diabetes have been previously reported.

For peer review only - http://bmjopen.bmj.com/site/about/guidelines.xhtml

The current findings may inform the predictive value of hs-CRP on public health strategies for prevention and surveillance of diabetes in the Nordic region where lifestyle, culture, demography, health care and socioeconomic factors resemble Norway.

The data available in the current analysis included the information of the use of lipid-lowering drugs, which are used in primary and secondary prevention of cardiovascular diseases but have previously been shown capable in reducing hs-CRP levels but at the same time might contribute to drug-induced side effects of impaired glycemic control or new onset of diabetes.[24] These features might have led to residual confounding in some other previous studies which were not able to or did not include medication use in their multivariate-adjusted models.[12 13] Moreover, the current study had utilized a large overall cohort sample size.

Study limitations

Limitations of this study include the lack of information of diabetes subtype. Since the study sample only included adults 30 years of age and above, primarily newly developed type 2 diabetes were expected. The study focused on a Norwegian general population and hence the conclusion may not be generalizable beyond this region. Since the baseline and future developed diabetes cases were self-reported, there might be potential non-differential misclassification of the outcome due to reporting bias. Similarly, self-reported medication use did not come with physician, medical record, or medical registry confirmation which might have led to potential measurement error in the outcome. Nevertheless, previous studies have found that self-reported diabetes[42-44] often accounted for high specificity (84.5% to 99.7%) although can exhibit low sensitivity (41.5% to 80.4%). Previous studies had reported the impact from socioeconomic position and demographic characteristics such that the sensitivity of self-reported diabetes can be elevated with higher education levels and among urban dwellers.[44] In the current study, validated laboratory-based

For peer review only - http://bmjopen.bmj.com/site/about/guidelines.xhtml

measurement of the HbA1c level was utilized in addition to self-reported diabetes and diabetes medication use to enhance both the sensitivity and specificity when capturing the known and unknown true positive and false negative diabetes cases.

We did not include information on diet, which is another potential confounder. Diet can be negatively or positively associated with hs-CRP level and with future diabetes development depending on the diet types and patterns.[45] It would be of interest to include this parameter in future studies. In addition, diet is also linked with socioeconomic status which was apparently correlated with hs-CRP level as depicted in this report.

As chronic inflammation relates to the etiology of type 1 diabetes, it would be of interest to study its association with hs-CRP by utilizing a more defined type 1 diabetes cohort. It is also of interest to include hormone replacement therapy[46 47] as a potential confounder since this medication has been implicated as associated with elevated hs-CRP levels but also implicated in the reduction of abdominal fat.

In conclusion, this study demonstrated that hs-CRP is a predictor for future diabetes development in a population-based sample of Norwegian adults. In combination with glycemic parameters such as HbA1c or fasting plasma glucose, this stable blood-based biomarker can potentially be used to facilitate risk group stratification, which may provide opportunities for early prevention or intervention of diabetes through clinical practice, public health education, and anti-inflammatory therapeutics development. Previous studies[47 48] had depicted ethnic variations of CRP levels. When comparing with white, after multivariate adjustment including BMI, black individuals had higher serum CRP concentrations while Asians including Chinese and Japanese had lower levels. Future studies to determine the potentially ethnicity-dependent cut-offs of hs-CRP for risk stratification are warranted.

For peer review only

ACKNOWLEGMENTS

We would like to thank the participants of the Tromsø Study. We would like to extend our gratitude to Dr. Anne Elise Eggen, Dr. Ola Løvsletten, Ms. Julie-Helene D. Sørensen, and the members of the Data and Publication Committee of UiT The Arctic University of Norway to make the Tromsø dataset available for this study.

For peer review only

CONTRIBUTORS

K.I.T. conceived the research question of the present study based on the suggestion from S.C. on a project relating to diabetes and the Tromsø Study. K.I.T. planned out the study design, analyzed and interpreted the data, constructed figures and tables, and wrote the manuscript. L.A.H. and S.C. contributed to the discussion of the research question, study design, analysis, and data interpretation. L.A.H. and S.C. took part in the critical revision of the manuscript and provided overall supervision of the study. S.C. coordinated the collaboration of the study. L.A.H took part in the data collection of Tromsø6 and Tromsø7. L.A.H. took part in the planning and management of Tromsø7. K.I.T. is the guarantor of this work and, as such, had full access to all the data in the study and takes responsibility for the integrity of the data and the accuracy of the data analysis. All authors approved the final manuscript.

For peer review only

For peer review only - http://bmjopen.bmj.com/site/about/guidelines.xhtml

COMPLETING INTERESTS

Authors declare no potential conflicts of interest relevant to this article.

For peer review only

For peer review only - http://bmjopen.bmj.com/site/about/guidelines.xhtml

ETHICS APPROVAL

Tromsø6 was approved by the Data Inspectorate of Norway and the Regional Committee for Medical and Health Research Ethics Northern Norway (REC North) (Ref. 121/2006) and Tromsø7 by the Norwegian Data Protection Authority (Ref. 14/01463-4/CGN) and REC North (Ref. 2014/940). The current study was approved by the London School of Hygiene & Tropical Medicine Ethics Committee (Ref. 2566).

For peer review only

For peer review only - http://bmjopen.bmj.com/site/about/guidelines.xhtml

DATA AVAILABILITY STATEMENT

Data are not publicly available but may be obtained from UiT The Arctic University of Norway through application and project approval by the Data and Publication Committee and are not publicly available. The data used in this study are owned and managed by UiT The Arctic University of Norway (https://uit.no/research/tromsostudy).

For peer review only

FUNDING STATEMENT

Funding for the Tromsø Study was obtained from UiT The Arctic University of Norway, Northern Norway Regional Health Authority, Ministry of Health and Care Services, Norwegian Research Council, and various public and charity research funds in Norway. There was no external funding for this specific paper. The funding bodies have no role in the design of the study, data collection, analysis, interpretation of data, or in writing the manuscript.

For peer review only

For peer review only - http://bmjopen.bmj.com/site/about/guidelines.xhtml

REFERENCES

1 Classification of diabetes mellitus. Geneva: World Health Organization. 2019. [Article online], 2019. Available: https://www.who.int/publications/i/item/classification-of-diabetes-mellitus [Accessed 11 Sept 2021].
2 Schwartz SS, Epstein S, Corkey BE, *et al*. The Time Is Right for a New Classification System for Diabetes: Rationale and Implications of the β-Cell-Centric Classification Schema. *Diabetes Care* 2016;39:179–186.
3 Shoelson SE, Lee J, Goldfine AB. Inflammation and insulin resistance. *J Clin Invest* 2006;116:1793–1801.
4 Medzhitov R. Origin and physiological roles of inflammation. *Nature* 2008;454:428–435.
5 Castell JV, Gómez-Lechón MJ, David M, *et al*. Acute-phase response of human hepatocytes: regulation of acute-phase protein synthesis by interleukin-6. *Hepatology* 1990;12:1179–1186.
6 Chen L, Deng H, Cui H, *et al*. Inflammatory responses and inflammation-associated diseases in organs. *Oncotarget* 2018;9:7204–7218.
7 Guarner V, Rubio-Ruiz ME. Low-grade systemic inflammation connects aging, metabolic syndrome and cardiovascular disease. *Interdiscip Top Gerontol* 2015;40:99–106.
8 Visser M, Bouter LM, McQuillan GM, *et al*. Elevated C-reactive protein levels in overweight and obese adults. *JAMA* 1999;282:2131–2135.
9 Bazzano LA, He J, Muntner P, *et al*. Relationship between cigarette smoking and novel risk factors for cardiovascular disease in the United States. *Ann Intern Med* 2003;138:891–897.
10 Yu Z, Ye X, Wang J, *et al*. Associations of physical activity with inflammatory factors, adipocytokines, and metabolic syndrome in middle-aged and older chinese people. *Circulation* 2009;119:2969–2977.
11 Tsalamandris S, Antonopoulos AS, Oikonomou E, *et al*. The Role of Inflammation in Diabetes: Current Concepts and Future Perspectives. *Eur Cardiol* 2019;14:50–59.
12 Doi Y, Kiyohara Y, Kubo M, *et al*. Elevated C-reactive protein is a predictor of the development of diabetes in a general Japanese population: the Hisayama Study. *Diabetes Care* 2005;28:2497–2500.
13 Thorand B, Löwel H, Schneider A, *et al*. C-reactive protein as a predictor for incident diabetes mellitus among middle-aged men: results from the MONICA Augsburg cohort study, 1984-1998. *Arch Intern Med* 2003;163:93–99.
14 Thorand B, Baumert J, Kolb H, *et al*. Sex differences in the prediction of type 2 diabetes by inflammatory markers: results from the MONICA/KORA Augsburg case-cohort study, 1984-2002. *Diabetes Care* 2007;30:854–860.
15 Duncan BB, Schmidt MI, Pankow JS, *et al*. Low-grade systemic inflammation and the development of type 2 diabetes: the atherosclerosis risk in communities study. *Diabetes* 2003;52:1799–1805.
16 Rubio-Martín E, Soriguer F, Gutiérrez-Repiso C, *et al*. C-reactive protein and incidence of type 2 diabetes in the Pizarra study. *Eur J Clin Invest* 2013;43:159–167.
17 Pradhan AD, Manson JE, Rifai N, *et al*. C-reactive protein, interleukin 6, and risk of developing type 2 diabetes mellitus. *JAMA* 2001;286:327–334.
18 Effoe VS, Correa A, Chen H, *et al*. High-Sensitivity C-Reactive Protein Is Associated With Incident Type 2 Diabetes Among African Americans: The Jackson Heart Study. *Diabetes Care* 2015;38:1694–1700.

For peer review only

19 Lee CC, Adler AI, Sandhu MS, *et al*. Association of C-reactive protein with type 2 diabetes: prospective analysis and meta-analysis. *Diabetologia* 2009;52:1040–1047.

20 Hovland A, Retterstøl K, Mollnes TE, *et al*. Anti-inflammatory effects of non-statin low-density lipoprotein cholesterol-lowering drugs: an unused potential? *Scand Cardiovasc J* 2020;54:274–279.

21 Albert MA, Danielson E, Rifai N, *et al*. Effect of statin therapy on C-reactive protein levels: the pravastatin inflammation/CRP evaluation (PRINCE): a randomized trial and cohort study. *JAMA* 2001;286:64–70.

22 Oh RC, Lanier JB. Management of hypertriglyceridemia. *Am Fam Physician* 2007;75:1365–1371.

23 Beshara A, Cohen E, Goldberg E, *et al*. Triglyceride levels and risk of type 2 diabetes mellitus: a longitudinal large study. *J Investig Med* 2016;64:383–387.

24 Kosmas CE, Silverio D, Sourlas A, *et al*. Impact of lipid-lowering therapy on glycemic control and the risk for new-onset diabetes mellitus. *Drugs Context* 2018;7:212562.

25 Jacobsen BK, Eggen AE, Mathiesen EB, *et al*. Cohort profile: the Tromsø Study. *Int J Epidemiol* 2012;41:961–967.

26 Eggen AE, Mathiesen EB, Wilsgaard T, *et al*. The sixth survey of the Tromsø Study (Tromsø6) in 2007-08: collaborative research in the interface between clinical medicine and epidemiology: study objectives, design, data collection procedures, and attendance in a multipurpose population-based health survey. *Scand J Public Health* 2013;41:65–80.

27 The seventh survey of the Tromsø Study. [Article online]. Available: https://uit.no/research/tromsostudy/project?pid=708909 [Accessed 11 Sept 2021].

28 WHO collaborating centre for drug statistics methodology, Guidelines for ATC classification and DDD assignment, 2022. [Article online], 2021. Available: https://www.whocc.no/filearchive/publications/2022_guidelines_web.pdf [Accessed 10 Jan 2022].

29 Langholz PL, Wilsgaard T, Njølstad I, *et al*. Trends in known and undiagnosed diabetes, HbA1c levels, cardiometabolic risk factors and diabetes treatment target achievement in repeated cross-sectional surveys: the population-based Tromsø Study 1994-2016. *BMJ Open* 2021;11:e041846.

30 Pace M LG, Glickman M, Grande E, *et al*. Revision of the European Standard Population, Report of Eurostat's task force, 2013 edition. [Article online], 2013. Available: https://ec.europa.eu/eurostat/documents/3859598/5926869/KS-RA-13-028-EN.PDF/e713fa79-1add-44e8-b23d-5e8fa09b3f8f [Accessed 11 Sept 2021].

31 Hu G, Jousilahti P, Tuomilehto J, *et al*. Association of serum C-reactive protein level with sex-specific type 2 diabetes risk: a prospective finnish study. *J Clin Endocrinol Metab* 2009;94:2099–2105.

32 Use of glycated haemoglobin (HbA1c) in the diagnosis of diabetes mellitus. Abbreviated report of a WHO consultation. [Article online], 2011. Available: https://apps.who.int/iris/bitstream/handle/10665/70523/WHO_NMH_CHP_CPM_11.1_eng.pdf?sequence=1&isAllowed=y [Accessed 11 Sept 2021].

33 Lebovitz HE, Banerji MA. Visceral adiposity is causally related to insulin resistance. *Diabetes Care* 2005;28:2322–2325.

34 Lapice E, Maione S, Patti L, *et al*. Abdominal adiposity is associated with elevated C-reactive protein independent of BMI in healthy nonobese people. *Diabetes Care* 2009;32:1734–1736.

For peer review only

35 Yoo EG. Waist-to-height ratio as a screening tool for obesity and cardiometabolic risk. Korean J Pediatr 2016;59:425–431.
36 Hsieh SD, Yoshinaga H. Abdominal fat distribution and coronary heart disease risk factors in men-waist/height ratio as a simple and useful predictor. *Int J Obes Relat Metab Disord* 1995;19:585–589.
37 Franceschi C, Campisi J. Chronic inflammation (Inflammaging) and its potential contribution to age-associated diseases. *J Gerontol A Biol Sci Med Sci* 2014;69(Suppl 1):S4–9.
38 Tabák AG, Herder C, Rathmann W, *et al*. Prediabetes: A high-risk state for developing diabetes. *Lancet* 2012; 379:2279–2290.
39 Kato K, Otsuka T, Saiki Y, *et al*. Association between elevated C-reactive protein levels and prediabetes in adults, particularly impaired glucose tolerance. *Can J Diabetes* 2019;43:40–45.
40 Jaiswal A, Tabassum R, Podder A, *et al*. Elevated level of C-reactive protein is associated with risk of prediabetes in Indians. *Atherosclerosis* 2012;222:495–501.
41 Malka R, Nathan DM, Higgins JM. Mechanistic modeling of hemoglobin glycation and red blood cell kinetics enables personalized diabetes monitoring. *Sci Transl Med* 2016;8:359ra130.
42 Okura Y, Urban LH, Mahoney DW, *et al*. Agreement between self-report questionnaires and medical record data was substantial for diabetes, hypertension, myocardial infarction and stroke but not for heart failure. *J Clin Epidemiol* 2004;57:1096–1103.
43 Schneider ALC, Pankow JS, Heiss G, *et al*. Validity and reliability of self-reported diabetes in the atherosclerosis risk in communities study. *Am J Epidemiol* 2012;176:738–743.
44 Yuan X, Liu T, Wu L, *et al*. Validity of self-reported diabetes among middle-aged and older Chinese adults: the China health and retirement longitudinal study. *BMJ Open* 2015;5:e006633.
45 van Woudenbergh GJ, Kuijsten A, Tigcheler B, *et al*. Meat consumption and its association with C-reactive protein and incident type 2 diabetes: the Rotterdam Study. *Diabetes Care* 2012;35:1499–1505.
46 Pradhan AD, Manson JE, Rossouw JE, *et al*. Inflammatory biomarkers, hormone replacement therapy, and incident coronary heart disease: prospective analysis from the Women's Health Initiative observational study. *JAMA* 2002;288:980–987.
47 Albert MA, Glynn RJ, Burling J, *et al*. C-reactive protein levels among women of various ethnic groups living in the United States (from the Women's Health Study). *Am J Cardiol* 2004;93:1238–1242.
48 Kelley-Hedgepeth A, Lloyd-Jones DM, Colvin A, *et al*. Ethnic differences in C-reactive protein concentrations. Clin Chem 2008;54:1027–1037.

For peer review only

	Tertiles of hs-CRP (mg/L)				
	**Tertile 1 (0.14 – 0.77)**	**Tertile 2 (0.78 – 1.69)**	**Tertile 3 (1.70 – 9.98)**	**p-value***	**Total**
**N**	2,716	2,673	2,678		8,067
**Sex**, n (%)					
Male	1,139 (41.9)	1,336 (50.0)	1,229 (45.9)	<0.001	3,704 (45.9)
Female	1,577 (58.1)	1,337 (50.0)	1,449 (54.1)		4,363 (54.1)
**Age**, n (col, %)					
30 – 39 years	108 (4.0)	94 (3.5)	78 (2.9)	<0.001	280 (3.5)
40 – 44 years	728 (26.8)	485 (18.1)	435 (16.2)		1,648 (20.4)
45 – 49 years	332 (12.2)	315 (11.8)	267 (10.0)		914 (11.3)
50 – 54 years	325 (12.0)	285 (10.7)	274 (10.2)		884 (11.0)
55 – 59 years	268 (9.9)	309 (11.6)	301 (11.2)		878 (10.9)
60 – 64 years	471 (17.3)	551 (20.6)	598 (22.3)		1,620 (20.1)
65 – 69 years	283 (10.4)	349 (13.1)	367 (13.7)		999 (12.4)
70 – 74 years	138 (5.1)	184 (6.9)	210 (7.8)		532 (6.6)
75 – 87 years	63 (2.3)	101 (3.8)	148 (5.5)		312 (3.9)
**hs-CRP**, mg/L					
Median (25th, 75th percentiles)	0.50 (0.38, 0.62)	1.12 (0.93, 1.39)	2.83 (2.13, 4.27)	0.0001	1.12 (0.62, 2.13)
**BMI category**, n (col, %)					
Normal, < 25 kg/m^2	1,507 (55.5)	865 (32.4)	564 (21.1)	<0.001	2,936 (36.4)
Overweight, 25 – <30 kg/m^2	1,048 (38.6)	1,362 (51.0)	1,270 (47.4)		3,680 (45.6)
Obese, ≥ 30 kg/m^2	160 (5.9)	445 (16.7)	843 (31.5)		1,448 (18.0)
Missing	1	1	1		3
**Hypertension**, n (col, %)					
Yes	845 (31.2)	1,199 (45.0)	1,458 (54.5)	<0.001	3,502 (43.5)
Missing	5	6	4		15
**Blood pressure**, mean ± SD					

For peer review only

Systolic, mmHg	127.65 ± 20.35	134.22 ± 22.11	137.52 ± 21.54	<0.0001	133.10 ± 21.73
Diastolic, mmHg	75.49 ± 10.12	78.10 ± 10.39	79.20 ± 10.65	<0.0001	77.59 ± 10.50
**Blood pressure lowering drugs**, n (col, %)					
In use	355 (13.1)	528 (19.8)	717 (26.8)	<0.001	1,600 (19.8)
**Waist-to-height ratio**					
Mean ± SD	0.52 ± 0.06	0.55 ± 0.06	0.58 ± 0.07	<0.0001	0.55 ± 0.07
**Abdominal obesity**, n (col, %)					
Yes	1,646 (62.8)	2,125 (81.7)	2,341 (90.1)	<0.001	6,112 (78.2)
Missing	96	73	80		249
**Non-HDL cholesterol**, mmol/L					
Mean ± SD	3.85 ± 1.02	4.20 ± 1.06	4.32 ± 1.08	<0.0001	4.12 ± 1.07
**Lipid-lowering drugs**					
In use, n (col, %)	260 (9.6)	296 (11.1)	315 (11.8)	0.030	871 (10.8)
**Alcohol consumption frequency**, n (col, %)					
Never drinker	172 (6.4)	218 (8.2)	242 (9.1)	<0.001	632 (7.9)
Monthly or less frequent	617 (22.9)	701 (26.5)	799 (30.1)		2,117 (26.5)
2 – 4 times a month	1,146 (42.5)	1,088 (41.1)	1,054 (39.7)		3,288 (41.1)
2 – 3 times a week	596 (22.1)	501 (18.9)	424 (16.0)		1,521 (19.0)
≥ 4 times a week	167 (6.2)	141 (5.3)	133 (5.0)		441 (5.5)
Missing	18	24	26		68
**Daily smoking**, n (col, %)					
Current	387 (14.4)	469 (17.7)	576 (21.8)	<0.001	1,432 (17.9)
Previously	1,147 (42.6)	1,115 (42.1)	1,171 (44.3)		3,433 (43.0)
Never	1,156 (43.0)	1,066 (40.2)	899 (34.0)		3,121 (39.1)
Missing	26	23	32		81
**Physical activity**, n (col, %)					
Sedentary	352 (13.6)	383 (15.3)	536 (21.6)	<0.001	1,271 (16.8)
Low	1,580 (60.8)	1,532 (61.0)	1,542 (62.2)		4,654 (61.3)
Moderate	589 (22.7)	552 (22.0)	378 (15.2)		1,519 (20.0)

For peer review only - http://bmjopen.bmj.com/site/about/guidelines.xhtml

Vigorous	76 (2.9)	44 (1.8)	25 (1.0)		145 (1.9)
Missing	119	162	197		478
**Education**, n (col, %)					
Primary/Secondary	516 (19.2)	641 (24.2)	762 (28.8)	<0.001	1,919 (24.0)
1-2 years senior high school	618 (22.9)	722 (27.3)	746 (28.2)		2,086 (26.1)
High School diploma	220 (8.2)	206 (7.8)	212 (8.0)		638 (8.0)
College/University < 4 years	542 (20.1)	504 (19.0)	479 (18.1)		1,525 (19.1)
College/University ≥ 4 years	799 (29.7)	575 (21.7)	450 (17.0)		1,824 (22.8)
Missing	21	25	29		75

Table 1 – Baseline characteristics of the study participants overall and across hs-CRP tertiles in the Tromsø Study 2007 – 2008.

* p-value across hs-CRP tertiles was obtained by Pearson's chi-squared test for categorial variables, by Kruskal-Wallis test for continuous variables with non-normal distribution, or by one-way ANOVA for continuous variables with normal distribution.

For peer review only

	Tertiles of hs-CRP (mg/L)				
	**Tertile 1** (0.14 – 0.77)	**Tertile 2** (0.78 – 1.69)	**Tertile 3** (1.70 – 9.98)	**p-value**	**Total**
**N**	2,716	2,673	2,678		8,067
New diabetes incidence *, n (%)	52 (1.9)	97 (3.6)	171 (6.4)	<0.001†	320 (4.0)
**Odds ratio, OR (p-value, 95% CI)**					
Crude model	1.00 (ref)	1.93 (<0.001, 1.37 – 2.71)	3.49 (<0.001, 2.55 – 4.79)		
Multivariate adjusted ‡	1.00 (ref)	1.21 (0.326, 0.83 – 1.78)	1.73 (0.004, 1.20 – 2.49)		
**OR (p-value, 95% CI) by 1 log increment of hs-CRP**					
Crude model		1.75 (<0.001, 1.53 – 1.99)			
Multivariate adjusted ‡		1.28 (0.003, 1.09 – 1.50)			

Table 2 – Crude and multivariate-adjusted logistic regression models for the association of hs-CRP and diabetes incidences in the Tromsø Study 2007 – 2016.

* Known and unknown diabetes incidence in Tromsø7. † p-value across hs-CRP tertiles by Pearson's chi-squared test. ‡ Adjusted for sex, age, body mass index, abdominal obesity, non-HDL cholesterol, lipid lowering drug use, hypertension, alcohol consumption frequency, daily smoking status, physical activity, and education.

For peer review only - http://bmjopen.bmj.com/site/about/guidelines.xhtml

FIGURE LEGENDS

Figure 1 – Study sample inclusion flow chart of the Tromsø Study 2007 – 2016.

Figure 2 – Age-standardized cumulative diabetes incidence across hs-CRP tertiles during follow-up in the Tromsø Study 2015 – 2016.

For peer review only

For peer review only - http://bmjopen.bmj.com/site/about/guidelines.xhtml

Figure 1

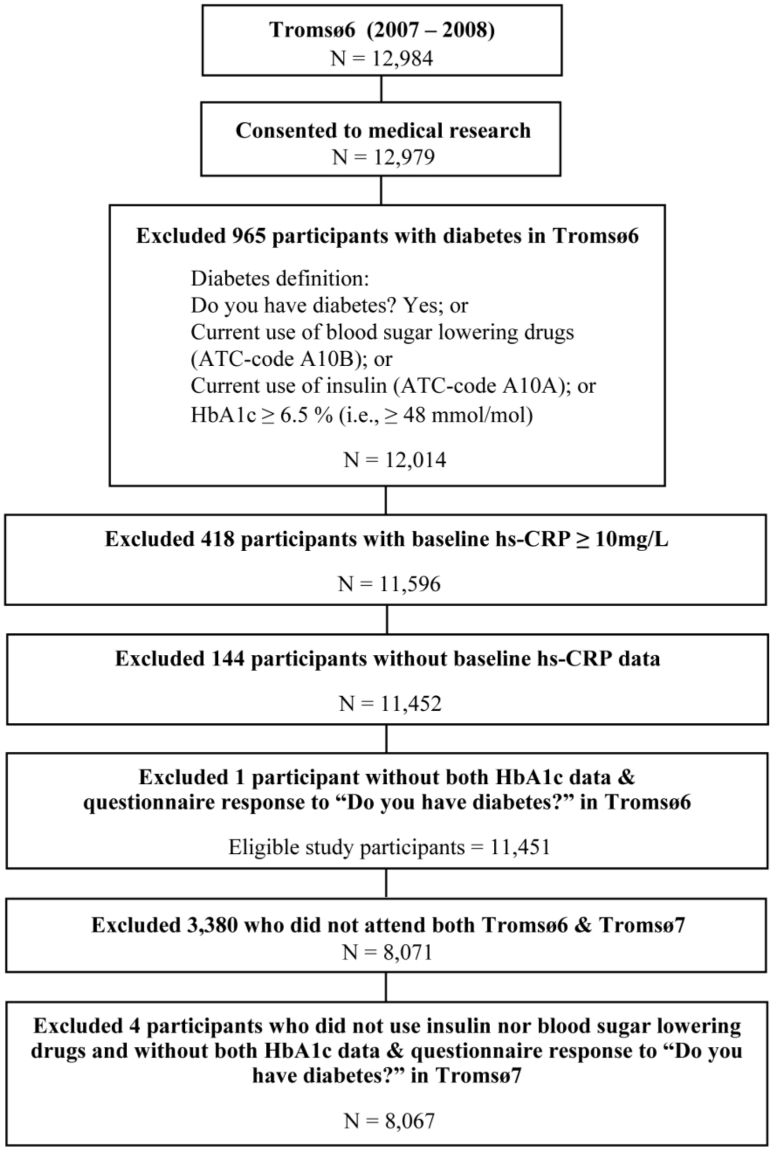

135x204mm (300 x 300 DPI)

Figure 2

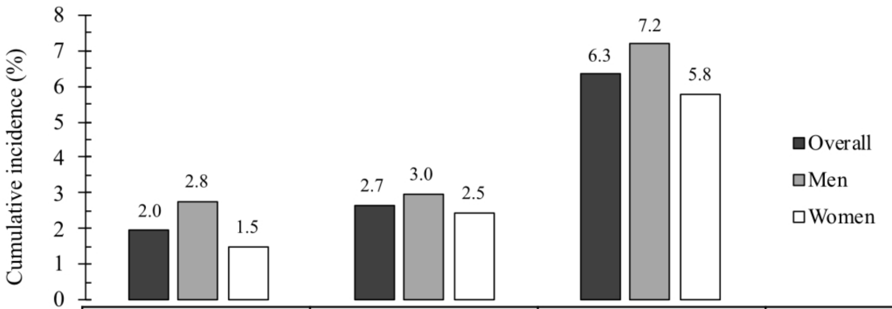

Tertile 1 (0.14 – 0.77)	Tertile 2 (0.78 – 1.69)	Tertile 3 (1.70 – 9.98)	hs-CRP (mg/L)
n=2,716	n=2,673	n=2,678	Overall (n=8,067)
n=1,139	n=1,336	n=1,229	Men (n=3,704)
n=1,577	n=1,337	n=1,449	Women (n=4,363)

187x101mm (300 x 300 DPI)

STROBE Statement—Checklist of items that should be included in reports of ***cohort studies***

	Item No	Recommendation	Page No
**Title and abstract**	1	(*a*) Indicate the study's design with a commonly used term in the title or the abstract	2
		(*b*) Provide in the abstract an informative and balanced summary of what was done and what was found	2
**Introduction**			
Background/rationale	2	Explain the scientific background and rationale for the investigation being reported	4-5
Objectives	3	State specific objectives, including any prespecified hypotheses	5
**Methods**			
Study design	4	Present key elements of study design early in the paper	6
Setting	5	Describe the setting, locations, and relevant dates, including periods of recruitment, exposure, follow-up, and data collection	6-7
Participants	6	(*a*) Give the eligibility criteria, and the sources and methods of selection of participants. Describe methods of follow-up	Figure 1
		(*b*) For matched studies, give matching criteria and number of exposed and unexposed	N/A
Variables	7	Clearly define all outcomes, exposures, predictors, potential confounders, and effect modifiers. Give diagnostic criteria, if applicable	7-9
Data sources/ measurement	8*	For each variable of interest, give sources of data and details of methods of assessment (measurement). Describe comparability of assessment methods if there is more than one group	7-9
Bias	9	Describe any efforts to address potential sources of bias	7-9
Study size	10	Explain how the study size was arrived at	6, Figure 1
Quantitative variables	11	Explain how quantitative variables were handled in the analyses. If applicable, describe which groupings were chosen and why	9
Statistical methods	12	(*a*) Describe all statistical methods, including those used to control for confounding	9-10
		(*b*) Describe any methods used to examine subgroups and interactions	10
		(*c*) Explain how missing data were addressed	10
		(*d*) If applicable, explain how loss to follow-up was addressed	6
		(*e*) Describe any sensitivity analyses	10
**Results**			
Participants	13*	(a) Report numbers of individuals at each stage of study—eg numbers potentially eligible, examined for eligibility, confirmed eligible, included in the study, completing follow-up, and analysed	Page 11/Figure 1
		(b) Give reasons for non-participation at each stage	Figure 1
		(c) Consider use of a flow diagram	Figure 1
Descriptive data	14*	(a) Give characteristics of study participants (eg demographic, clinical, social) and information on exposures and potential confounders	11
		(b) Indicate number of participants with missing data for each variable of interest	Table 1
		(c) Summarise follow-up time (eg, average and total amount)	Fixed follow up time –

For peer review only - http://bmjopen.bmj.com/site/about/guidelines.xhtml

			explained page 6
Outcome data	15*	Report numbers of outcome events or summary measures over time	Table 2 & Figure 2

For peer review only

For peer review only - http://bmjopen.bmj.com/site/about/guidelines.xhtml

Main results	16	(*a*) Give unadjusted estimates and, if applicable, confounder-adjusted estimates and their precision (eg, 95% confidence interval). Make clear which confounders were adjusted for and why they were included	Table 2
		(*b*) Report category boundaries when continuous variables were categorized	Table 2
		(*c*) If relevant, consider translating estimates of relative risk into absolute risk for a meaningful time period	n/a
Other analyses	17	Report other analyses done—eg analyses of subgroups and interactions, and sensitivity analyses	12
**Discussion**			
Key results	18	Summarise key results with reference to study objectives	13
Limitations	19	Discuss limitations of the study, taking into account sources of potential bias or imprecision. Discuss both direction and magnitude of any potential bias	16,17
Interpretation	20	Give a cautious overall interpretation of results considering objectives, limitations, multiplicity of analyses, results from similar studies, and other relevant evidence	17
Generalisability	21	Discuss the generalisability (external validity) of the study results	16,17
**Other information**			
Funding	22	Give the source of funding and the role of the funders for the present study and, if applicable, for the original study on which the present article is based	23

*Give information separately for exposed and unexposed groups.

Note: An Explanation and Elaboration article discusses each checklist item and gives methodological background and published examples of transparent reporting. The STROBE checklist is best used in conjunction with this article (freely available on the Web sites of PLoS Medicine at http://www.plosmedicine.org/, Annals of Internal Medicine at http://www.annals.org/, and Epidemiology at http://www.epidem.com/). Information on the STROBE Initiative is available at http://www.strobe-statement.org.

For peer review only

BMJ Open

The association of C-reactive protein with future development of diabetes: A population-based 7-year cohort study among Norwegian adults aged 30 and older in the Tromsø Study 2007 – 2016

Journal:	*BMJ Open*
Manuscript ID	bmjopen-2022-070284.R1
Article Type:	Original research
Date Submitted by the Author:	04-Apr-2023
Complete List of Authors:	Tong, Kit I.; University Health Network<br>Hopstock, Laila ; UiT The Arctic University of Norway<br>Cook, Sarah; Imperial College London; London School of Hygiene & Tropical Medicine
**Primary Subject Heading**:	Diabetes and endocrinology
Secondary Subject Heading:	Epidemiology
Keywords:	Epidemiology < TROPICAL MEDICINE, DIABETES & ENDOCRINOLOGY, General diabetes < DIABETES & ENDOCRINOLOGY

SCHOLARONE™
Manuscripts

I, the Submitting Author has the right to grant and does grant on behalf of all authors of the Work (as defined in the below author licence), an exclusive licence and/or a non-exclusive licence for contributions from authors who are: i) UK Crown employees; ii) where BMJ has agreed a CC-BY licence shall apply, and/or iii) in accordance with the terms applicable for US Federal Government officers or employees acting as part of their official duties; on a worldwide, perpetual, irrevocable, royalty-free basis to BMJ Publishing Group Ltd ("BMJ") its licensees and where the relevant Journal is co-owned by BMJ to the co-owners of the Journal, to publish the Work in this journal and any other BMJ products and to exploit all rights, as set out in our licence.

The Submitting Author accepts and understands that any supply made under these terms is made by BMJ to the Submitting Author unless you are acting as an employee on behalf of your employer or a postgraduate student of an affiliated institution which is paying any applicable article publishing charge ("APC") for Open Access articles. Where the Submitting Author wishes to make the Work available on an Open Access basis (and intends to pay the relevant APC), the terms of reuse of such Open Access shall be governed by a Creative Commons licence – details of these licences and which Creative Commons licence will apply to this Work are set out in our licence referred to above.

Other than as permitted in any relevant BMJ Author's Self Archiving Policies, I confirm this Work has not been accepted for publication elsewhere, is not being considered for publication elsewhere and does not duplicate material already published. I confirm all authors consent to publication of this Work and authorise the granting of this licence.

For peer review only

The association of C-reactive protein with future development of diabetes: A population-based 7-year cohort study among Norwegian adults aged 30 and older in the Tromsø Study 2007 – 2016

Kit I. Tong,[1] Laila Arnesdatter Hopstock,[2] Sarah Cook,[3,4]

[1]Princess Margaret Cancer Centre, University Health Network, Toronto, Ontario, Canada. kit.tong@uhnresearch.ca

[2]Department of Health and Care Sciences, UiT The Arctic University of Norway, Tromsø, Norway. laila.hopstock@uit.no

[3]Faculty of Epidemiology and Population Health, London School of Hygiene & Tropical Medicine, London, UK. sarah.cook@lshtm.ac.uk

[4]School of Public Health, Imperial College London, London, UK.

For peer review only

Short running title: Association of hs-CRP with incident diabetes

Correspondence to:

Dr Sarah Cook; sarah.cook@imperial.ac.uk and Dr Kit I. Tong; kit.tong@uhnresearch.ca

Word count: 4816

Number of tables: 2

Number of figures: 3

For peer review only - http://bmjopen.bmj.com/site/about/guidelines.xhtml

ABSTRACT

Objectives The extent to which observed associations between high-sensitivity C-reactive protein (hs-CRP) and incident diabetes are explained by obesity and hypertension remains unclear. This study aimed to investigate the association of hs-CRP with developing diabetes in a Norwegian general population sample.

Design A cohort study using two population-based surveys of the Tromsø Study: the sixth survey Tromsø 6 (2007-2008) as baseline and the seventh survey Tromsø7 (2015-2016) at follow-up.

Setting Tromsø municipality of Norway, a country with increasing proportion of older adults and a high prevalence of overweight, obesity, and hypertension.

Participants 8,067 women and men without diabetes, aged 30 – 87 years, at baseline Tromsø6 who subsequently also participated in Tromsø7.

Outcome measures Diabetes defined by self-reported diabetes, diabetes medication use, and/or HbA1c ≥6.5% (≥ 48 mmol/mol) was modelled by logistic regression for the association with baseline hs-CRP, either stratified into 3-quantiles or as continuous variable, adjusted for demographic factors, behavioural and cardiovascular risk factors, lipid-lowering medication use, and hypertension. Interactions by sex, body mass index (BMI), hypertension, or abdominal obesity were assessed by adding interaction terms in the fully adjusted model.

Results There were 320 (4.0%) diabetes cases after 7 years. After multivariable adjustment including obesity and hypertension, individuals in the highest hs-CRP tertile 3 had 73% higher odds of developing diabetes (OR = 1.73; p-value, 0.004; 95% CI, 1.20 – 2.49) when compared with the lowest tertile or 28% higher odds of incidence per one-log of hs-CRP increment (OR = 1.28; p-value, 0.003; 95% CI 1.09 – 1.50). There was no evidence for interaction between hs-CRP and sex, hypertension, BMI, or abdominal obesity.

For peer review only - http://bmjopen.bmj.com/site/about/guidelines.xhtml

Conclusions Raised hs-CRP was associated with future diabetes development in a Norwegian adult population sample. The CRP-diabetes association could not be fully explained by obesity or hypertension.

For peer review only

STRENGTHS AND LIMITATIONS OF THIS STUDY

⇒ The 7-year population-based cohort study design allowed us to investigate the temporal association between hs-CRP and future diabetes development.

⇒ The study utilized a large overall cohort sample size with high quality, high participation rate and a low level (<11%) of missing data, which garners high internal validity.

⇒ A number of potential confounders were taken into account including medication use of lipid-lowering drugs, which were not included in other previous studies.

⇒ Definition of the baseline and outcome future diabetes were based partly on self-reported diagnosis and self-reported diabetes medication use although the diabetes case definition was additionally based on the more robust HbA1c laboratory-based blood test to improve both sensitivity and specificity.

⇒ The study focused on a sample of the Norwegian general population from one municipality in Northern Norway and hence the conclusions may not be generalizable beyond this region.

For peer review only

INTRODUCTION

Diabetes is a diverse metabolic disorder primarily characterized by impaired glucose tolerance and hyperglycaemia resulting from insulin deficiency or resistance. In 2019 there were approximately 463 million people with diabetes worldwide and the number of patients is constantly increasing.[1] The growing hyperglycaemia-related deaths, health care burden, and the associated socio-economic outcomes are pressing issues. Identification of modifiable risk factors and predictive biomarkers for diabetes becomes important to offer opportunities for early prevention and intervention.

Due to the conflicting broad definitions of type 1 and type 2 diabetes, there was a recent proposal for a β-cell-centric classification system of diabetes that encompasses interactions between β-cells function and abundance with genetics, insulin resistance, environmental factors, immune dysregulation, and inflammation, which underlie a wide spectrum of hyperglycaemic phenotypes found in diabetic patients.[2] Standardization of laboratory-based tests such as those that measure C-peptide, low-grade inflammation, β-cells mass, insulin resistance, and β-cells autoantibodies may facilitate better subtype diagnosis and subsequent precision care of diabetes.[2 3]

While disease progression of type 1 diabetes involves inflammation of the islet cells leading to ultimate massive β-cells loss and clinical diabetes,[4 5] systemic inflammation is considered as one of the mechanisms mediating the pathogenesis of type 2 diabetes.[4] Inflammation is a cellular defense system against infections and is an essential mechanism for normal tissue homeostasis.[6] Inflammatory cells produce cytokines leading to the production of acute phase proteins in the liver to promote tissue repair.[7] However, when timely resolution reinstating cellular homeostasis fails, the resulting chronic inflammation can also lead to tissue damage.[8] Given the emerging role of inflammation in the pathophysiology of diabetes and the associated metabolic disorders, there is

For peer review only - http://bmjopen.bmj.com/site/about/guidelines.xhtml

increasing interest in the development of anti-inflammatory therapeutics to improve both the prevention and the management of diabetes with numerous clinical trials showing various promising effects on type 1 and type 2 diabetes patients.[4 9 10] Several risk factors for diabetes - aging, obesity, smoking and physical inactivity - have been found to be positively associated with low-grade inflammation.[11-14] Excessive energy intake and sedentary lifestyle contribute to fat accumulation in tissues including muscles, liver, and pancreas, which may trigger chronic low-grade inflammation through adipokine-induced production of pro-inflammatory cytokines and chemokines, ultimately leading to insulin resistance and β-cell dysfunction.[10] Indeed, the elevation of acute-phase proteins such as C-reactive protein (CRP), a marker for systemic inflammation, has previously been shown to be associated with development of type 1 and type 2 diabetes.[15 16]

Demographic factors such as sex,[17 18] age,[11 19] and education;[20] lifestyle or behaviour risk factors for diabetes and/or CRP levels such as alcohol consumption,[21 22] daily smoking,[13 23] and physical inactivity;[14 24] clinical risk factors such as high body mass index (BMI),[12 25] abdominal obesity,[12 26 27] non-HDL cholesterol,[28 29] medication use of lipid lowering drug,[30 31] and the comorbidity hypertension[32] are potential confounders that have been indicated associated with CRP levels and/or with diabetes incidences; but not mediating the pathway between the association of CRP and diabetes incidences (figure 1).

There are conflicting conclusions in the literature on whether the association between high sensitivity (hs)-CRP and diabetes development is modified by sex[15 33 34] and whether the association is truly independent of hypertension,[35] general obesity,[36-38] and abdominal obesity.[33 39] Evidences of interaction were also evident in some studies between CRP and sex,[34 40] BMI,[15 38] hypertension,[15 41] or with waist circumference,[38] while other studies however did not observe evidence of interaction between hs-CRP with sex nor with BMI.[42 43]

For peer review only - http://bmjopen.bmj.com/site/about/guidelines.xhtml

Lipid-lowering drugs such as statins, fibrates, nicotinic acid, and some other lipid modifying agents have both a lipid-lowering capacity and anti-inflammatory effect.[44] The use of these medications has been found able to lower hs-CRP levels[30] while, conversely, the recipients of the drugs are inherently at risk of diabetes development due to indications for the medication use to reduce blood cholesterol and triglyceride levels.[45 46] Some, such as statins, are associated with elevated risk of new onset of diabetes while nicotinic acid is associated with impaired blood glucose control suggesting potential drug-induced side effects.[31] Some previous studies were not able to or did not adjust for the parameter of medication use[15 33 36] leading to the questions of potential residual confounding.

According to Norwegian Institute of Public Health, there was high prevalence of overweight and obesity combined in Norway.[47] Using a recent revised consensus definition of metabolism syndrome from the harmonized Adult Treatment Panel-III (ATP-III) criteria,[48] a recent repeated large cohort cross-sectional study of the rural Northern Norway has found that the prevalence of age-standardized metabolic syndrome (MetS) has progressed from 31.2% in 2003-2004 (n=6550) to 35.6% in 2012-2014 (n=6004) with concomitant increase in the mean MetS severity, which was believed to be driven by the marked increase in abdominal obesity among the study population over the years.[49]

Population-based studies assessing CRP as a marker for future diabetes incidences has not been reported for the Scandinavian countries. We aimed to investigate the association of hs-CRP on the development of diabetes after an interval of 7-years among adults in Norway, a Nordic country with an increasing proportion of middle-aged and older adults and with a high prevalence of overweight, obesity, and hypertension.

For peer review only - http://bmjopen.bmj.com/site/about/guidelines.xhtml

METHODS

Setting

The Tromsø Study[50] is a population-based health study in the Tromsø municipality, Norway. Participants were invited from the whole birth cohorts and random sample of the Tromsø residents. Data collection include questionnaires, biological samples, and clinical examinations.

This study involved data from 2 surveys of the Tromsø Study: the sixth survey of the Tromsø Study (Tromsø6) 2007 – 2008 as baseline and the seventh survey of the Tromsø Study (Tromsø7) 2015 – 2016 as the follow-up time point after a 7-year interval.

In Tromsø6 2007 – 2008, all residents aged 40 – 42 and 60 – 87 years (n=12,578), a 10% random sample of residents aged 30 – 39 years (n=1,056), a 40% random sample of residents aged 43 – 59 years (n=5,787) and all previous participants who attended Tromsø4 second visit (if not already included in the three groups above) (n=341) were invited.[51] In total, 12,984 women and men attended Tromsø6 with 66% participation (figure 2)

In Tromsø7 2015 – 2016, all residents aged 40 years and older were invited (N=32,591).[52] In total, 21,083 women and men attended Tromsø7 with 65% participation (figure 2).

Study Design

A cohort study used two population-based surveys of the Tromsø Study: the sixth survey Tromsø6 (2007-2008) as baseline and the seventh survey Tromsø7 (2015-2016) at follow-up after 7-year interval. The association of the baseline hs-CRP, expressed as 3-quantiles, at Tromsø6 with future diabetes cases at Tromsø7 for the participants who did not have baseline diabetes at Tromsø6 was examined by logistic regression adjusted for a number of *a priori* potential confounding factors and then further tested for potential effect modifications by likelihood-ratio tests. The CRP-

For peer review only

For peer review only - http://bmjopen.bmj.com/site/about/guidelines.xhtml

diabetes association was further tested in the sensitivity analysis by repeating multivariable logistic regression using the baseline log hs-CRP, at Tromsø6, expressed as continuous independent variable.

Study Sample

Figure 2 depicts the flow chart for the selection of the study sample. In brief, participants without prevalent diabetes in Tromsø6 who had also attended Tromsø7 were included in this analysis.

In addition, individuals with baseline hs-CRP ≥10 mg/L, an indication of acute inflammatory condition at the time of examination, would be excluded in the final analysis to mitigate the risk of measurement error. As acute inflammation may resolve over a short period of time and may not necessarily transit into chronic inflammatory condition. A previous study[53] involving 5,111 men aged 48-77 in the Oslo Study showed that individuals with health conditions of osteoporosis, asthma, chronic bronchitis/emphysema, diabetes, or myocardial infarction had a mean CRP level of 6.53 mg/L, 5.01 mg/L, 4.42 mg/L, 4.53 mg/L, and 4.27 mg/L, respectively. These values were much less than hs-CRP of 10 mg/L.

In details, 12,984 participants attended Tromsø6. We excluded 5 participants who did not consent to medical research; 965 participants with baseline diabetes; 418 participants with baseline hs-CRP ≥10 mg/L; 144 participants without baseline hs-CRP data; and 1 participant without HbA1c nor self-reported diabetes diagnosis data with negative response to the use of insulin and blood sugar lowering drugs for determining baseline diabetes status. Among the 11,451 participants without baseline diabetes in Tromsø6 with hs-CRP <10 mg/L and valid measurements, a total of 8,071 also attended Tromsø7. Additionally, 4 participants were excluded due to inadequate information in Tromsø7 to determine diabetes status. Hence, 8,067 (3,704 men, 45.9%; 4,363

For peer review only - http://bmjopen.bmj.com/site/about/guidelines.xhtml

women, 54.1%) participants without baseline diabetes nor acute inflammation in Tromsø6 aged 30 – 87 years were included in the final analysis (figure 2).

Criteria defining diabetes condition is as described below in section "*Outcome Definition (Diabetes)*".

Data Collection Procedures

Data obtained by questionnaires, laboratory analyses, and clinical examinations have been described previously.[51 52 54] Briefly, blood samples without mandatory fasting were collected by EDTA anticoagulation tubes to measure HbA1c levels by high-performance liquid chromatography with Variant II (Bio-Rad Laboratories, Hercules, CA, USA) in Tromsø6 and by Tosoh G8 (Tosoh Bioscience, San Francisco, USA) in Tromsø7. Serum hs-CRP, total cholesterol, and high-density lipoprotein (HDL) cholesterol were measured with immunoturbidimetric and enzymatic colorimetric methods, respectively, by Modal PPE autoanalyzer (Roche Diagnostics Norway AS) in Tromsø6. Height in centimeters (cm) and weight in kilograms (kg) were measured by a Jenix DS 102 stadiometer (Dong Sahn Jenix, Seoul, Korea). Waist circumference was measured in cm by a Seca measuring tape at the level of the umbilicus. Three separate readings of systolic and diastolic blood pressure were taken, using a Dinamap Pro care 300 monitor (GE Healthcare, Norway), with 1-minute of intervals between each reading after a 2-minute seated rest. The mean systolic and diastolic blood pressure of readings 2 and 3 were used in this study.

Variables in the analyses

Exposure (hs-CRP) - The baseline hs-CRP at Tromsø6, stratified into 3 quantiles of ascending values, was used as laboratory-based proxy measurement for chronic inflammation to deduce its

For peer review only - http://bmjopen.bmj.com/site/about/guidelines.xhtml

impact on the odds of developing diabetes over 7 years in Tromsø7. Additionally, in the sensitivity analysis, hs-CRP was firstly log transformed due to its non-normally and skewed distribution. The log transformed hs-CRP was then used as a continuous independent variable to re-analyze the regression model.

Outcome Definition (Diabetes) - Known and unknown incident diabetes cases at Tromsø7 were identified by glycated hemoglobin (HbA1c) ≥ 6.5% (i.e., ≥ 48 mmol/mol), and/or self-reported diabetes diagnosis ("Do you, or have you had diabetes?), and/or self-reported use of blood sugar lowering (ATC-code A10B)[55] and/or insulin-containing medication (ATC-code A10A)[55] for treating symptoms. Self-reported previous diabetes only was not defined as diabetes.

Potential confounders - As described in the conceptual framework in figure 1 and in the introduction, previous studies[11-14 17-32] suggested that the following variables are potential confounders that have been indicated associated with CRP levels and/or with diabetes incidences, but not mediating the pathway between the association of CRP and diabetes incidence: They include demographic factors (age, sex, and education); behavioural risk factors (alcohol consumption frequency, daily smoking status, and physical activity); cardiovascular risk factors (body mass index (BMI), abdominal obesity, and non-high-density lipoprotein (non-HDL) cholesterol); use of lipid-lowering medications (ATC-code C10)[55]; and comorbid hypertension.

Potential effect modifiers - Previous studies[15 34 38 40 41] suggested that sex, BMI category, abdominal obesity, or hypertension may interact with hs-CRP although conflicting results have been found from other studies.[42 43]

Definition of the variables (confounders and/or effect modifiers):

For peer review only - http://bmjopen.bmj.com/site/about/guidelines.xhtml

- Age was divided into 5-year groups for the individuals aged 40 – 74 years. Due to data sparsity, the youngest and the eldest individuals were categorized into 10-year (30 – 39 years) and 12-year (75 – 87 years) age groups.
- Self-reported education, as a proxy for socioeconomic status, was defined by 5 levels (primary/secondary school, 1 – 2 years senior high school, high school diploma, < 4 years university, or ≥ 4 years university).
- Alcohol consumption frequency was defined by 5 levels (never drinker, monthly or less frequent drinker, 2 – 4 times per month, 2 – 3 times per week, or ≥ 4 times per week).
- Daily smoking was defined as current, previous or never.
- Physical activity was defined from self-report as sedentary (reading, watching TV, or other sedentary activity), low (walking, cycling, or other forms of exercise at least 4 hours a week), moderate (recreational sports, heavy gardening, etc. at least 4 hours a week), or vigorous (hard training or sports competitions, regularly several times a week).[51]
- BMI was categorized as normal (< 25 kg/m^2), overweight (25 – 29.9 kg/m^2), or obesity (≥ 30 kg/m^2).[47] Due to data sparsity, the participants who were underweight (< 18.5 kg/m^2) were combined into the normal BMI group.
- Abdominal obesity was categorized as abdominal obese when waist-to-height ratio ≥ 0.5. It was suggested by previous systematic review and meta-analysis studies that a waist-to-height ratio cutoff of 0.5 is suitable for adults of both sex and different ethnic backgrounds.[27 56]
- Non-HDL cholesterol was derived by negating HDL cholesterol from total serum cholesterol.
- Hypertension was defined by systolic blood pressure ≥ 140 mmHg and/or diastolic blood pressure ≥ 90 mmHg,[57] and/or self-reported use of blood pressure lowering drugs (ATC-code

For peer review only - http://bmjopen.bmj.com/site/about/guidelines.xhtml

C02, antihypertensives; C03, diuretics; C07, beta blocking agents; C08, calcium channel blockers; C09, renin-angiotensin acting agents)[55].

Statistical analysis

The hs-CRP levels of the study participants (table 1) were first stratified into three quantiles, 0.14 – 0.77 mg/L (tertile 1), 0.78 – 1.69 mg/L (tertile 2), and 1.70 – 9.98 mg/L (tertile 3) to conduct most of the analyses described below unless otherwise specified.

Age-standardized cumulative diabetes incidence - In figure 3, age-standardized cumulative diabetes incidence across baseline hs-CRP tertiles, overall or sex-specific, was calculated using direct standardization to the 2013 European Standard Population[58] to examine any dosage effect of hs-CRP exposure on outcome diabetes cases.

Baseline characteristics - In table 1, the descriptive statistics of the baseline characteristics for the sample population across the 3-quantiles of baseline hs-CRP was examined:

By Pearson's chi-squared test for categorical variables (sex, age, BMI category, hypertension, blood pressure lowering drugs, abdominal obesity, lipid-lowering drugs, alcohol consumption frequency, daily smoking, physical activity, education).

By Kruskal-Wallis test for continuous variables with non-normal distribution (hs-CRP).

By one-way ANOVA for continuous variables with normal distribution (waist-to-height ratio, non-HDL cholesterol, systolic blood pressure, diastolic blood pressure).

Logistic regression models - In table 2, crude model was built to examine the association of baseline hs-CRP tertiles with diabetes incidence in Tromsø7. The model was then multivariable adjusted for a set of a *priori* determined covariates (sex, age, BMI, hypertension, abdominal obesity, non-HDL cholesterol, lipid-lowering drug use, alcohol consumption frequency, daily

For peer review only - http://bmjopen.bmj.com/site/about/guidelines.xhtml

	Tertiles of hs-CRP (mg/L)				
	Tertile 1 (0.14 – 0.77)	Tertile 2 (0.78 – 1.69)	Tertile 3 (1.70 – 9.98)	p-value*	Total
**N**	2,716	2,673	2,678		8,067
**Sex**, n (%)					
Male	1,139 (41.9)	1,336 (50.0)	1,229 (45.9)	<0.001	3,704 (45.9)
Female	1,577 (58.1)	1,337 (50.0)	1,449 (54.1)		4,363 (54.1)
**Age**, n (col, %)					
30 – 39 years	108 (4.0)	94 (3.5)	78 (2.9)	<0.001	280 (3.5)
40 – 44 years	728 (26.8)	485 (18.1)	435 (16.2)		1,648 (20.4)
45 – 49 years	332 (12.2)	315 (11.8)	267 (10.0)		914 (11.3)
50 – 54 years	325 (12.0)	285 (10.7)	274 (10.2)		884 (11.0)
55 – 59 years	268 (9.9)	309 (11.6)	301 (11.2)		878 (10.9)
60 – 64 years	471 (17.3)	551 (20.6)	598 (22.3)		1,620 (20.1)
65 – 69 years	283 (10.4)	349 (13.1)	367 (13.7)		999 (12.4)
70 – 74 years	138 (5.1)	184 (6.9)	210 (7.8)		532 (6.6)
75 – 87 years	63 (2.3)	101 (3.8)	148 (5.5)		312 (3.9)
**hs-CRP**, mg/L					
Median (25th, 75th percentiles)	0.50 (0.38, 0.62)	1.12 (0.93, 1.39)	2.83 (2.13, 4.27)	0.0001	1.12 (0.62, 2.13)
**BMI category**, n (col, %)					
Normal, < 25 kg/m^2	1,507 (55.5)	865 (32.4)	564 (21.1)	<0.001	2,936 (36.4)
Overweight, 25 – 29.9 kg/m^2	1,048 (38.6)	1,362 (51.0)	1,270 (47.4)		3,680 (45.6)
Obesity, ≥ 30 kg/m^2	160 (5.9)	445 (16.7)	843 (31.5)		1,448 (18.0)
Missing	1	1	1		3
**Hypertension**, n (col, %)					
Yes	845 (31.2)	1,199 (45.0)	1,458 (54.5)	<0.001	3,502 (43.5)
Missing	5	6	4		15
**Blood pressure**, mean ± SD					
Systolic, mmHg	127.65 ± 20.35	134.22 ± 22.11	137.52 ± 21.54	<0.0001	133.10 ± 21.73
Diastolic, mmHg	75.49 ± 10.12	78.10 ± 10.39	79.20 ± 10.65	<0.0001	77.59 ± 10.50
**Blood pressure lowering drugs**, n (col, %)					
In use	355 (13.1)	528 (19.8)	717 (26.8)	<0.001	1,600 (19.8)
**Waist-to-height ratio**					
Mean ± SD	0.52 ± 0.06	0.55 ± 0.06	0.58 ± 0.07	<0.0001	0.55 ± 0.07
**Abdominal obesity**, n (col, %)					
Yes	1,646 (62.8)	2,125 (81.7)	2,341 (90.1)	<0.001	6,112 (78.2)
Missing	96	73	80		249
**Non-HDL cholesterol**, mmol/L					
Mean ± SD	3.85 ± 1.02	4.20 ± 1.06	4.32 ± 1.08	<0.0001	4.12 ± 1.07
**Lipid-lowering drugs**					
In use, n (col, %)	260 (9.6)	296 (11.1)	315 (11.8)	0.030	871 (10.8)
**Alcohol consumption frequency**, n (col, %)					
Never drinker	172 (6.4)	218 (8.2)	242 (9.1)	<0.001	632 (7.9)
Monthly or less frequent	617 (22.9)	701 (26.5)	799 (30.1)		2,117 (26.5)
2 – 4 times a month	1,146 (42.5)	1,088 (41.1)	1,054 (39.7)		3,288 (41.1)
2 – 3 times a week	596 (22.1)	501 (18.9)	424 (16.0)		1,521 (19.0)
≥ 4 times a week	167 (6.2)	141 (5.3)	133 (5.0)		441 (5.5)
Missing	18	24	26		68
**Daily smoking**, n (col, %)					
Current	387 (14.4)	469 (17.7)	576 (21.8)	<0.001	1,432 (17.9)

For peer review only

For peer review only - http://bmjopen.bmj.com/site/about/guidelines.xhtml

Previously	1,147 (42.6)	1,115 (42.1)	1,171 (44.3)		3,433 (43.0)
Never	1,156 (43.0)	1,066 (40.2)	899 (34.0)		3,121 (39.1)
Missing	26	23	32		81
**Physical activity**, n (col, %)					
Sedentary	352 (13.6)	383 (15.3)	536 (21.6)	<0.001	1,271 (16.8)
Low	1,580 (60.8)	1,532 (61.0)	1,542 (62.2)		4,654 (61.3)
Moderate	589 (22.7)	552 (22.0)	378 (15.2)		1,519 (20.0)
Vigorous	76 (2.9)	44 (1.8)	25 (1.0)		145 (1.9)
Missing	119	162	197		478
**Education**, n (col, %)					
Primary/Secondary	516 (19.2)	641 (24.2)	762 (28.8)	<0.001	1,919 (24.0)
1-2 years senior high school	618 (22.9)	722 (27.3)	746 (28.2)		2,086 (26.1)
High School diploma	220 (8.2)	206 (7.8)	212 (8.0)		638 (8.0)
College/University < 4 years	542 (20.1)	504 (19.0)	479 (18.1)		1,525 (19.1)
College/University ≥ 4 years	799 (29.7)	575 (21.7)	450 (17.0)		1,824 (22.8)
Missing	21	25	29		75

Table 1 – Baseline characteristics of the study participants overall and across hs-CRP tertiles in the Tromsø Study 2007 – 2008.

* p-value across hs-CRP tertiles was obtained by Pearson's chi-squared test for categorial variables, by Kruskal-Wallis test for continuous variables with non-normal distribution, or by one-way ANOVA for continuous variables with normal distribution.

For peer review only

	Tertiles of hs-CRP (mg/L)				
	**Tertile 1** (0.14 – 0.77)	**Tertile 2** (0.78 – 1.69)	**Tertile 3** (1.70 – 9.98)	**p-value**	**Total**
**N**	2,716	2,673	2,678		8,067
New diabetes incidence *, n (%)	52 (1.9)	97 (3.6)	171 (6.4)	<0.001†	320 (4.0)
**Logistic regression, hs-CRP in tertiles**	**OR (p-value, 95% CI)**				
Crude model	1.00 (ref)	1.93 (<0.001, 1.37 – 2.71)	3.49 (<0.001, 2.55 – 4.79)		
Multivariable adjusted ‡	1.00 (ref)	1.21 (0.326, 0.83 – 1.78)	1.73 (0.004, 1.20 – 2.49)		
**Multivariable adjusted model + interaction term††**	**p-value, LRT §**				
+ hs-CRP tertile X Sex	0.902				
+ hs-CRP tertile X BMI	0.807				
+ hs-CRP tertile X hypertension	0.773				
+ hs-CRP tertile X abdominal obesity	0.632				
**Logistic regression, hs-CRP as continuous variable**	**OR (p-value, 95% CI) by 1 log increment of hs-CRP**				
Crude model	1.75 (<0.001, 1.53 – 1.99)				
Multivariable adjusted ‡	1.28 (0.003, 1.09 – 1.50)				

Table 2 – Crude and multivariable adjusted logistic regression models for the association of hs-CRP and diabetes incidences in the Tromsø Study 2007 – 2016.

* Known and unknown diabetes incidence in Tromsø7. † p-value across hs-CRP tertiles by Pearson's chi-squared test. ‡ Adjusted for sex, age, body mass index, abdominal obesity, non-HDL cholesterol, lipid lowering drug use, hypertension, alcohol consumption frequency, daily smoking status, physical activity, and education. †† Models with hs-CRP in teriles adjusted for multivariables as described in ‡, except for variables included as interaction terms. § LRT, Likelihood-ratio test for testing interaction. OR, odds ratio.

For peer review only - http://bmjopen.bmj.com/site/about/guidelines.xhtml

smoking status, physical activity, and education) to correct for any potential confounding effects on the CRP-diabetes association.

Evaluation of potential effect modification - There were conflicting conclusions in previous studies[15 34 38 40-43] on whether CRP-diabetes association is effect modified by sex, BMI, abdominal obesity, or hypertension. In table 2, we examined the potential effect modifiers by adding interaction term for sex (hs-CRP tertile x sex), anthropometric factors (hs-CRP tertile x BMI or hs-CRP tertile x abdominal obesity), or hypertension (hs-CRP tertile x hypertension) separately to the final multivariable logistic regression model and tested the model by likelihood ratio tests (LRT). The P-value of the LRT was used to evaluate whether the addition of any interaction term constitutes a better model. The null hypothesis is that the model is better fit without the addition of interaction term.

Sensitivity analysis - In table 2, the analysis was repeated by using baseline hs-CRP as a continuous independent variable in conducting the logistic regression. Due to its skewed distribution, the baseline hs-CRP in mg/L was firstly log transformed before assessing again its association with diabetes incidence at Tromsø7. Logistic regression, with or without multivariable adjusted for *a priori* covariates as described above, was used to evaluate the odds of diabetes cases by one-log increment of baseline hs-CRP among all study sample.

All analysis was performed using STATA v.14.2 (StataCorp. 2015. Stata Statistical Software: Release 14. College Station, TX: StataCorp LP).

Patient and public involvement

There was no patient involvement in the study. The original cohort data of the two surveys were collected with ethic approvals as described under Ethics Approval section in this paper. Only

For peer review only - http://bmjopen.bmj.com/site/about/guidelines.xhtml

anonymous data from participants who had consented for medical research in the original Tromsø Study were used in this study. The participants from the original Tromsø Study did not participate in the development of the research question of this study nor the outcome measures of this study informed by patients' priorities, experience, and preferences.

For peer review only

For peer review only - http://bmjopen.bmj.com/site/about/guidelines.xhtml

RESULTS

Baseline characteristics

As depicted in table 1, the total study sample of 8,067 individuals had a median hs-CRP level of 1.12 mg/L. The median hs-CRP levels were 0.5 mg/L, 1.12 mg/L, and 2.83 mg/L in tertile 1, 2, 3 respectively. Similarly, a previous study employing a large Norwegian community-based surveys, the Nord-Trøndelag Heath Study (HUNT), reported a median hs-CRP level of 1.1 mg/L among the entire study sample of 30,669 aged from 20 and older.[59]

The baseline characteristics (waist-to-height ratio, abdominal obesity, non-HDL cholesterol, systolic/diastolic blood pressure, hypertension, blood pressure lowering drugs, daily smoking, alcohol consumption frequency, BMI, physical activity, and education) of the participants exhibited statistical evidence for a difference across the 3 tertiles with increasing values of hs-CRP (p-values <0.001 – <0.0001) (table 1). Conversely, lipid-lowering drug usage only showed modest impact (p-value = 0.03). In tertiles 2 and 3, 55% and 60.5% of the participants were aged ≥ 55 years, respectively, while 82% and 90% of these participants were categorized as abdominal obesity. In addition, participants in tertiles 2 and 3 had higher percentage of overweight or obesity, hypertension, physically inactive, daily smokers, lower education, and never or infrequent drinking when compared with those in tertile 1.

Age-standardized cumulative diabetes incidence

There was a more than 3-fold increase of overall age-standardized cumulative diabetes incidence in the highest hs-CRP tertile 3 (6.3%) when compared with the reference tertile 1 (2.0%). This trend was apparent in both women (1.5% vs 5.8%, 3.9-fold increase) and men (2.8% vs 7.2%, 2.6-fold increase) (figure 3).

Baseline hs-CRP associates with future diabetes development

For peer review only - http://bmjopen.bmj.com/site/about/guidelines.xhtml

There were 320 (4.0%) known and unknown incident cases of diabetes during follow-up (table 2). The odds of diabetes incidence were almost 2 times (crude OR=1.93; p-value <0.001; 95% CI 1.37 – 2.71) and 3.5 times (crude OR=3.49; p-value <0.001; 95% CI 2.55 – 4.79) higher among participants of hs-CRP tertile 2 and tertile 3, respectively. Although the strength of the association reduced in the fully adjusted model, positive association remained and the participants in hs-CRP tertile 3 had 73% higher odds of future diabetes (OR = 1.73; p-value, 0.004; 95% CI, 1.20 – 2.49). The addition of the individual interaction term of hs-CRP tertile x sex (Likelihood-ratio tests (LRT) p-value, 0.902), hs-CRP tertile x BMI (LRT p-value, 0.807), hs-CRP tertile x hypertension (LRT p-value, 0.773), or hs-CRP tertile x abdominal obesity (LRT p-value, 0.632) to the multivariable adjusted model did not support any compound effects of these covariates (table 2).

Sensitivity analysis

In the sensitivity analysis, consistent with the analyses using hs-CRP tertiles, a positive association between log transformed hs-CRP and diabetes development was observed (table 2). For every one-log baseline hs-CRP increment, there was 75% increase in odds of diabetes cases (crude OR = 1.75; p-value, <0.001; 95% CI 1.53 – 1.99). Positive association maintained after multivariable adjustment with 28% higher odds of diabetes development (OR = 1.28; p-value, 0.003; 95% CI 1.09 – 1.50) per one-log increase of baseline hs-CRP.

For peer review only - http://bmjopen.bmj.com/site/about/guidelines.xhtml

DISCUSSION

We found a marked association between high hs-CRP and future diabetes incidence at 7-year follow-up in this study of adults from a general Norwegian population.

Comparison to other studies

A prospective study conducted in Spain demonstrated a positive association between elevated hs-CRP with future diabetes development after adjusting for age, sex, and obesity[36] but a study in UK found that the association was attenuated and no longer significant after adjusting for BMI and waist-to-hip ratio.[39] While some others showed that the association became insignificant in men but maintained in women after adjusting for BMI, suggesting evidence of interaction between hs-CRP and sex on the association.[34 40] However, the present study did not observe evidence for interaction between hs-CRP and sex, BMI, abdominal obesity, or hypertension. The association was also maintained after adjusting for multiple factors including BMI and abdominal obesity. Whereas some other studies[34 36] did not exclude individuals with elevated baseline CRP levels above 10 mg/L, which might have uplifted the exposure level due to the potential underlying issues of acute inflammatory condition other than the risk associated with diabetes development. This heightened exposure threshold likely attenuated the true association of the exposure and outcome. A Finnish cohort study[40] relied primarily on the diagnosed diabetes data through medical registries and self-reported surveys, whereas a German case-control study[34] identified baseline diabetes solely by self-reported information. There might be limitation of misclassifying baseline diabetes due to bias on self-reported diabetes. The lack of validated laboratory-based tests such as HbA1c levels or fasting-blood sugar content in these studies might fail to capture any unknown diabetes at baseline, which might have led to non-differential misclassification and could have diluted the strength of the association. Whereas, in the German study, the differential misclassification of the

For peer review only - http://bmjopen.bmj.com/site/about/guidelines.xhtml

baseline unknown diabetes among the controls might have biased the association leaning towards null due to the case-control study design,[34] which in part might be explaining the attenuation in the strength of association after adjusting for BMI. Lee and colleagues,[39] on the other hand, used a HbA1c level cutoff of >7.0% (i.e., >53 mmol/mol) for identifying unknown diabetes status at baseline and during follow-up. While this higher cutoff had been used for defining diabetes status, this deviation from the cutoff of 6.5% (i.e., 48 mmol/mol) recommended by the World Health Organization (WHO)[60] could introduce inconsistencies with the findings of other studies which chose to follow the WHO recommended standards.

There is increasing awareness that elevated hs-CRP levels and diabetes incidences are associated with adiposity.[26] The association of elevated hs-CRP with central adiposity could be independent of BMI.[61] Some European studies adjusted their models for BMI but not for central adiposity.[34 36 40] Waist-to-hip ratio has been indicated using various sex-specific thresholds.[62] Although Lee and colleagues had adjusted for both BMI and waist-to-hip ratio,[39] the inherent limitation of the waist-to-hip ratio might have led to residual confounding in some of these published analyses. Indeed, Lee and colleagues suggested that the differential results found in a meta-analysis of 16 published studies might be due to, in part, the adjustment for different indexes of central adiposity.[39] The waist-to-height ratio had been found to be sex-, age-, and ethnicity-independent;[62] and the use of this index in the current study might have addressed more adequately the confounding impact of the fat storage profile for both men and women.

Aging-associated chronic inflammation has been proposed as playing a part in the etiology of type 2 diabetes.[63] Although aging has long been identified as a risk factor and positively correlates with pathogenesis, it would be challenging to pinpoint a particular age cutoff to inform clinical practice. Previous research have suggested multiple sources of aging-associated chronic inflammation

For peer review only - http://bmjopen.bmj.com/site/about/guidelines.xhtml

including age-related accumulation of damaged cells, changes in gut microbiota over age, increasing cellular senescence and the subsequent secretion of proinflammatory cytokines namely the senescence-associated secretory phenotype or SASP, and age-associated modification of the immune system among others.[63] Although fat is a good source of inflammatory cytokines, obesity alone may not represent the entire spectrum of mechanisms underlying the development of diabetes via chronic systemic inflammation.

Individuals deemed prediabetes, defined by HbA1c between 5.7 – 6.4% or 39 – 47 mmol/ml, were included in this current study. Prediabetes state has been considered as risk for diabetes transformation.[64] Although positive association had been previously implicated,[65 66] the temporal relationship between elevated hs-CRP and prediabetes condition remained unclear. Glycemic parameters such as HbA1c could provide estimates to reflect the average blood glucose concentration of an individual over the preceding 2 to 3 months. Nevertheless, the method also elicits certain extent of inaccuracies due to some glucose-independent variation, although corrective modeling has recently been proposed.[67]

Strengths of the study

This study has several strengths. The 7-year follow-up study allowed us to investigate the temporal association of hs-CRP on diabetes development. The study population was recruited and sampled from all residents of the Tromsø municipality with validated systematic data collection for each of the repetitive surveys, including the questionnaires, the clinical examinations and the lab assessments. The data being analyzed were of high quality with high participation in the study, and low levels of missing data in general (<11%) – which garners high internal validity. No findings from similar population-based studies conducted in the Scandinavian countries investigating the association between CRP and incident diabetes have been previously reported.

For peer review only - http://bmjopen.bmj.com/site/about/guidelines.xhtml

The current findings of the positive CRP-diabetes association may inform public health strategies for prevention and surveillance of diabetes in the Nordic region where lifestyle, culture, demography, health care and socioeconomic factors resemble Norway.

The data available in the current analysis included the information of the use of lipid-lowering drugs, which are used in primary and secondary prevention of cardiovascular diseases but have previously been shown capable in reducing hs-CRP levels but at the same time might contribute to drug-induced side effects of impaired glycemic control or new onset of diabetes.[31] These features might have led to residual confounding in some other previous studies which were not able to or did not include medication use in their multivariable adjusted models.[15 33] Moreover, the current study had utilized a large overall cohort sample size.

Limitations of the study

Limitations of this study include the lack of information of diabetes subtype. Since the study sample only included adults 30 years of age and above, primarily newly developed type 2 diabetes were expected. The study focused on a Norwegian general population and hence the conclusion may not be generalizable beyond this region. Since the baseline and future developed diabetes cases were self-reported, there might be potential non-differential misclassification of the outcome due to reporting bias. Similarly, self-reported medication for treating symptoms did not come with physician, medical record, or medical registry confirmation might have led to potential measurement error in the outcome. Nevertheless, previous studies have found that self-reported diabetes[68-70] often accounted for high specificity (84.5% to 99.7%) although it can exhibit low sensitivity (41.5% to 80.4%). Previous studies had reported the impact from socioeconomic position and demographic characteristics such that the sensitivity of self-reported diabetes can be elevated with higher education levels and among urban dwellers.[70] In the current study, validated

For peer review only - http://bmjopen.bmj.com/site/about/guidelines.xhtml

laboratory-based measurement of the HbA1c level was utilized in addition to self-reported diabetes and diabetes medication use for treating symptoms to enhance both the sensitivity and specificity when capturing the known and unknown true positive and false negative diabetes cases.

We did not include information on diet, which is another potential confounder. Diet can be negatively or positively associated with hs-CRP level and with future diabetes development depending on the diet types and patterns.[71] It would be of interest to include this parameter in future studies. In addition, diet is also linked with socioeconomic status which was apparently correlated with hs-CRP level as depicted in this report. On the other hand, due to data sparsity, we had combined participants with underweight condition (< 18.5 kg/m^2) with those with normal BMI 18.5 - 24.9 kg/m^2) as a single entity “normal BMI (< 25 kg/m^2)” in the model. Given that the overweight and obesity conditions were more likely associated with hs-CRP and/or diabetes development,[12 25-27] the grouping of the small number of underweight with the normal BMI participants should not introduce impactful confounding effects in the model.

As chronic inflammation relates to the etiology of type 1 diabetes, it would be of interest to study its association with hs-CRP by utilizing a more defined type 1 diabetes cohort. It is also of interest to include hormone replacement therapy[72 73] as a potential confounder since this medication for treating symptoms had been implicated associated with elevated hs-CRP levels but also implicated in the reduction of abdominal fat.

For peer review only - http://bmjopen.bmj.com/site/about/guidelines.xhtml

CONCLUSION

In conclusion, this study has demonstrated that hs-CRP is associated with future diabetes development in a population-based sample of Norwegian adults. This association was consistent across sex, body mass index, abdominal obesity and hypertension. In combination with glycemic parameters such as HbA1c or fasting plasma glucose, this stable blood-based biomarker can potentially be used to facilitate risk group stratification, which may provide opportunities for early prevention or intervention of diabetes through clinical practices, public health education, and anti-inflammatory therapeutics development. Previous studies[73 74] had depicted ethnic variations of CRP levels which we were not able to explore within this study. When comparing with white, after multivariable adjustment including BMI, black individuals had higher serum CRP concentrations while Asians including Chinese and Japanese had lower levels. Future studies to determine the potentially ethnicity-dependent cutoffs of hs-CRP for risk stratification are warranted.

For peer review only

For peer review only - http://bmjopen.bmj.com/site/about/guidelines.xhtml

ACKNOWLEGMENTS

We would like to thank the participants of the Tromsø Study. We would like to extend our gratitude to Dr. Anne Elise Eggen, Dr. Ola Løvsletten, Ms. Julie-Helene D. Sørensen, and the members of the Tromsø Study Data and Publication Committee of UiT The Arctic University of Norway to make the Tromsø dataset available for this study.

For peer review only

For peer review only - http://bmjopen.bmj.com/site/about/guidelines.xhtml

CONTRIBUTORS

K.I.T. conceived the research question of the present study based on the suggestion from S.C. on a project relating to diabetes and the Tromsø Study. K.I.T. planned out the study design, analyzed and interpreted the data, constructed figures and tables, wrote the manuscript. L.A.H. and S.C. contributed to the discussion of the research question, study design, analysis, and data interpretation. L.A.H. and S.C. took part in the critical revision of the manuscript and provided overall supervision of the study. S.C. coordinated the collaboration of the study. L.A.H took part in the data collection of Tromsø6 and Tromsø7. L.A.H. took part in the planning and management of Tromsø7. K.I.T. is the guarantor of this work and, as such, had full access to all the data in the study and takes responsibility for the integrity of the data and the accuracy of the data analysis. All authors approved the final manuscript.

For peer review only

For peer review only - http://bmjopen.bmj.com/site/about/guidelines.xhtml

COMPETING INTERESTS

Authors declare no potential conflicts of interest relevant to this article.

For peer review only

For peer review only - http://bmjopen.bmj.com/site/about/guidelines.xhtml

ETHICS APPROVAL

Tromsø6 was approved by the Data Inspectorate of Norway and the Regional Committee for Medical and Health Research Ethics Northern Norway (REC North) (Ref. 121/2006) and Tromsø7 by the Norwegian Data Protection Authority (Ref. 14/01463-4/CGN) and REC North (Ref. 2014/940). The current study was approved by the London School of Hygiene & Tropical Medicine Ethics Committee (Ref. 2566).

For peer review only

For peer review only - http://bmjopen.bmj.com/site/about/guidelines.xhtml

DATA AVAILABILITY STATEMENT

Data are not publicly available but may be obtained from UiT The Arctic University of Norway through application and project approval by the Data and Publication Committee. The data used in this study are owned and managed by UiT The Arctic University of Norway (https://uit.no/research/tromsostudy).

For peer review only

FUNDING

Funding for the Tromsø Study was obtained from UiT The Arctic University of Norway, Northern Norway Regional Health Authority, Ministry of Health and Care Services, Norwegian Research Council, and various public and charity research funds in Norway. There was no external funding for this specific paper. The funding bodies have no role in the design of the study, data collection, analysis, interpretation of data, or in writing the manuscript.

For peer review only

For peer review only - http://bmjopen.bmj.com/site/about/guidelines.xhtml

REFERENCES

1 Saeedi P, Petersohn I, Salpea P, et al. Global and regional diabetes prevalence estimates for 2019 and projections for 2030 and 2045: Results from the International Diabetes Federation Diabetes Atlas, 9th edition. *Diabetes Research and Clinical Practice* 2019;157:107843.
2 Schwartz SS, Epstein S, Corkey BE, *et al*. The Time Is Right for a New Classification System for Diabetes: Rationale and Implications of the β-Cell-Centric Classification Schema. *Diabetes Care* 2016;39:179–86.
3 Classification of diabetes mellitus. Geneva: World Health Organization. 2019. Available: https://www.who.int/publications/i/item/classification-of-diabetes-mellitus [Accessed 11 Sept 2021].
4 Shoelson SE, Lee J, Goldfine AB. Inflammation and insulin resistance. *J Clin Invest* 2006;116:1793–801.
5 Clark M, Kroger CJ, Tisch RM. Type 1 Diabetes: A chronic Anti-self-inflammatory response. *Front Immunol* 2017;8:1898.
6 Medzhitov R. Origin and physiological roles of inflammation. *Nature* 2008;454:428–35.
7 Castell JV, Gómez-Lechón MJ, David M, *et al*. Acute-phase response of human hepatocytes: regulation of acute-phase protein synthesis by interleukin-6. *Hepatology* 1990;12:1179–86.
8 Chen L, Deng H, Cui H, *et al*. Inflammatory responses and inflammation-associated diseases in organs. *Oncotarget* 2018;9:7204–18.
9 Donath MY. Targeting inflammation in the treatment of type 2 diabetes: time to start. *Nat Rev Drug Discov* 2014;13(6):465-76.
10 Tsalamandris S, Antonopoulos AS, Oikonomou E, *et al*. The Role of Inflammation in Diabetes: Current Concepts and Future Perspectives. *Eur Cardiol* 2019;14:50–9.
11 Guarner V, Rubio-Ruiz ME. Low-grade systemic inflammation connects aging, metabolic syndrome and cardiovascular disease. *Interdiscip Top Gerontol* 2015;40:99–106.
12 Visser M, Bouter LM, McQuillan GM, *et al*. Elevated C-reactive protein levels in overweight and obese adults. *JAMA* 1999;282:2131–5.
13 Fröhlich M, Sund M, Löwel H, *et al*. Independent association of various smoking characteristics with markers of systemic inflammation in men. Results from a representative sample of the general population (MONICA Augsburg Survey 1994/95). *Eur Heart J* 2003;24:1365–72.
14 Yu Z, Ye X, Wang J, *et al*. Associations of physical activity with inflammatory factors, adipocytokines, and metabolic syndrome in middle-aged and older chinese people. *Circulation* 2009;119:2969–77.
15 Doi Y, Kiyohara Y, Kubo M, *et al*. Elevated C-reactive protein is a predictor of the development of diabetes in a general Japanese population: the Hisayama Study. *Diabetes Care* 2005;28:2497–500.
16 Chase HP, Cooper S, Osberg I, *et al*. Elevated C-reactive protein levels in the development of type 1 diabetes. *Diabetes* 2004;53:2569–73.
17 Khera A, McGuire DK, Murphy SA, *et al*. Race and gender differences in C-reactive protein levels. *J Am Coll Cardiol* 2005;46:464–9.

For peer review only - http://bmjopen.bmj.com/site/about/guidelines.xhtml

18 Kautzky-Willer A, Harreiter J, Pacini G. Sex and Gender Differences in Risk, Pathophysiology and Complications of Type 2 Diabetes Mellitus. *Endocr Rev* 2016;37:278–316.
19 Kirkman MS, Briscoe VJ, Clark N, *et al*. Diabetes in older adults. *Diabetes Care* 2012;35:2650–64.
20 Friedman EM, Herd P. Income, education, and inflammation: differential associations in a national probability sample (The MIDUS study). *Psychosom Med* 2010;72:290–300.
21 Imhof A, Froehlich M, Brenner H, *et al*. Effect of alcohol consumption on systemic markers of inflammation. *Lancet* 2001;357:763–7.
22 Carlsson S, Hammar N, Grill V, *et al*. Alcohol consumption and the incidence of type 2 diabetes: a 20-year follow-up of the Finnish twin cohort study. *Diabetes Care* 2003;26:2785–90.
23 Sargeant LA, Khaw KT, Bingham S, *et al*. Cigarette smoking and glycaemia: the EPIC-Norfolk Study. European Prospective Investigation into Cancer. *Int J Epidemiol* 2001;30:547–54
24 Wahid A, Manek N, Nichols M, *et al*. Quantifying the Association Between Physical Activity and Cardiovascular Disease and Diabetes: A Systematic Review and Meta-Analysis. *J Am Heart Assoc* 2016;5:e002495.
25 Narayan KM, Boyle JP, Thompson TJ, *et al*. Effect of BMI on lifetime risk for diabetes in the U.S. *Diabetes Care* 2007;30:1562–6.
26 Lebovitz HE, Banerji MA. Visceral adiposity is causally related to insulin resistance. *Diabetes Care* 2005;28:2322–25.
27 Ashwell M, Gunn P, Gibson S. Waist-to-height ratio is a better screening tool than waist circumference and BMI for adult cardiometabolic risk factors: systematic review and meta-analysis. *Obes Rev* 2012;13:275–86.
28 Jovicić S, Ignjatović S, Dajak M, *et al*. Association of lipid and inflammatory markers with C-reactive protein in cardiovascular risk assessment for primary prevention. *Clin Lab* 2009;55:411–9.
29 Seo MH, Bae JC, Park SE, *et al*. Association of lipid and lipoprotein profiles with future development of type 2 diabetes in nondiabetic Korean subjects: a 4-year retrospective, longitudinal study. *J Clin Endocrinol Metab* 2011;96:E2050–4.
30 Albert MA, Danielson E, Rifai N, *et al*. Effect of statin therapy on C-reactive protein levels: the pravastatin inflammation/CRP evaluation (PRINCE): a randomized trial and cohort study. *JAMA* 2001;286:64–70.
31 Kosmas CE, Silverio D, Sourlas A, *et al*. Impact of lipid-lowering therapy on glycemic control and the risk for new-onset diabetes mellitus. *Drugs Context* 2018;7:212562.
32 Lakoski SG, Cushman M, Palmas W, *et al*. The relationship between blood pressure and C-reactive protein in the Multi-Ethnic Study of Atherosclerosis (MESA). *J Am Coll Cardiol* 2005;46:1869–74.
33 Thorand B, Löwel H, Schneider A, *et al*. C-reactive protein as a predictor for incident diabetes mellitus among middle-aged men: results from the MONICA Augsburg cohort study, 1984-1998. *Arch Intern Med* 2003;163:93–9.
34 Thorand B, Baumert J, Kolb H, *et al*. Sex differences in the prediction of type 2 diabetes by inflammatory markers: results from the MONICA/KORA Augsburg case-cohort study, 1984-2002. *Diabetes Care* 2007;30:854–60.

For peer review only - http://bmjopen.bmj.com/site/about/guidelines.xhtml

35 Duncan BB, Schmidt MI, Pankow JS, *et al*. Low-grade systemic inflammation and the development of type 2 diabetes: the atherosclerosis risk in communities study. *Diabetes* 2003;52:1799–805.
36 Rubio-Martín E, Soriguer F, Gutiérrez-Repiso C, *et al*. C-reactive protein and incidence of type 2 diabetes in the Pizarra study. *Eur J Clin Invest* 2013;43:159–67.
37 Pradhan AD, Manson JE, Rifai N, *et al*. C-reactive protein, interleukin 6, and risk of developing type 2 diabetes mellitus. *JAMA* 2001;286:327–34.
38 Effoe VS, Correa A, Chen H, *et al*. High-Sensitivity C-Reactive Protein Is Associated With Incident Type 2 Diabetes Among African Americans: The Jackson Heart Study. *Diabetes Care* 2015;38:1694–700.
39 Lee CC, Adler AI, Sandhu MS, *et al*. Association of C-reactive protein with type 2 diabetes: prospective analysis and meta-analysis. *Diabetologia* 2009;52:1040–7.
40 Hu G, Jousilahti P, Tuomilehto J, *et al*. Association of serum C-reactive protein level with sex-specific type 2 diabetes risk: a prospective finnish study. *J Clin Endocrinol Metab* 2009;94:2099–105.
41 Kanmani S, Kwon M, Shin MK, *et al*. Association of C-Reactive Protein with Risk of Developing Type 2 Diabetes Mellitus, and Role of Obesity and Hypertension: A Large Population-Based Korean Cohort Study. *Sci Rep* 2019;9:4573.
42 Pan A, Wang Y, Yuan JM, *et al*. High-sensitive C-reactive protein and risk of incident type 2 diabetes: a case-control study nested within the Singapore Chinese Health Study. *BMC Endocr Disord* 2017;17:8.
43 Ahmadi-Abhari S, Kaptoge S, Luben RN, *et al*. Longitudinal association of C-reactive protein and Haemoglobin A1c over 13 years: the European Prospective Investigation into Cancer-Norfolk study. *Cardiovasc Diabetol* 2015;14:61.
44 Hovland A, Retterstøl K, Mollnes TE, *et al*. Anti-inflammatory effects of non-statin low-density lipoprotein cholesterol-lowering drugs: an unused potential? *Scand Cardiovasc J* 2020;54:274–9.
45 Oh RC, Lanier JB. Management of hypertriglyceridemia. *Am Fam Physician* 2007;75:1365–71.
46 Beshara A, Cohen E, Goldberg E, *et al*. Triglyceride levels and risk of type 2 diabetes mellitus: a longitudinal large study. *J Investig Med* 2016;64:383–7.
47 Overweight and obesity in Norway. Available: https://www.fhi.no/en/op/hin/health-disease/overweight-and-obesity-in-norway---/ [Accessed 19 Mar 2023].
48 Alberti KGMM, Eckel RH, Grundy SM, *et al*. Harmonizing the metabolic syndrome: a joint interim statement of the International Diabetes Federation Task Force on Epidemiology and Prevention; National Heart, Lung, and Blood Institute; American Heart Association; World Heart Federation; International Atherosclerosis Society; and International Association for the Study of Obesity. *Circulation* 2009;120:1640-5.
49 Michalsen VL Kvaløy K, Svartberg J, *et al*. Change in prevalence and severity of metabolic syndrome in the Sami and non-Sami population in rural Northern Norway using a repeated cross-sectional population-based study design: the SAMINOR Study. *BMJ Open* 2019;9:e027791.
50 Jacobsen BK, Eggen AE, Mathiesen EB, *et al*. Cohort profile: the Tromsø Study. *Int J Epidemiol* 2012;41:961–7.
51 Eggen AE, Mathiesen EB, Wilsgaard T, *et al*. The sixth survey of the Tromsø Study (Tromsø6) in 2007-08: collaborative research in the interface between clinical medicine and

For peer review only

For peer review only - http://bmjopen.bmj.com/site/about/guidelines.xhtml

epidemiology: study objectives, design, data collection procedures, and attendance in a multipurpose population-based health survey. *Scand J Public Health* 2013;41:65–80.
52 The seventh survey of the Tromsø Study. Available: https://uit.no/research/tromsostudy/project?pid=708909 [Accessed 11 Sept 2021].
53 Håheim LL, Nafstad P, Olsen I, *et al*. C-reactive protein variations for different chronic somatic disorders. *Scand J Public Health* 2009;37:640-6.
54 Langholz PL, Wilsgaard T, Njølstad I, *et al*. Trends in known and undiagnosed diabetes, HbA1c levels, cardiometabolic risk factors and diabetes treatment target achievement in repeated cross-sectional surveys: the population-based Tromsø Study 1994-2016. *BMJ Open* 2021;11:e041846.
55 WHO collaborating centre for drug statistics methodology, Guidelines for ATC classification and DDD assignment, 2022. Available: https://www.whocc.no/filearchive/publications/2022_guidelines_web.pdf [Accessed 10 Jan 2022].
56 Browning LM, Hsieh SD, Ashwell M. A systematic review of waist-to-height ratio as a screening tool for the prediction of cardiovascular disease and diabetes: 0·5 could be a suitable global boundary value. *Nutr Res Rev* 2010;23:247–69.
57 Raised blood pressure (Indicator 11). Available: https://www.fhi.no/en/op/Indicators-for-NCD/blood-pressure/hoyt-blodtrykk-indikator-11/ [Accessed 19 Mar 2023]
58 Pace M LG, Glickman M, Grande E, *et al*. Revision of the European Standard Population, Report of Eurostat's task force, 2013 edition. Available: https://ec.europa.eu/eurostat/documents/3859598/5926869/KS-RA-13-028-EN.PDF/e713fa79-1add-44e8-b23d-5e8fa09b3f8f [Accessed 11 Sept 2021].
59 Ho KKN, Simic M, Småstuen MC, *et al*. The association between insomnia, c-reactive protein, and chronic low back pain: cross-sectional analysis of the HUNT study, Norway. *Scand J* Pain 2019;19:765–77.
60 Use of glycated haemoglobin (HbA1c) in the diagnosis of diabetes mellitus. Abbreviated report of a WHO consultation. Available: https://apps.who.int/iris/bitstream/handle/10665/70523/WHO_NMH_CHP_CPM_11.1_eng.pdf?sequence=1&isAllowed=y [Accessed 11 Sept 2021].
61 Lapice E, Maione S, Patti L, *et al*. Abdominal adiposity is associated with elevated C-reactive protein independent of BMI in healthy nonobese people. *Diabetes Care* 2009;32:1734–36.
62 Yoo EG. Waist-to-height ratio as a screening tool for obesity and cardiometabolic risk. *Korean J Pediatr* 2016;59:425–31.
63 Franceschi C, Campisi J. Chronic inflammation (Inflammaging) and its potential contribution to age-associated diseases. *J Gerontol A Biol Sci Med Sci* 2014;69(Suppl 1):S4–9.
64 Tabák AG, Herder C, Rathmann W, *et al*. Prediabetes: A high-risk state for developing diabetes. *Lancet* 2012; 379:2279–90.
65 Kato K, Otsuka T, Saiki Y, *et al*. Association between elevated C-reactive protein levels and prediabetes in adults, particularly impaired glucose tolerance. *Can J Diabetes* 2019;43:40–5.
66 Jaiswal A, Tabassum R, Podder A, *et al*. Elevated level of C-reactive protein is associated with risk of prediabetes in Indians. *Atherosclerosis* 2012;222:495–501.
67 Malka R, Nathan DM, Higgins JM. Mechanistic modeling of hemoglobin glycation and red blood cell kinetics enables personalized diabetes monitoring. *Sci Transl Med* 2016;8:359ra130.

For peer review only - http://bmjopen.bmj.com/site/about/guidelines.xhtml

68 Okura Y, Urban LH, Mahoney DW, *et al*. Agreement between self-report questionnaires and medical record data was substantial for diabetes, hypertension, myocardial infarction and stroke but not for heart failure. *J Clin Epidemiol* 2004;57:1096–103.
69 Schneider ALC, Pankow JS, Heiss G, *et al*. Validity and reliability of self-reported diabetes in the atherosclerosis risk in communities study. *Am J Epidemiol* 2012;176:738–43.
70 Yuan X, Liu T, Wu L, *et al*. Validity of self-reported diabetes among middle-aged and older Chinese adults: the China health and retirement longitudinal study. *BMJ Open* 2015;5:e006633.
71 van Woudenbergh GJ, Kuijsten A, Tigcheler B, *et al*. Meat consumption and its association with C-reactive protein and incident type 2 diabetes: the Rotterdam Study. *Diabetes Care* 2012;35:1499–505.
72 Pradhan AD, Manson JE, Rossouw JE, *et al*. Inflammatory biomarkers, hormone replacement therapy, and incident coronary heart disease: prospective analysis from the Women's Health Initiative observational study. *JAMA* 2002;288:980–7.
73 Albert MA, Glynn RJ, Burling J, *et al*. C-reactive protein levels among women of various ethnic groups living in the United States (from the Women's Health Study). *Am J Cardiol* 2004;93:1238–42.
74 Kelley-Hedgepeth A, Lloyd-Jones DM, Colvin A, *et al*. Ethnic differences in C-reactive protein concentrations. *Clin Chem* 2008;54:1027–37

For peer review only

For peer review only - http://bmjopen.bmj.com/site/about/guidelines.xhtml

FIGURE LEGENDS

Figure 1 – Conceptual framework.

Figure 2 – Study sample inclusion flow chart of the Tromsø Study 2007 – 2016.

Figure 3 – Age-standardized cumulative diabetes incidence across hs-CRP tertiles during follow-up in the Tromsø Study 2015 – 2016.

For peer review only

For peer review only - http://bmjopen.bmj.com/site/about/guidelines.xhtml

Figure 1

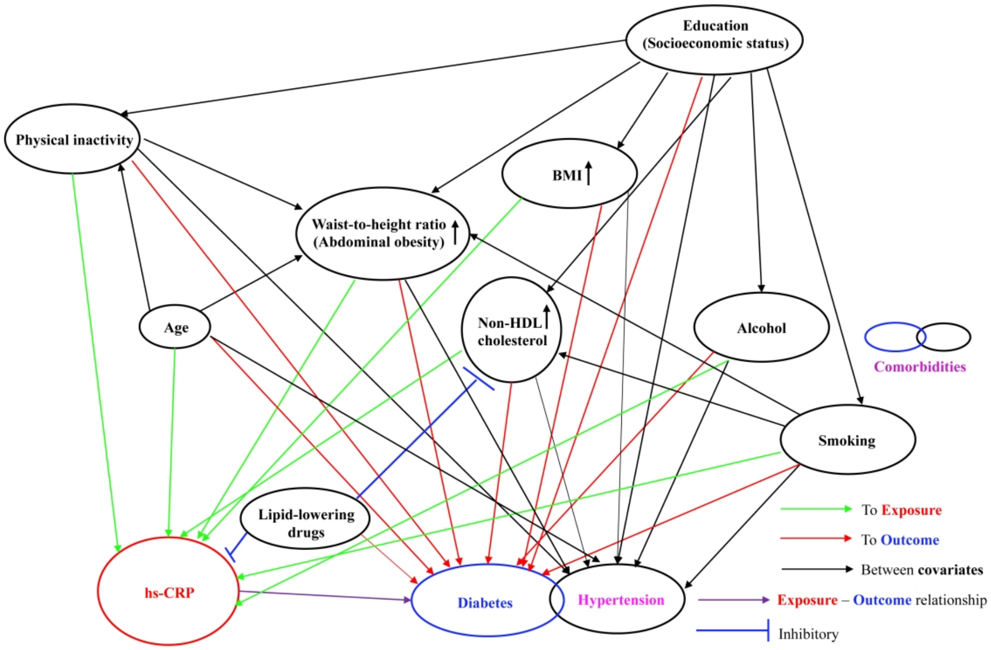

Figure 1. Conceptual framework

280x199mm (300 x 300 DPI)

Figure 2

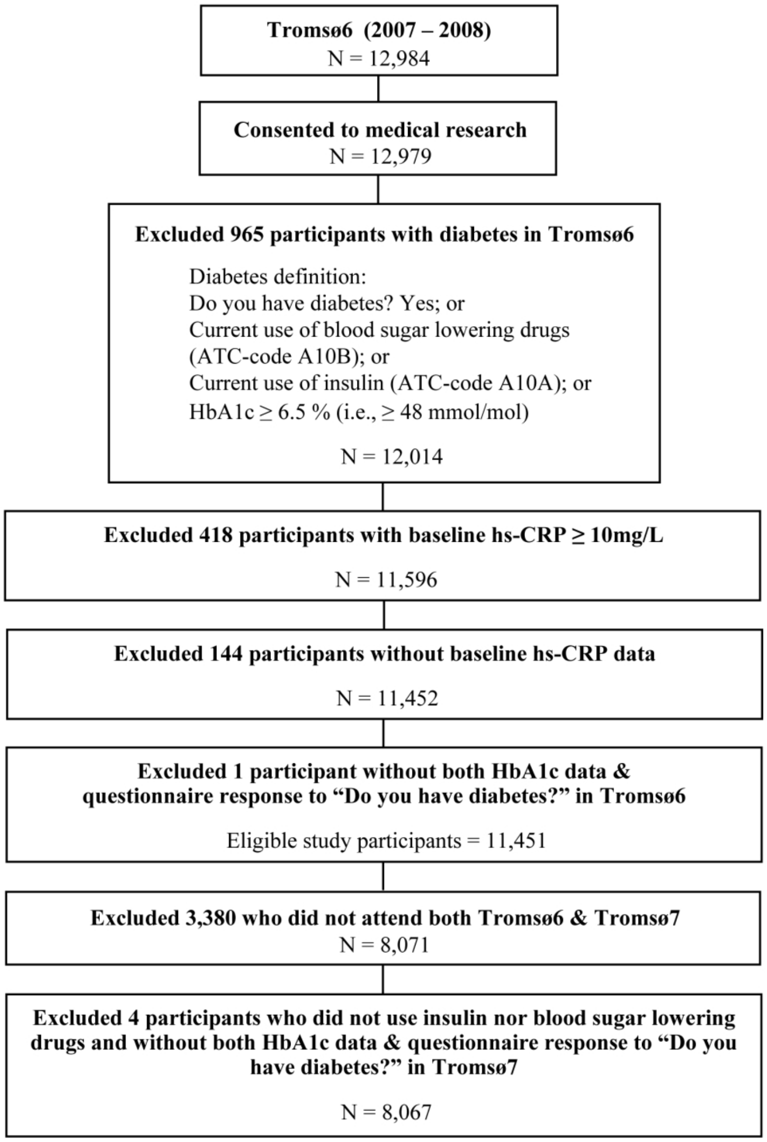

Figure 2. Study sample inclusion flow chart of the Tromsø Study 2007 – 2016

135x206mm (300 x 300 DPI)

Figure 3

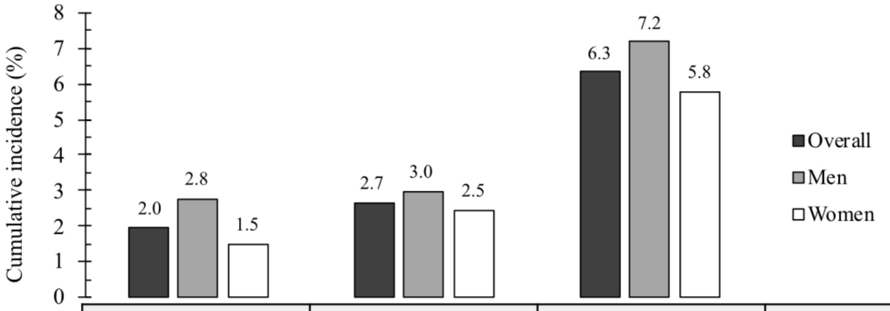

Tertile 1 (0.14 – 0.77)	Tertile 2 (0.78 – 1.69)	Tertile 3 (1.70 – 9.98)	hs-CRP (mg/L)
n=2,716	n=2,673	n=2,678	Overall (n=8,067)
n=1,139	n=1,336	n=1,229	Men (n=3,704)
n=1,577	n=1,337	n=1,449	Women (n=4,363)

Figure 3. Age-standardized cumulative diabetes incidence across hs-CRP tertiles during follow-up in the Tromsø Study 2015 – 2016

187x99mm (300 x 300 DPI)

STROBE Statement—Checklist of items that should be included in reports of ***cohort studies***

	Item No	Recommendation	Page No
**Title and abstract**	1	(*a*) Indicate the study's design with a commonly used term in the title or the abstract	2
		(*b*) Provide in the abstract an informative and balanced summary of what was done and what was found	2
**Introduction**			
Background/rationale	2	Explain the scientific background and rationale for the investigation being reported	4-5
Objectives	3	State specific objectives, including any prespecified hypotheses	5
**Methods**			
Study design	4	Present key elements of study design early in the paper	6
Setting	5	Describe the setting, locations, and relevant dates, including periods of recruitment, exposure, follow-up, and data collection	6-7
Participants	6	(*a*) Give the eligibility criteria, and the sources and methods of selection of participants. Describe methods of follow-up	Figure 1
		(*b*) For matched studies, give matching criteria and number of exposed and unexposed	N/A
Variables	7	Clearly define all outcomes, exposures, predictors, potential confounders, and effect modifiers. Give diagnostic criteria, if applicable	7-9
Data sources/ measurement	8*	For each variable of interest, give sources of data and details of methods of assessment (measurement). Describe comparability of assessment methods if there is more than one group	7-9
Bias	9	Describe any efforts to address potential sources of bias	7-9
Study size	10	Explain how the study size was arrived at	6, Figure 1
Quantitative variables	11	Explain how quantitative variables were handled in the analyses. If applicable, describe which groupings were chosen and why	9
Statistical methods	12	(*a*) Describe all statistical methods, including those used to control for confounding	9-10
		(*b*) Describe any methods used to examine subgroups and interactions	10
		(*c*) Explain how missing data were addressed	10
		(*d*) If applicable, explain how loss to follow-up was addressed	6
		(*e*) Describe any sensitivity analyses	10
**Results**			
Participants	13*	(a) Report numbers of individuals at each stage of study—eg numbers potentially eligible, examined for eligibility, confirmed eligible, included in the study, completing follow-up, and analysed	Page 11/Figure 1
		(b) Give reasons for non-participation at each stage	Figure 1
		(c) Consider use of a flow diagram	Figure 1
Descriptive data	14*	(a) Give characteristics of study participants (eg demographic, clinical, social) and information on exposures and potential confounders	11
		(b) Indicate number of participants with missing data for each variable of interest	Table 1
		(c) Summarise follow-up time (eg, average and total amount)	Fixed follow up time –

For peer review only - http://bmjopen.bmj.com/site/about/guidelines.xhtml

			explained page 6
Outcome data	15*	Report numbers of outcome events or summary measures over time	Table 2 & Figure 2

For peer review only

Main results	16	(*a*) Give unadjusted estimates and, if applicable, confounder-adjusted estimates and their precision (eg, 95% confidence interval). Make clear which confounders were adjusted for and why they were included	Table 2
		(*b*) Report category boundaries when continuous variables were categorized	Table 2
		(*c*) If relevant, consider translating estimates of relative risk into absolute risk for a meaningful time period	n/a
Other analyses	17	Report other analyses done—eg analyses of subgroups and interactions, and sensitivity analyses	12
**Discussion**			
Key results	18	Summarise key results with reference to study objectives	13
Limitations	19	Discuss limitations of the study, taking into account sources of potential bias or imprecision. Discuss both direction and magnitude of any potential bias	16,17
Interpretation	20	Give a cautious overall interpretation of results considering objectives, limitations, multiplicity of analyses, results from similar studies, and other relevant evidence	17
Generalisability	21	Discuss the generalisability (external validity) of the study results	16,17
**Other information**			
Funding	22	Give the source of funding and the role of the funders for the present study and, if applicable, for the original study on which the present article is based	23

*Give information separately for exposed and unexposed groups.

Note: An Explanation and Elaboration article discusses each checklist item and gives methodological background and published examples of transparent reporting. The STROBE checklist is best used in conjunction with this article (freely available on the Web sites of PLoS Medicine at http://www.plosmedicine.org/, Annals of Internal Medicine at http://www.annals.org/, and Epidemiology at http://www.epidem.com/). Information on the STROBE Initiative is available at http://www.strobe-statement.org.

For peer review only

For peer review only - http://bmjopen.bmj.com/site/about/guidelines.xhtml

BMJ Open

The association of C-reactive protein with future development of diabetes: A population-based 7-year cohort study among Norwegian adults aged 30 and older in the Tromsø Study 2007 – 2016

Journal:	*BMJ Open*
Manuscript ID	bmjopen-2022-070284.R2
Article Type:	Original research
Date Submitted by the Author:	21-Aug-2023
Complete List of Authors:	Tong, Kit I.; University Health Network<br>Hopstock, Laila ; UiT The Arctic University of Norway<br>Cook, Sarah; Imperial College London; London School of Hygiene & Tropical Medicine
**Primary Subject Heading**:	Diabetes and endocrinology
Secondary Subject Heading:	Epidemiology
Keywords:	Epidemiology < TROPICAL MEDICINE, DIABETES & ENDOCRINOLOGY, General diabetes < DIABETES & ENDOCRINOLOGY

SCHOLARONE™
Manuscripts

I, the Submitting Author has the right to grant and does grant on behalf of all authors of the Work (as defined in the below author licence), an exclusive licence and/or a non-exclusive licence for contributions from authors who are: i) UK Crown employees; ii) where BMJ has agreed a CC-BY licence shall apply, and/or iii) in accordance with the terms applicable for US Federal Government officers or employees acting as part of their official duties; on a worldwide, perpetual, irrevocable, royalty-free basis to BMJ Publishing Group Ltd ("BMJ") its licensees and where the relevant Journal is co-owned by BMJ to the co-owners of the Journal, to publish the Work in this journal and any other BMJ products and to exploit all rights, as set out in our licence.

The Submitting Author accepts and understands that any supply made under these terms is made by BMJ to the Submitting Author unless you are acting as an employee on behalf of your employer or a postgraduate student of an affiliated institution which is paying any applicable article publishing charge ("APC") for Open Access articles. Where the Submitting Author wishes to make the Work available on an Open Access basis (and intends to pay the relevant APC), the terms of reuse of such Open Access shall be governed by a Creative Commons licence – details of these licences and which Creative Commons licence will apply to this Work are set out in our licence referred to above.

Other than as permitted in any relevant BMJ Author's Self Archiving Policies, I confirm this Work has not been accepted for publication elsewhere, is not being considered for publication elsewhere and does not duplicate material already published. I confirm all authors consent to publication of this Work and authorise the granting of this licence.

For peer review only

The association of C-reactive protein with future development of diabetes: A population-based 7-year cohort study among Norwegian adults aged 30 and older in the Tromsø Study 2007 – 2016

Kit I. Tong,[1] Laila Arnesdatter Hopstock,[2] Sarah Cook,[3,4]

[1]Princess Margaret Cancer Centre, University Health Network, Toronto, Ontario, Canada. kit.tong@uhn.ca

[2]Department of Health and Care Sciences, UiT The Arctic University of Norway, Tromsø, Norway. laila.hopstock@uit.no

[3]Faculty of Epidemiology and Population Health, London School of Hygiene & Tropical Medicine, London, UK.

[4]School of Public Health, Imperial College London, London, UK. sarah.cook@imperial.ac.uk

Short running title: Association of hs-CRP with incident diabetes

Correspondence to:

Dr Sarah Cook; sarah.cook@imperial.ac.uk and Dr Kit I. Tong; kit.tong@uhn.ca

Word count: 5367

Number of tables: 3

Number of figures: 3

For peer review only

ABSTRACT

Objectives The extent to which observed associations between high-sensitivity C-reactive protein (hs-CRP) and incident diabetes are explained by obesity and hypertension remains unclear. This study aimed to investigate the association of hs-CRP with developing diabetes in a Norwegian general population sample.

Design A cohort study using two population-based surveys of the Tromsø Study: the sixth survey Tromsø6 (2007-2008) as baseline and the seventh survey Tromsø7 (2015-2016) at follow-up.

Setting Tromsø municipality of Norway, a country with increasing proportion of older adults and a high prevalence of overweight, obesity, and hypertension.

Participants 8,067 women and men without diabetes, aged 30 – 87 years, at baseline Tromsø6 who subsequently also participated in Tromsø7.

Outcome measures Diabetes defined by self-reported diabetes, diabetes medication use, and/or HbA1c ≥6.5% (≥ 48 mmol/mol) was modelled by logistic regression for the association with baseline hs-CRP, either stratified into 3-quantiles or as continuous variable, adjusted for demographic factors, behavioural and cardiovascular risk factors, lipid-lowering medication use, and hypertension. Interactions by sex, body mass index (BMI), hypertension, or abdominal obesity were assessed by adding interaction terms in the fully adjusted model.

Results There were 320 (4.0%) diabetes cases after 7 years. After multivariable adjustment including obesity and hypertension, individuals in the highest hs-CRP tertile 3 had 73% higher odds of developing diabetes (OR = 1.73; p-value, 0.004; 95% CI, 1.20 – 2.49) when compared with the lowest tertile or 28% higher odds of incidence per one-log of hs-CRP increment (OR = 1.28; p-value, 0.003; 95% CI 1.09 – 1.50). There was no evidence for interaction between hs-CRP and sex, hypertension, BMI, or abdominal obesity.

For peer review only - http://bmjopen.bmj.com/site/about/guidelines.xhtml

For peer review only

Conclusions Raised hs-CRP was associated with future diabetes development in a Norwegian adult population sample. The CRP-diabetes association could not be fully explained by obesity or hypertension.

For peer review only

For peer review only - http://bmjopen.bmj.com/site/about/guidelines.xhtml

STRENGTHS AND LIMITATIONS OF THIS STUDY

⇒ The 7-year population-based cohort study design allowed us to investigate the temporal association between hs-CRP and future diabetes development.

⇒ The study utilized a large overall cohort sample size with high quality, high participation rate and a low level (<11%) of missing data, which garners high internal validity.

⇒ A number of potential confounders were taken into account including medication use of lipid-lowering drugs, which were not included in other previous studies.

⇒ Definition of the baseline and outcome future diabetes were based partly on self-reported diagnosis and self-reported diabetes medication use although the diabetes case definition was additionally based on the more robust HbA1c laboratory-based blood test to improve both sensitivity and specificity.

⇒ The study focused on a sample of the Norwegian general population from one municipality in Northern Norway and hence the conclusions may not be generalizable beyond this region.

For peer review only

INTRODUCTION

Diabetes is a diverse metabolic disorder primarily characterized by impaired glucose tolerance and hyperglycaemia resulting from insulin deficiency or resistance. In 2019 there were approximately 463 million people with diabetes worldwide and the number of patients is constantly increasing.[1] The growing hyperglycaemia-related deaths, health care burden, and the associated socio-economic outcomes are pressing issues. Identification of modifiable risk factors and predictive biomarkers for diabetes becomes important to offer opportunities for early prevention and intervention.

Due to the conflicting broad definitions of type 1 and type 2 diabetes, there was a recent proposal for a β-cell-centric classification system of diabetes that encompasses interactions between β-cells function and abundance with genetics, insulin resistance, environmental factors, immune dysregulation, and inflammation, which underlie a wide spectrum of hyperglycaemic phenotypes found in diabetic patients.[2] Standardization of laboratory-based tests such as those that measure C-peptide, low-grade inflammation, β-cells mass, insulin resistance, and β-cells autoantibodies may facilitate better subtype diagnosis and subsequent precision care of diabetes.[2,3]

While disease progression of type 1 diabetes involves inflammation of the islet cells leading to ultimate massive β-cells loss and clinical diabetes,[4,5] systemic inflammation is considered as one of the mechanisms mediating the pathogenesis of type 2 diabetes.[4] Inflammation is a cellular defense system against infections and is an essential mechanism for normal tissue homeostasis.[6] Inflammatory cells produce cytokines leading to the production of acute phase proteins in the liver to promote tissue repair.[7] However, when timely resolution reinstating cellular homeostasis fails, the resulting chronic inflammation can also lead to tissue damage.[8] Given the emerging role of inflammation in the pathophysiology of diabetes and the associated metabolic disorders, there is

For peer review only

increasing interest in the development of anti-inflammatory therapeutics to improve both the prevention and the management of diabetes with numerous clinical trials showing various promising effects on type 1 and type 2 diabetes patients.[4,9,10] Several risk factors for diabetes - aging, obesity, smoking and physical inactivity - have been found to be positively associated with low-grade inflammation.[11–14] Excessive energy intake and sedentary lifestyle contribute to fat accumulation in tissues including muscles, liver, and pancreas, which may trigger chronic low-grade inflammation through adipokine-induced production of pro-inflammatory cytokines and chemokines, ultimately leading to insulin resistance and β-cell dysfunction.[10] Indeed, the elevation of acute-phase proteins such as C-reactive protein (CRP), a marker for systemic inflammation, has previously been shown to be associated with development of type 1 and type 2 diabetes.[15,16]

Demographic factors such as sex,[17,18] age,[11,19] and education;[20] lifestyle or behaviour risk factors for diabetes and/or CRP levels such as alcohol consumption,[21,22] daily smoking,[13,23] and physical inactivity;[14,24] clinical risk factors such as high body mass index (BMI),[12,25] abdominal obesity,[12,26,27] non-HDL cholesterol,[28,29] medication use of lipid lowering drug,[30,31] and the comorbidity hypertension are potential confounders that have been indicated associated with CRP levels and/or with diabetes incidences;[32] but not mediating the pathway between the association of CRP and diabetes incidences (figure 1).

There are conflicting conclusions in the literature on whether the association between high sensitivity (hs)-CRP and diabetes development is modified by sex and whether the association is truly independent of hypertension,[15,33–35] general obesity,[36–38] and abdominal obesity.[33,39] Evidences of interaction were also evident in some studies between CRP and

For peer review only - http://bmjopen.bmj.com/site/about/guidelines.xhtml

sex,[34,40] BMI,[15,38] hypertension,[15,41] or with waist circumference,[38] while other studies however did not observe evidence of interaction between hs-CRP with sex nor with BMI.[42,43] Lipid-lowering drugs such as statins, fibrates, nicotinic acid, and some other lipid modifying agents have both a lipid-lowering capacity and anti-inflammatory effect.[44] The use of these medications has been found able to lower hs-CRP levels;[30] while, conversely, the recipients of the drugs are inherently at risk of diabetes development due to indications for the medication use to reduce blood cholesterol and triglyceride levels.[45,46] Some, such as statins, are associated with elevated risk of new onset of diabetes while nicotinic acid is associated with impaired blood glucose control suggesting potential drug-induced side effects.[31] Some previous studies were not able to or did not adjust for the parameter of medication use leading to the questions of potential residual confounding.[15,33,36]

According to Norwegian Institute of Public Health, there was high prevalence of overweight and obesity combined in Norway.[47] Using a recent revised consensus definition of metabolism syndrome from the harmonized Adult Treatment Panel-III (ATP-III) criteria,[48] a recent repeated large cohort cross-sectional study of the rural Northern Norway has found that the prevalence of age-standardized metabolic syndrome (MetS) has progressed from 31.2% in 2003-2004 (n=6550) to 35.6% in 2012-2014 (n=6004) with concomitant increase in the mean MetS severity, which was believed to be driven by the marked increase in abdominal obesity among the study population over the years.[49]

Population-based studies assessing CRP as a marker for future diabetes incidences has not been reported for the Scandinavian countries. We aimed to investigate the association of hs-CRP on the development of diabetes after an interval of 7-years among adults in Norway, a Nordic country

For peer review only - http://bmjopen.bmj.com/site/about/guidelines.xhtml

with an increasing proportion of middle-aged and older adults and with a high prevalence of overweight, obesity, and hypertension.

For peer review only

For peer review only - http://bmjopen.bmj.com/site/about/guidelines.xhtml

METHODS

Setting

The Tromsø Study is a population-based health study in the Tromsø municipality, Norway.[50] Participants were invited from the whole birth cohorts and random sample of the Tromsø residents. Data collection include questionnaires, biological samples, and clinical examinations.

This study involved data from 2 surveys of the Tromsø Study: the sixth survey of the Tromsø Study (Tromsø6) 2007 – 2008 as baseline and the seventh survey of the Tromsø Study (Tromsø7) 2015 – 2016 as the follow-up time point after a 7-year interval.

In Tromsø6 2007 – 2008, all residents aged 40 – 42 and 60 – 87 years (n=12,578), a 10% random sample of residents aged 30 – 39 years (n=1,056), a 40% random sample of residents aged 43 – 59 years (n=5,787) and all previous participants who attended Tromsø4 second visit (if not already included in the three groups above) (n=341) were invited.[51] In total, 12,984 women and men attended Tromsø6 with 66% participation (figure 2)

In Tromsø7 2015 – 2016, all residents aged 40 years and older were invited (N=32,591).[52] In total, 21,083 women and men attended Tromsø7 with 65% participation (figure 2).

Study Design

A cohort study used two population-based surveys of the Tromsø Study: the sixth survey Tromsø6 (2007-2008) as baseline and the seventh survey Tromsø7 (2015-2016) at follow-up after 7-year interval. The association of the baseline hs-CRP, expressed as 3-quantiles, at Tromsø6 with future diabetes cases at Tromsø7 for the participants who did not have baseline diabetes at Tromsø6 was examined by logistic regression adjusted for a number of *a priori* potential confounding factors and then further tested for potential effect modifications by likelihood-ratio tests. The CRP-

For peer review only - http://bmjopen.bmj.com/site/about/guidelines.xhtml

diabetes association was further tested in the sensitivity analysis by repeating multivariable logistic regression using the baseline log hs-CRP, at Tromsø6, expressed as continuous independent variable.

Study Sample

Figure 2 depicts the flow chart for the selection of the study sample. In brief, participants without prevalent diabetes in Tromsø6 who had also attended Tromsø7 were included in this analysis.

In addition, individuals with baseline hs-CRP ≥10 mg/L, an indication of acute inflammatory condition at the time of examination,[53–57] would be excluded in the final analysis to mitigate the risk of measurement error. As acute inflammation may resolve over a short period of time and may not necessarily transit into chronic inflammatory condition.[58] A previous study involving 5,111 men aged 48 – 77 in the Oslo Study showed that individuals with health conditions of osteoporosis, asthma, chronic bronchitis/emphysema, diabetes, or myocardial infarction had a mean CRP level of 6.53 mg/L, 5.01 mg/L, 4.42 mg/L, 4.53 mg/L, and 4.27 mg/L, respectively.[59] These values were much less than hs-CRP of 10 mg/L.

In details, 12,984 participants attended Tromsø6. We excluded 5 participants who did not consent to medical research; 965 participants with baseline diabetes; 418 participants with baseline hs-CRP ≥10 mg/L; 144 participants without baseline hs-CRP data; and 1 participant without HbA1c nor self-reported diabetes diagnosis data with negative response to the use of insulin and blood sugar lowering drugs for determining baseline diabetes status. Among the 11,451 participants without baseline diabetes in Tromsø6 with hs-CRP <10 mg/L and valid measurements, a total of 8,071 also attended Tromsø7. Additionally, 4 participants were excluded due to inadequate information in Tromsø7 to determine diabetes status. Hence, 8,067 (3,704 men, 45.9%; 4,363

For peer review only - http://bmjopen.bmj.com/site/about/guidelines.xhtml

women, 54.1%) participants without baseline diabetes nor acute inflammation in Tromsø6 aged 30 – 87 years were included in the final analysis (figure 2).

Criteria defining diabetes condition is as described below in section "*Outcome Definition (Diabetes)*".

Data Collection Procedures

Data obtained by questionnaires, laboratory analyses, and clinical examinations have been described previously.[51,52,60] Briefly, blood samples without mandatory fasting were collected by EDTA anticoagulation tubes to measure HbA1c levels by high-performance liquid chromatography with Variant II (Bio-Rad Laboratories, Hercules, CA, USA) in Tromsø6 and by Tosoh G8 (Tosoh Bioscience, San Francisco, USA) in Tromsø7. Serum hs-CRP, total cholesterol, and high-density lipoprotein (HDL) cholesterol were measured with immunoturbidimetric and enzymatic colorimetric methods, respectively, by Modal PPE autoanalyzer (Roche Diagnostics Norway AS) in Tromsø6. Height in centimeters (cm) and weight in kilograms (kg) were measured by a Jenix DS 102 stadiometer (Dong Sahn Jenix, Seoul, Korea). Waist circumference was measured in cm by a Seca measuring tape at the level of the umbilicus. Three separate readings of systolic and diastolic blood pressure were taken, using a Dinamap Pro care 300 monitor (GE Healthcare, Norway), with 1-minute of intervals between each reading after a 2-minute seated rest. The mean systolic and diastolic blood pressure of readings 2 and 3 were used in this study.

Variables in the analyses

Exposure (hs-CRP) - The baseline hs-CRP at Tromsø6, stratified into 3 quantiles of ascending values, was used as laboratory-based proxy measurement for chronic inflammation to deduce its

For peer review only - http://bmjopen.bmj.com/site/about/guidelines.xhtml

impact on the odds of developing diabetes over 7 years in Tromsø7. Additionally, in the sensitivity analysis, hs-CRP was firstly log transformed due to its non-normally and skewed distribution. The log transformed hs-CRP was then used as a continuous independent variable to re-analyze the regression model.

Outcome Definition (Diabetes) - Known and unknown incident diabetes cases at Tromsø7 were identified by glycated hemoglobin (HbA1c) ≥ 6.5% (i.e., ≥ 48 mmol/mol),[60] and/or self-reported diabetes diagnosis ("Do you, or have you had diabetes?), and/or self-reported use of blood sugar lowering (ATC-code A10B) and/or insulin-containing medication (ATC-code A10A) for treating symptoms.[61] Self-reported previous diabetes only was not defined as diabetes.

Potential confounders - As described in the conceptual framework in figure 1 and in the introduction, previous studies suggested that the following variables are potential confounders that have been indicated associated with CRP levels and/or with diabetes incidences,[11–14,17–32] but not mediating the pathway between the association of CRP and diabetes incidence: They include demographic factors (age, sex, and education); behavioural risk factors (alcohol consumption frequency, daily smoking status, and physical activity); cardiovascular risk factors (body mass index (BMI), abdominal obesity, and non-high-density lipoprotein (non-HDL) cholesterol); use of lipid-lowering medications (ATC-code C10);[61] and comorbid hypertension.

Potential effect modifiers - Previous studies suggested that sex, BMI category, abdominal obesity, or hypertension may interact with hs-CRP although conflicting results have been found from other studies.[15,34,38,40–43]

Definition of the variables (confounders and/or effect modifiers):

For peer review only - http://bmjopen.bmj.com/site/about/guidelines.xhtml

- Age was divided into 5-year groups for the individuals aged 40 – 74 years. Due to data sparsity, the youngest and the eldest individuals were categorized into 10-year (30 – 39 years) and 12-year (75 – 87 years) age groups.
- Self-reported education, as a proxy for socioeconomic status, was defined by 5 levels (primary/secondary school, 1 – 2 years senior high school, high school diploma, < 4 years university, or ≥ 4 years university).
- Alcohol consumption frequency was defined by 5 levels (never drinker, monthly or less frequent drinker, 2 – 4 times per month, 2 – 3 times per week, or ≥ 4 times per week).
- Daily smoking was defined as current, previous or never.
- Physical activity was defined from self-report as sedentary (reading, watching TV, or other sedentary activity), low (walking, cycling, or other forms of exercise at least 4 hours a week), moderate (recreational sports, heavy gardening, etc. at least 4 hours a week), or vigorous (hard training or sports competitions, regularly several times a week).[51]
- BMI was categorized as < 25 kg/m^2, 25 – 29.9 kg/m^2, or ≥ 30 kg/m^2.[47]
- Abdominal obesity was categorized as abdominal obese when waist-to-height ratio ≥ 0.5. It was suggested by previous systematic review and meta-analysis studies that a waist-to-height ratio cutoff of 0.5 is suitable for adults of both sex and different ethnic backgrounds.[27,62]
- Non-HDL cholesterol was derived by negating HDL cholesterol from total serum cholesterol.
- Hypertension was defined by systolic blood pressure ≥ 140 mmHg and/or diastolic blood pressure ≥ 90 mmHg,[63] and/or self-reported use of blood pressure lowering drugs (ATC-code C02, antihypertensives; C03, diuretics; C07, beta blocking agents; C08, calcium channel blockers; C09, renin-angiotensin acting agents) for treatment.[61]

For peer review only - http://bmjopen.bmj.com/site/about/guidelines.xhtml

Statistical analysis

The hs-CRP levels of the study participants (table 1) were first stratified into three quantiles, 0.14 – 0.77 mg/L (tertile 1), 0.78 – 1.69 mg/L (tertile 2), and 1.70 – 9.98 mg/L (tertile 3) to conduct most of the analyses described below unless otherwise specified.

Age-standardized cumulative diabetes incidence - In figure 3, age-standardized cumulative diabetes incidence across baseline hs-CRP tertiles, overall or sex-specific, was calculated using direct standardization to the 2013 European Standard Population to examine any dosage effect of hs-CRP exposure on outcome diabetes cases.[64]

Baseline characteristics - In table 1, the descriptive statistics of the baseline characteristics for the sample population across the 3-quantiles of baseline hs-CRP was examined:

By Pearson's chi-squared test for categorical variables (sex, age, BMI category, hypertension, blood pressure lowering drugs, abdominal obesity, lipid-lowering drugs, alcohol consumption frequency, daily smoking, physical activity, education).

By Kruskal-Wallis test for continuous variables with non-normal distribution (hs-CRP).

By one-way ANOVA for continuous variables with normal distribution (waist-to-height ratio, non-HDL cholesterol, systolic blood pressure, diastolic blood pressure).

Logistic regression models - In table 2, crude model was built to examine the association of baseline hs-CRP tertiles with diabetes incidence in Tromsø7. The model was then multivariable adjusted for a set of a *priori* determined covariates (sex, age, BMI, hypertension, abdominal obesity, non-HDL cholesterol, lipid-lowering drug use, alcohol consumption frequency, daily

For peer review only

For peer review only - http://bmjopen.bmj.com/site/about/guidelines.xhtml

	Tertiles of hs-CRP (mg/L)				
	Tertile 1 (0.14 – 0.77)	Tertile 2 (0.78 – 1.69)	Tertile 3 (1.70 – 9.98)	p-value*	Total
**N**	2,716	2,673	2,678		8,067
**Sex**, n (%)					
Male	1,139 (41.9)	1,336 (50.0)	1,229 (45.9)	<0.001	3,704 (45.9)
Female	1,577 (58.1)	1,337 (50.0)	1,449 (54.1)		4,363 (54.1)
**Age**, n (col, %)					
30 – 39 years	108 (4.0)	94 (3.5)	78 (2.9)	<0.001	280 (3.5)
40 – 44 years	728 (26.8)	485 (18.1)	435 (16.2)		1,648 (20.4)
45 – 49 years	332 (12.2)	315 (11.8)	267 (10.0)		914 (11.3)
50 – 54 years	325 (12.0)	285 (10.7)	274 (10.2)		884 (11.0)
55 – 59 years	268 (9.9)	309 (11.6)	301 (11.2)		878 (10.9)
60 – 64 years	471 (17.3)	551 (20.6)	598 (22.3)		1,620 (20.1)
65 – 69 years	283 (10.4)	349 (13.1)	367 (13.7)		999 (12.4)
70 – 74 years	138 (5.1)	184 (6.9)	210 (7.8)		532 (6.6)
75 – 87 years	63 (2.3)	101 (3.8)	148 (5.5)		312 (3.9)
**hs-CRP**, Median, mg/L	0.50	1.12	2.83	0.0001	1.12
(25th, 75th percentiles)	(0.38, 0.62)	(0.93, 1.39)	(2.13, 4.27)		(0.62, 2.13)
**BMI category**, n (col, %)					
< 25 kg/m^2	1,507 (55.5)	865 (32.4)	564 (21.1)	<0.001	2,936 (36.4)
25 – 29.9 kg/m^2	1,048 (38.6)	1,362 (51.0)	1,270 (47.4)		3,680 (45.6)
≥ 30 kg/m^2	160 (5.9)	445 (16.7)	843 (31.5)		1,448 (18.0)
Missing	1	1	1		3
**Hypertension**, n (col, %)	845 (31.2)	1,199 (45.0)	1,458 (54.5)	<0.001	3,502 (43.5)
Missing	5	6	4		15
**Blood pressure**, mean ± SD					
Systolic, mmHg	127.65 ± 20.35	134.22 ± 22.11	137.52 ± 21.54	<0.0001	133.10 ± 21.73
Diastolic, mmHg	75.49 ± 10.12	78.10 ± 10.39	79.20 ± 10.65	<0.0001	77.59 ± 10.50
**Blood pressure lowering drugs**					
In use, n (col, %)	355 (13.1)	528 (19.8)	717 (26.8)	<0.001	1,600 (19.8)
**Waist-to-height ratio**					
Mean ± SD	0.52 ± 0.06	0.55 ± 0.06	0.58 ± 0.07	<0.0001	0.55 ± 0.07
**Abdominal obesity**, n (col, %)	1,646 (62.8)	2,125 (81.7)	2,341 (90.1)	<0.001	6,112 (78.2)
Missing	96	73	80		249
**Non-HDL cholesterol**, mmol/L					
Mean ± SD	3.85 ± 1.02	4.20 ± 1.06	4.32 ± 1.08	<0.0001	4.12 ± 1.07
**Lipid-lowering drugs**					
In use, n (col, %)	260 (9.6)	296 (11.1)	315 (11.8)	0.030	871 (10.8)
**Alcohol consumption frequency**, n (col, %)					
Never drinker	172 (6.4)	218 (8.2)	242 (9.1)	<0.001	632 (7.9)
Monthly or less frequent	617 (22.9)	701 (26.5)	799 (30.1)		2,117 (26.5)
2 – 4 times a month	1,146 (42.5)	1,088 (41.1)	1,054 (39.7)		3,288 (41.1)
2 – 3 times a week	596 (22.1)	501 (18.9)	424 (16.0)		1,521 (19.0)
≥ 4 times a week	167 (6.2)	141 (5.3)	133 (5.0)		441 (5.5)
Missing	18	24	26		68
**Daily smoking**, n (col, %)					
Current	387 (14.4)	469 (17.7)	576 (21.8)	<0.001	1,432 (17.9)
Previously	1,147 (42.6)	1,115 (42.1)	1,171 (44.3)		3,433 (43.0)
Never	1,156 (43.0)	1,066 (40.2)	899 (34.0)		3,121 (39.1)
Missing	26	23	32		81
**Physical activity**, n (col, %)					
Sedentary	352 (13.6)	383 (15.3)	536 (21.6)	<0.001	1,271 (16.8)
Low	1,580 (60.8)	1,532 (61.0)	1,542 (62.2)		4,654 (61.3)
Moderate	589 (22.7)	552 (22.0)	378 (15.2)		1,519 (20.0)
Vigorous	76 (2.9)	44 (1.8)	25 (1.0)		145 (1.9)
Missing	119	162	197		478

For peer review only - http://bmjopen.bmj.com/site/about/guidelines.xhtml

**Education**, n (col, %)					
Primary/Secondary	516 (19.2)	641 (24.2)	762 (28.8)	<0.001	1,919 (24.0)
1-2 years senior high school	618 (22.9)	722 (27.3)	746 (28.2)		2,086 (26.1)
High School diploma	220 (8.2)	206 (7.8)	212 (8.0)		638 (8.0)
College/University < 4 years	542 (20.1)	504 (19.0)	479 (18.1)		1,525 (19.1)
College/University ≥ 4 years	799 (29.7)	575 (21.7)	450 (17.0)		1,824 (22.8)
Missing	21	25	29		75

Table 1 – Baseline characteristics of the study participants overall and across hs-CRP tertiles in the Tromsø Study 2007 – 2008.

* p-value across hs-CRP tertiles was obtained by Pearson's chi-squared test for categorial variables, by Kruskal-Wallis test for continuous variables with non-normal distribution, or by one-way ANOVA for continuous variables with normal distribution.

For peer review only

	Tertiles of hs-CRP (mg/L)				
	**Tertile 1** (0.14 – 0.77)	**Tertile 2** (0.78 – 1.69)	**Tertile 3** (1.70 – 9.98)	**p-value**	**Total**
**N**	2,716	2,673	2,678		8,067
Diabetes incidence *, n (%)	52 (1.9)	97 (3.6)	171 (6.4)	<0.001†	320 (4.0)
**Logistic regression, hs-CRP in tertiles, OR (p-value, 95% CI)**					
Crude model	1.00 (ref)	1.93 (<0.001, 1.37 – 2.71)	3.49 (<0.001, 2.55 – 4.79)		
Multivariable adjusted ‡	1.00 (ref)	1.21 (0.326, 0.83 – 1.78)	1.73 (0.004, 1.20 – 2.49)		
**Multivariable adjusted model + interaction term††**			**p-value, LRT §**		
+ hs-CRP tertile X Sex			0.902		
+ hs-CRP tertile X BMI			0.807		
+ hs-CRP tertile X hypertension			0.773		
+ hs-CRP tertile X abdominal obesity			0.632		
**Logistic regression, hs-CRP as continuous variable, OR (p-value, 95% CI) by 1 log increment of hs-CRP**					
Crude model			1.75 (<0.001, 1.53 – 1.99)		
Multivariable adjusted ‡			1.28 (0.003, 1.09 – 1.50)		

Table 2 – Crude and multivariable adjusted logistic regression models for the association of hs-CRP and diabetes incidences in the Tromsø Study 2007 – 2016.

* Known and unknown diabetes incidence in Tromsø7. † p-value across hs-CRP tertiles by Pearson's chi-squared test. ‡ Adjusted for sex, age, body mass index, abdominal obesity, non-HDL cholesterol, lipid lowering drug use, hypertension, alcohol consumption frequency, daily smoking status, physical activity, and education. †† Models with hs-CRP in teriles adjusted for multivariables as described in ‡, except for variables included as interaction terms. § LRT, Likelihood-ratio test for testing interaction. OR, odds ratio; ref, reference.

For peer review only

For peer review only - http://bmjopen.bmj.com/site/about/guidelines.xhtml

smoking status, physical activity, and education) to correct for any potential confounding effects on the CRP-diabetes association.

Evaluation of potential effect modification - There were conflicting conclusions in previous studies on whether CRP-diabetes association is effect modified by sex, BMI, abdominal obesity, or hypertension.[15,34,38,40–43] In table 2, we examined the potential effect modifiers by adding interaction term for sex (hs-CRP tertile x sex), anthropometric factors (hs-CRP tertile x BMI or hs-CRP tertile x abdominal obesity), or hypertension (hs-CRP tertile x hypertension) separately to the final multivariable logistic regression model and tested the model by likelihood ratio tests (LRT). The P-value of the LRT was used to evaluate whether the addition of any interaction term constitutes a better model. The null hypothesis is that the model is better fit without the addition of interaction term.

Sensitivity analyses - In table 2, the analysis was repeated by using baseline hs-CRP as a continuous independent variable in conducting the logistic regression. Due to its skewed distribution, the baseline hs-CRP in mg/L was firstly log transformed before assessing again its association with diabetes incidence at Tromsø7. Logistic regression, with or without multivariable adjusted for *a priori* covariates as described above, was used to evaluate the odds of diabetes cases by one-log increment of baseline hs-CRP among all study sample.

As discussed in the Methods session, individuals with baseline hs-CRP ≥10 mg/L might be potentially experiencing acute inflammatory conditions at the time of the lab-based examination during the Tromsø Study survey.[53–58] To assess the robustness of the analysis, the logistic regression models with or without multivariable adjusted for *a priori* covariates as described above were repeated by including study participants with hs-CRP ≥10 mg/L using baseline hs-CRP as 3-quantiles and also as continuous independent variables (table 3).

For peer review only - http://bmjopen.bmj.com/site/about/guidelines.xhtml

	Tertiles of hs-CRP (mg/L)			p-value	Total
	**Tertile 1** (0.14 – 0.79)	**Tertile 2** (0.80 – 1.78)	**Tertile 3** (1.79 – 136.60)		
**N**	2,801	2,748	2,754		8,303
Diabetes incidence *, n (%)	56 (2.0)	101 (3.7)	187 (6.8)	<0.001†	344 (4.1)
**Logistic regression, hs-CRP in tertiles, OR (p-value, 95% CI)**					
Crude model	1.00 (ref)	1.87 (<0.001, 1.34 – 2.60)	3.57 (<0.001, 2.64 – 4.84)		
Multivariable adjusted ‡	1.00 (ref)	1.13 (0.525, 0.78 – 1.64)	1.68 (0.004, 1.18 – 2.40)		
**Logistic regression, hs-CRP as continuous variable, OR (p-value, 95% CI) by 1 log increment of hs-CRP**					
Crude model		1.63 (<0.001, 1.47 – 1.81)			
Multivariable adjusted ‡		1.28 (<0.001, 1.12 – 1.46)			

Table 3 – Sensitivity analysis including study participants with hs-CRP ≥ 10mg/L in the Tromsø Study 2007 – 2016.

* Known and unknown diabetes incidence in Tromsø7. † p-value across hs-CRP tertiles by Pearson's chi-squared test. ‡ Adjusted for sex, age, body mass index, abdominal obesity, non-HDL cholesterol, lipid lowering drug use, hypertension, alcohol consumption frequency, daily smoking status, physical activity, and education. OR, odds ratio; ref, reference.

For peer review only

All analysis was performed using STATA v.14.2 (StataCorp. 2015. Stata Statistical Software: Release 14. College Station, TX: StataCorp LP).

Patient and public involvement

There was no patient involvement in the study. The original cohort data of the two surveys were collected with ethic approvals as described under Ethics Approval section in this paper. Only anonymous data from participants who had consented for medical research in the original Tromsø Study were used in this study. The participants from the original Tromsø Study did not participate in the development of the research question of this study nor the outcome measures of this study informed by patients' priorities, experience, and preferences.

For peer review only

For peer review only - http://bmjopen.bmj.com/site/about/guidelines.xhtml

RESULTS

Baseline characteristics

As depicted in table 1, the total study sample of 8,067 individuals had a median hs-CRP level of 1.12 mg/L. The median hs-CRP levels were 0.5 mg/L, 1.12 mg/L, and 2.83 mg/L in tertile 1, 2, 3 respectively. Similarly, a previous study employing a large Norwegian community-based surveys, the Nord-Trøndelag Heath Study (HUNT), reported a median hs-CRP level of 1.1 mg/L among the entire study sample of 30,669 aged from 20 and older.[65]

The baseline characteristics (waist-to-height ratio, abdominal obesity, non-HDL cholesterol, systolic/diastolic blood pressure, hypertension, blood pressure lowering drugs, daily smoking, alcohol consumption frequency, BMI, physical activity, and education) of the participants exhibited statistical evidence for a difference across the 3 tertiles with increasing values of hs-CRP (p-values <0.001 – <0.0001) (table 1). Conversely, lipid-lowering drug usage only showed modest impact (p-value = 0.03). In tertiles 2 and 3, 55% and 60.5% of the participants were aged ≥ 55 years, respectively, while 82% and 90% of these participants were categorized as abdominal obesity. In addition, participants in tertiles 2 and 3 had higher percentage of overweight or obesity, hypertension, physically inactive, daily smokers, lower education, and never or infrequent drinking when compared with those in tertile 1.

Age-standardized cumulative diabetes incidence

There was a more than 3-fold increase of overall age-standardized cumulative diabetes incidence in the highest hs-CRP tertile 3 (6.3%) when compared with the reference tertile 1 (2.0%). This trend was apparent in both women (1.5% vs 5.8%, 3.9-fold increase) and men (2.8% vs 7.2%, 2.6-fold increase) (figure 3).

Baseline hs-CRP associates with future diabetes development

For peer review only - http://bmjopen.bmj.com/site/about/guidelines.xhtml

There were 320 (4.0%) known and unknown incident cases of diabetes during follow-up (table 2). The odds of diabetes incidence were almost 2 times (crude OR=1.93; p-value <0.001; 95% CI 1.37 – 2.71) and 3.5 times (crude OR=3.49; p-value <0.001; 95% CI 2.55 – 4.79) higher among participants of hs-CRP tertile 2 and tertile 3, respectively. Although the strength of the association reduced in the fully adjusted model, positive association remained and the participants in hs-CRP tertile 3 had 73% higher odds of future diabetes (OR = 1.73; p-value, 0.004; 95% CI, 1.20 – 2.49). The addition of the individual interaction term of hs-CRP tertile x sex (Likelihood-ratio tests (LRT) p-value, 0.902), hs-CRP tertile x BMI (LRT p-value, 0.807), hs-CRP tertile x hypertension (LRT p-value, 0.773), or hs-CRP tertile x abdominal obesity (LRT p-value, 0.632) to the multivariable adjusted model did not support any compound effects of these covariates (table 2).

Sensitivity analyses

In the sensitivity analysis depicted in table 2, consistent with the analyses using hs-CRP tertiles, a positive association between log transformed hs-CRP and diabetes development was observed. For every one-log baseline hs-CRP increment, there was 75% increase in odds of diabetes cases (crude OR = 1.75; p-value, <0.001; 95% CI 1.53 – 1.99). Positive association maintained after multivariable adjustment with 28% higher odds of diabetes development (OR = 1.28; p-value, 0.003; 95% CI 1.09 – 1.50) per one-log increase of baseline hs-CRP.

Another sensitivity analysis including study participants with hs-CRP ≥10 mg/L was as described in table 3. There were additional 236 study participants with valid measurements included in this sensitivity analysis. With the exception of the upper limit of hs-CRP level in hs-CRP tertile 3, there was less than 0.1 mg/L in the changes of hs-CRP measurement among the three hs-CRP quantiles. The upper limit of hs-CRP tertile 3 had drastically increased more than 13-fold to 136.60 mg/L, broadening particularly the range of hs-CRP measurement in tertile 3. There were 344 new known

For peer review only - http://bmjopen.bmj.com/site/about/guidelines.xhtml

For peer review only

and unknown diabetes cases, accounting for 4.1% of these 8,303 study samples, which is approximately 0.1% increase when comparing with the study samples excluding participants with hs-CRP ≥10 mg/L in table 2. Even after including study participants with hs-CRP ≥10mg/L, consistent positive association was observed with 68% increase in multivariable adjusted odds (OR = 1.68; p-value, 0.004; 95% CI, 1.18 – 2.40) of diabetes cases among the participants in hs-CRP tertile 3 (table 3). In general, by including participants with potentially acute inflammation (hs-CRP ≥10mg/L) conditions, the crude and adjusted associations were slightly biased towards null (table 2 versus table 3). Nevertheless, the models using hs-CRP as 3-quantiles remained robust in both scenarios. Similarly, when the sensitivity analysis was repeated among these 8,303 study samples using hs-CRP as continuous independent variable, the crude OR also slightly biased towards null (table 2 versus table 3), denoting a 63% increase in crude odds of new diabetes cases (crude OR = 1.63; p-value, <0.001; 95% CI, 1.47 – 1.81) by 1 log increment of hs-CRP. Nevertheless, the multivariable adjusted association (OR = 1.28; p-value, <0.001; 95% CI 1.12 – 1.46; table 3) per one-log increase of baseline hs-CRP remained robust even after including participants with potentially acute inflammation (hs-CRP ≥10mg/L) conditions.

For peer review only - http://bmjopen.bmj.com/site/about/guidelines.xhtml

DISCUSSION

We found a marked association between high hs-CRP and future diabetes incidence at 7-year follow-up in this study of adults from a general Norwegian population.

Comparison to other studies

A prospective study conducted in Spain demonstrated a positive association between elevated hs-CRP with future diabetes development after adjusting for age, sex, and obesity but a study in UK found that the association was attenuated and no longer significant after adjusting for BMI and waist-to-hip ratio.[36,39] While some others showed that the association became insignificant in men but maintained in women after adjusting for BMI, suggesting evidence of interaction between hs-CRP and sex on the association.[34,40] However, the present study did not observe evidence for interaction between hs-CRP and sex, BMI, abdominal obesity, or hypertension. The association was also maintained after adjusting for multiple factors including BMI and abdominal obesity. Whereas some other studies did not exclude individuals with elevated baseline CRP levels above 10 mg/L,[34,36] which might have uplifted the exposure level due to the potential underlying issues of acute inflammatory condition other than the risk associated with diabetes development. This heightened exposure threshold likely attenuated the true association of the exposure and outcome by means of non-differential misclassification. Indeed, a slight bias towards null was observed in our sensitivity analyses (table 2 versus table 3) after including study participants with hs-CRP ≥10mg/L, especially when stratifying participants into three hs-CRP quantiles. Nevertheless, the positive association remained robust in both scenarios.

A Finnish cohort study relied primarily on the diagnosed diabetes data through medical registries and self-reported surveys,[40] whereas a German case-control study identified baseline diabetes solely by self-reported information.[34] There might be limitation of misclassifying baseline

For peer review only

For peer review only - http://bmjopen.bmj.com/site/about/guidelines.xhtml

diabetes due to bias on self-reported diabetes. The lack of validated laboratory-based tests such as HbA1c levels or fasting-blood sugar content in these studies might fail to capture any unknown diabetes at baseline, which might have led to non-differential misclassification and could have diluted the strength of the association. Whereas, in the German study, the differential misclassification of the baseline unknown diabetes among the controls might have biased the association leaning towards null due to the case-control study design,[34] which in part might be explaining the attenuation in the strength of association after adjusting for BMI. Lee and colleagues,[39] on the other hand, used a HbA1c level cutoff of >7.0% (i.e., >53 mmol/mol) for identifying unknown diabetes status at baseline and during follow-up. While this higher cutoff had been used for defining diabetes status, this deviation from the cutoff of 6.5% (i.e., 48 mmol/mol) recommended by the World Health Organization (WHO) could introduce inconsistencies with the findings of other studies which chose to follow the WHO recommended standards.[66]

There is increasing awareness that elevated hs-CRP levels and diabetes incidences are associated with adiposity.[26] The association of elevated hs-CRP with central adiposity could be independent of BMI.[67] Some European studies adjusted their models for BMI but not for central adiposity.[34,36,40] Waist-to-hip ratio has been indicated using various sex-specific thresholds.[68] Although Lee and colleagues had adjusted for both BMI and waist-to-hip ratio,[39] the inherent limitation of the waist-to-hip ratio might have led to residual confounding in some of these published analyses. Indeed, Lee and colleagues suggested that the differential results found in a meta-analysis of 16 published studies might be due to, in part, the adjustment for different indexes of central adiposity.[39] The waist-to-height ratio had been found to be sex-, age-, and ethnicity-independent;[68] and the use of this index in the current study might have addressed more adequately the confounding impact of the fat storage profile for both men and women.

For peer review only - http://bmjopen.bmj.com/site/about/guidelines.xhtml

Aging-associated chronic inflammation has been proposed as playing a part in the etiology of type 2 diabetes.[69] Although aging has long been identified as a risk factor and positively correlates with pathogenesis, it would be challenging to pinpoint a particular age cutoff to inform clinical practice. Previous research have suggested multiple sources of aging-associated chronic inflammation including age-related accumulation of damaged cells, changes in gut microbiota over age, increasing cellular senescence and the subsequent secretion of proinflammatory cytokines namely the senescence-associated secretory phenotype or SASP, and age-associated modification of the immune system among others.[69] Although fat is a good source of inflammatory cytokines, obesity alone may not represent the entire spectrum of mechanisms underlying the development of diabetes via chronic systemic inflammation.

Individuals deemed prediabetes, defined by HbA1c between 5.7 – 6.4% or 39 – 47 mmol/ml, were included in this current study. Prediabetes state has been considered as risk for diabetes transformation.[70] Although positive association had been previously implicated,[71,72] the temporal relationship between elevated hs-CRP and prediabetes condition remained unclear. Glycemic parameters such as HbA1c could provide estimates to reflect the average blood glucose concentration of an individual over the preceding 2 to 3 months. Nevertheless, the method also elicits certain extent of inaccuracies due to some glucose-independent variation, although corrective modeling has recently been proposed.[73]

Strengths of the study

This study has several strengths. The 7-year follow-up study allowed us to investigate the temporal association of hs-CRP on diabetes development. The study population was recruited and sampled from all residents of the Tromsø municipality with validated systematic data collection for each of the repetitive surveys, including the questionnaires, the clinical examinations and the lab

For peer review only - http://bmjopen.bmj.com/site/about/guidelines.xhtml

assessments. The data being analyzed were of high quality with high participation in the study, and low levels of missing data in general (<11%) – which garners high internal validity. No findings from similar population-based studies conducted in the Scandinavian countries investigating the association between CRP and incident diabetes have been previously reported. The current findings of the positive CRP-diabetes association may inform public health strategies for prevention and surveillance of diabetes in the Nordic region where lifestyle, culture, demography, health care and socioeconomic factors resemble Norway.

The data available in the current analysis included the information of the use of lipid-lowering drugs, which are used in primary and secondary prevention of cardiovascular diseases but have previously been shown capable in reducing hs-CRP levels but at the same time might contribute to drug-induced side effects of impaired glycemic control or new onset of diabetes.[31] These features might have led to residual confounding in some other previous studies which were not able to or did not include medication use in their multivariable adjusted models.[15,33] Moreover, the current study had utilized a large overall cohort sample size.

Limitations of the study

Limitations of this study include the lack of information of diabetes subtype. Since the study sample only included adults 30 years of age and above, primarily newly developed type 2 diabetes were expected. The study focused on a Norwegian general population and hence the conclusion may not be generalizable beyond this region. Since the baseline and future developed diabetes cases were self-reported, there might be potential non-differential misclassification of the outcome due to reporting bias. Similarly, self-reported medication for treating symptoms did not come with physician, medical record, or medical registry confirmation might have led to potential measurement error in the outcome. Nevertheless, previous studies have found that self-reported

For peer review only - http://bmjopen.bmj.com/site/about/guidelines.xhtml

diabetes often accounted for high specificity (84.5% to 99.7%) although it can exhibit low sensitivity (41.5% to 80.4%).[74–76] Previous studies had reported the impact from socioeconomic position and demographic characteristics such that the sensitivity of self-reported diabetes can be elevated with higher education levels and among urban dwellers.[76] In the current study, validated laboratory-based measurement of the HbA1c level was utilized in addition to self-reported diabetes and diabetes medication use for treating symptoms to enhance both the sensitivity and specificity when capturing the known and unknown true positive and false negative diabetes cases.

We did not include information on diet, which is another potential confounder. Diet can be negatively or positively associated with hs-CRP level and with future diabetes development depending on the diet types and patterns.[77] It would be of interest to include this parameter in future studies. In addition, diet is also linked with socioeconomic status which was apparently correlated with hs-CRP level as depicted in this report. On the other hand, due to data sparsity, we had combined participants with underweight condition (< 18.5 kg/m^2) with those with normal BMI 18.5 - 24.9 kg/m^2) as a single entity BMI (< 25 kg/m^2) in the model. Given that the overweight and obesity conditions were more likely associated with hs-CRP and/or diabetes development,[12,25–27] the grouping of the small number of underweight with the normal BMI participants should not introduce impactful confounding effects in the model.[78]

As chronic inflammation relates to the etiology of type 1 diabetes, it would be of interest to study its association with hs-CRP by utilizing a more defined type 1 diabetes cohort. It is also of interest to include hormone replacement therapy as a potential confounder since this medication for treating symptoms had been implicated associated with elevated hs-CRP levels but also implicated in the reduction of abdominal fat.[79,80]

For peer review only - http://bmjopen.bmj.com/site/about/guidelines.xhtml

CONCLUSION

In conclusion, this study has demonstrated that hs-CRP is associated with future diabetes development in a population-based sample of Norwegian adults. This association was consistent across sex, body mass index, abdominal obesity and hypertension. In combination with glycemic parameters such as HbA1c or fasting plasma glucose, this stable blood-based biomarker can potentially be used to facilitate risk group stratification, which may provide opportunities for early prevention or intervention of diabetes through clinical practices, public health education, and anti-inflammatory therapeutics development. Previous studies had depicted ethnic variations of CRP levels which we were not able to explore within this study.[80,81] When comparing with white, after multivariable adjustment including BMI, black individuals had higher serum CRP concentrations while Asians including Chinese and Japanese had lower levels. Future studies to determine the potentially ethnicity-dependent cutoffs of hs-CRP for risk stratification are warranted.

For peer review only - http://bmjopen.bmj.com/site/about/guidelines.xhtml

ACKNOWLEGMENTS

We would like to thank the participants of the Tromsø Study. We would like to extend our gratitude to Dr. Anne Elise Eggen, Dr. Ola Løvsletten, Ms. Julie-Helene D. Sørensen, and the members of the Tromsø Study Data and Publication Committee of UiT The Arctic University of Norway to make the Tromsø dataset available for this study.

For peer review only

For peer review only - http://bmjopen.bmj.com/site/about/guidelines.xhtml

CONTRIBUTORS

K.I.T. conceived the research question of the present study based on the suggestion from S.C. on a project relating to diabetes and the Tromsø Study. K.I.T. planned out the study design, analyzed and interpreted the data, constructed figures and tables, wrote and revised the manuscript. L.A.H. and S.C. contributed to the discussion of the research question, study design, analysis, and data interpretation. L.A.H. and S.C. took part in the critical revision of the manuscript and provided overall supervision of the study. S.C. coordinated the collaboration of the study. L.A.H took part in the data collection of Tromsø6 and Tromsø7. L.A.H. took part in the planning and management of Tromsø7. K.I.T. is the guarantor of this work and, as such, had full access to all the data in the study and takes responsibility for the integrity of the data and the accuracy of the data analysis. All authors approved the final manuscript.

For peer review only

For peer review only - http://bmjopen.bmj.com/site/about/guidelines.xhtml

COMPLETING INTERESTS

Authors declare no potential conflicts of interest relevant to this article.

For peer review only

ETHICS APPROVAL

Tromsø6 was approved by the Data Inspectorate of Norway and the Regional Committee for Medical and Health Research Ethics Northern Norway (REC North) (Ref. 121/2006) and Tromsø7 by the Norwegian Data Protection Authority (Ref. 14/01463-4/CGN) and REC North (Ref. 2014/940). The current study was approved by the London School of Hygiene & Tropical Medicine Ethics Committee (Ref. 2566).

For peer review only

DATA AVAILABILITY STATEMENT

Data are not publicly available but may be obtained from UiT The Arctic University of Norway through application and project approval by the Data and Publication Committee. The data used in this study are owned and managed by UiT The Arctic University of Norway (https://uit.no/research/tromsostudy).

For peer review only

FUNDING STATEMENT

Funding for the Tromsø Study was obtained from UiT The Arctic University of Norway, Northern Norway Regional Health Authority, Ministry of Health and Care Services, Norwegian Research Council, and various public and charity research funds in Norway. For SC infrastructure support for this research was provided by the NIHR Imperial Biomedical Research Centre. There was no external funding for this specific paper. The funding bodies have no role in the design of the study, data collection, analysis, interpretation of data, or in writing the manuscript.

For peer review only

REFERENCES

1 Saeedi P, Petersohn I, Salpea P, et al. Global and regional diabetes prevalence estimates for 2019 and projections for 2030 and 2045: Results from the International Diabetes Federation Diabetes Atlas, 9th edition. *Diabetes Research and Clinical Practice* 2019;157:107843.
2 Schwartz SS, Epstein S, Corkey BE, *et al*. The Time Is Right for a New Classification System for Diabetes: Rationale and Implications of the β-Cell-Centric Classification Schema. *Diabetes Care* 2016;39:179–86.
3 Classification of diabetes mellitus. Geneva: World Health Organization. 2019. Available: https://www.who.int/publications/i/item/classification-of-diabetes-mellitus [Accessed 11 Sept 2021].
4 Shoelson SE, Lee J, Goldfine AB. Inflammation and insulin resistance. *J Clin Invest* 2006;116:1793–801.
5 Clark M, Kroger CJ, Tisch RM. Type 1 Diabetes: A chronic Anti-self-inflammatory response. *Front Immunol* 2017;8:1898.
6 Medzhitov R. Origin and physiological roles of inflammation. *Nature* 2008;454:428–35.
7 Castell JV, Gómez-Lechón MJ, David M, *et al*. Acute-phase response of human hepatocytes: regulation of acute-phase protein synthesis by interleukin-6. *Hepatology* 1990;12:1179–86.
8 Chen L, Deng H, Cui H, *et al*. Inflammatory responses and inflammation-associated diseases in organs. *Oncotarget* 2018;9:7204–18.
9 Donath MY. Targeting inflammation in the treatment of type 2 diabetes: time to start. *Nat Rev Drug Discov* 2014;13(6):465-76.
10 Tsalamandris S, Antonopoulos AS, Oikonomou E, *et al*. The Role of Inflammation in Diabetes: Current Concepts and Future Perspectives. *Eur Cardiol* 2019;14:50–9.
11 Guarner V, Rubio-Ruiz ME. Low-grade systemic inflammation connects aging, metabolic syndrome and cardiovascular disease. *Interdiscip Top Gerontol* 2015;40:99–106.
12 Visser M, Bouter LM, McQuillan GM, *et al*. Elevated C-reactive protein levels in overweight and obese adults. *JAMA* 1999;282:2131–5.
13 Fröhlich M, Sund M, Löwel H, *et al*. Independent association of various smoking characteristics with markers of systemic inflammation in men. Results from a representative sample of the general population (MONICA Augsburg Survey 1994/95). *Eur Heart J* 2003;24:1365–72.
14 Yu Z, Ye X, Wang J, *et al*. Associations of physical activity with inflammatory factors, adipocytokines, and metabolic syndrome in middle-aged and older chinese people. *Circulation* 2009;119:2969–77.
15 Doi Y, Kiyohara Y, Kubo M, *et al*. Elevated C-reactive protein is a predictor of the development of diabetes in a general Japanese population: the Hisayama Study. *Diabetes Care* 2005;28:2497–500.
16 Chase HP, Cooper S, Osberg I, *et al*. Elevated C-reactive protein levels in the development of type 1 diabetes. *Diabetes* 2004;53:2569–73.
17 Khera A, McGuire DK, Murphy SA, *et al*. Race and gender differences in C-reactive protein levels. *J Am Coll Cardiol* 2005;46:464–9.
18 Kautzky-Willer A, Harreiter J, Pacini G. Sex and Gender Differences in Risk, Pathophysiology and Complications of Type 2 Diabetes Mellitus. *Endocr Rev* 2016;37:278–316.

For peer review only - http://bmjopen.bmj.com/site/about/guidelines.xhtml

19 Kirkman MS, Briscoe VJ, Clark N, *et al*. Diabetes in older adults. *Diabetes Care* 2012;35:2650–64.
20 Friedman EM, Herd P. Income, education, and inflammation: differential associations in a national probability sample (The MIDUS study). *Psychosom Med* 2010;72:290–300.
21 Imhof A, Froehlich M, Brenner H, *et al*. Effect of alcohol consumption on systemic markers of inflammation. *Lancet* 2001;357:763–7.
22 Carlsson S, Hammar N, Grill V, *et al*. Alcohol consumption and the incidence of type 2 diabetes: a 20-year follow-up of the Finnish twin cohort study. *Diabetes Care* 2003;26:2785–90.
23 Sargeant LA, Khaw KT, Bingham S, *et al*. Cigarette smoking and glycaemia: the EPIC-Norfolk Study. European Prospective Investigation into Cancer. *Int J Epidemiol* 2001;30:547–54
24 Wahid A, Manek N, Nichols M, *et al*. Quantifying the Association Between Physical Activity and Cardiovascular Disease and Diabetes: A Systematic Review and Meta-Analysis. *J Am Heart Assoc* 2016;5:e002495.
25 Narayan KM, Boyle JP, Thompson TJ, *et al*. Effect of BMI on lifetime risk for diabetes in the U.S. *Diabetes Care* 2007;30:1562–6.
26 Lebovitz HE, Banerji MA. Visceral adiposity is causally related to insulin resistance. *Diabetes Care* 2005;28:2322–25.
27 Ashwell M, Gunn P, Gibson S. Waist-to-height ratio is a better screening tool than waist circumference and BMI for adult cardiometabolic risk factors: systematic review and meta-analysis. *Obes Rev* 2012;13:275–86.
28 Jovicić S, Ignjatović S, Dajak M, *et al*. Association of lipid and inflammatory markers with C-reactive protein in cardiovascular risk assessment for primary prevention. *Clin Lab* 2009;55:411–9.
29 Seo MH, Bae JC, Park SE, *et al*. Association of lipid and lipoprotein profiles with future development of type 2 diabetes in nondiabetic Korean subjects: a 4-year retrospective, longitudinal study. *J Clin Endocrinol Metab* 2011;96:E2050–4.
30 Albert MA, Danielson E, Rifai N, *et al*. Effect of statin therapy on C-reactive protein levels: the pravastatin inflammation/CRP evaluation (PRINCE): a randomized trial and cohort study. *JAMA* 2001;286:64–70.
31 Kosmas CE, Silverio D, Sourlas A, *et al*. Impact of lipid-lowering therapy on glycemic control and the risk for new-onset diabetes mellitus. *Drugs Context* 2018;7:212562.
32 Lakoski SG, Cushman M, Palmas W, *et al*. The relationship between blood pressure and C-reactive protein in the Multi-Ethnic Study of Atherosclerosis (MESA). *J Am Coll Cardiol* 2005;46:1869–74.
33 Thorand B, Löwel H, Schneider A, *et al*. C-reactive protein as a predictor for incident diabetes mellitus among middle-aged men: results from the MONICA Augsburg cohort study, 1984-1998. *Arch Intern Med* 2003;163:93–9.
34 Thorand B, Baumert J, Kolb H, *et al*. Sex differences in the prediction of type 2 diabetes by inflammatory markers: results from the MONICA/KORA Augsburg case-cohort study, 1984-2002. *Diabetes Care* 2007;30:854–60.
35 Duncan BB, Schmidt MI, Pankow JS, *et al*. Low-grade systemic inflammation and the development of type 2 diabetes: the atherosclerosis risk in communities study. *Diabetes* 2003;52:1799–805.

For peer review only - http://bmjopen.bmj.com/site/about/guidelines.xhtml

36 Rubio-Martín E, Soriguer F, Gutiérrez-Repiso C, *et al*. C-reactive protein and incidence of type 2 diabetes in the Pizarra study. *Eur J Clin Invest* 2013;43:159–67.
37 Pradhan AD, Manson JE, Rifai N, *et al*. C-reactive protein, interleukin 6, and risk of developing type 2 diabetes mellitus. *JAMA* 2001;286:327–34.
38 Effoe VS, Correa A, Chen H, *et al*. High-Sensitivity C-Reactive Protein Is Associated With Incident Type 2 Diabetes Among African Americans: The Jackson Heart Study. *Diabetes Care* 2015;38:1694–700.
39 Lee CC, Adler AI, Sandhu MS, *et al*. Association of C-reactive protein with type 2 diabetes: prospective analysis and meta-analysis. *Diabetologia* 2009;52:1040–7.
40 Hu G, Jousilahti P, Tuomilehto J, *et al*. Association of serum C-reactive protein level with sex-specific type 2 diabetes risk: a prospective finnish study. *J Clin Endocrinol Metab* 2009;94:2099–105.
41 Kanmani S, Kwon M, Shin MK, *et al*. Association of C-Reactive Protein with Risk of Developing Type 2 Diabetes Mellitus, and Role of Obesity and Hypertension: A Large Population-Based Korean Cohort Study. *Sci Rep* 2019;9:4573.
42 Pan A, Wang Y, Yuan JM, *et al*. High-sensitive C-reactive protein and risk of incident type 2 diabetes: a case-control study nested within the Singapore Chinese Health Study. *BMC Endocr Disord* 2017;17:8.
43 Ahmadi-Abhari S, Kaptoge S, Luben RN, *et al*. Longitudinal association of C-reactive protein and Haemoglobin A1c over 13 years: the European Prospective Investigation into Cancer-Norfolk study. *Cardiovasc Diabetol* 2015;14:61.
44 Hovland A, Retterstøl K, Mollnes TE, *et al*. Anti-inflammatory effects of non-statin low-density lipoprotein cholesterol-lowering drugs: an unused potential? *Scand Cardiovasc J* 2020;54:274–9.
45 Oh RC, Lanier JB. Management of hypertriglyceridemia. *Am Fam Physician* 2007;75:1365–71.
46 Beshara A, Cohen E, Goldberg E, *et al*. Triglyceride levels and risk of type 2 diabetes mellitus: a longitudinal large study. *J Investig Med* 2016;64:383–7.
47 Overweight and obesity in Norway. Available: https://www.fhi.no/en/op/hin/health-disease/overweight-and-obesity-in-norway---/ [Accessed 19 Mar 2023].
48 Alberti KGMM, Eckel RH, Grundy SM, *et al*. Harmonizing the metabolic syndrome: a joint interim statement of the International Diabetes Federation Task Force on Epidemiology and Prevention; National Heart, Lung, and Blood Institute; American Heart Association; World Heart Federation; International Atherosclerosis Society; and International Association for the Study of Obesity. *Circulation* 2009;120:1640-5.
49 Michalsen VL Kvaløy K, Svartberg J, *et al*. Change in prevalence and severity of metabolic syndrome in the Sami and non-Sami population in rural Northern Norway using a repeated cross-sectional population-based study design: the SAMINOR Study. *BMJ Open* 2019;9:e027791.
50 Jacobsen BK, Eggen AE, Mathiesen EB, *et al*. Cohort profile: the Tromsø Study. *Int J Epidemiol* 2012;41:961–7.
51 Eggen AE, Mathiesen EB, Wilsgaard T, *et al*. The sixth survey of the Tromsø Study (Tromsø6) in 2007-08: collaborative research in the interface between clinical medicine and epidemiology: study objectives, design, data collection procedures, and attendance in a multipurpose population-based health survey. *Scand J Public Health* 2013;41:65–80.

For peer review only - http://bmjopen.bmj.com/site/about/guidelines.xhtml

52 The seventh survey of the Tromsø Study. Available: https://uit.no/research/tromsostudy/project?pid=708909 [Accessed 11 Sept 2021].
53 C-reactive protein concentrations as a marker of inflammation or infection for interpreting biomarkers of micronutrient status. World Health Organization. 2014. Available: https://apps.who.int/iris/handle/10665/133708 [Accessed 6 Aug 2023].
54 Stuart J, Whicher JT. Tests for detecting and monitoring the acute phase response. *Arch Dis Child* 1988;63:115–7.
55 Marchand A, Van Lente F, Galen RS. The assessment of laboratory tests in the diagnosis of acute appendicitis. *Am J Clin Pathol* 1983;80:369–74.
56 van Dieijen-Visser MP, Go PMNYH, Brombacher PJ. The value of laboratory tests in patients suspected of acute appendicitis. *Eur J Clin Chem Clin Biochem* 1991;29:749–52.
57 Peltola HO. C-reactive protein for rapid monitoring of infections of the central nervous system. *Lancet* 1982;319:980–3.
58 Clyne B, Olshaker JS. The C-reactive protein. *J Emerg Med* 1999;17:1019–25.
59 Håheim LL, Nafstad P, Olsen I, *et al*. C-reactive protein variations for different chronic somatic disorders. *Scand J Public Health* 2009;37:640-6.
60 Langholz PL, Wilsgaard T, Njølstad I, *et al*. Trends in known and undiagnosed diabetes, HbA1c levels, cardiometabolic risk factors and diabetes treatment target achievement in repeated cross-sectional surveys: the population-based Tromsø Study 1994-2016. *BMJ Open* 2021;11:e041846.
61 WHO collaborating centre for drug statistics methodology, Guidelines for ATC classification and DDD assignment, 2022. Available: https://www.whocc.no/filearchive/publications/2022_guidelines_web.pdf [Accessed 10 Jan 2022].
62 Browning LM, Hsieh SD, Ashwell M. A systematic review of waist-to-height ratio as a screening tool for the prediction of cardiovascular disease and diabetes: 0·5 could be a suitable global boundary value. *Nutr Res Rev* 2010;23:247–69.
63 Raised blood pressure (Indicator 11). Available: https://www.fhi.no/en/op/Indicators-for-NCD/blood-pressure/hoyt-blodtrykk-indikator-11/ [Accessed 19 Mar 2023]
64 Pace M LG, Glickman M, Grande E, *et al*. Revision of the European Standard Population, Report of Eurostat's task force, 2013 edition. Available: https://ec.europa.eu/eurostat/documents/3859598/5926869/KS-RA-13-028-EN.PDF/e713fa79-1add-44e8-b23d-5e8fa09b3f8f [Accessed 11 Sept 2021].
65 Ho KKN, Simic M, Småstuen MC, *et al*. The association between insomnia, c-reactive protein, and chronic low back pain: cross-sectional analysis of the HUNT study, Norway. *Scand J* Pain 2019;19:765–77.
66 Use of glycated haemoglobin (HbA1c) in the diagnosis of diabetes mellitus. Abbreviated report of a WHO consultation. Available: https://apps.who.int/iris/bitstream/handle/10665/70523/WHO_NMH_CHP_CPM_11.1_eng.pdf?sequence=1&isAllowed=y [Accessed 11 Sept 2021].
67 Lapice E, Maione S, Patti L, *et al*. Abdominal adiposity is associated with elevated C-reactive protein independent of BMI in healthy nonobese people. *Diabetes Care* 2009;32:1734–36.
68 Yoo EG. Waist-to-height ratio as a screening tool for obesity and cardiometabolic risk. *Korean J Pediatr* 2016;59:425–31.
69 Franceschi C, Campisi J. Chronic inflammation (Inflammaging) and its potential contribution to age-associated diseases. *J Gerontol A Biol Sci Med Sci* 2014;69(Suppl 1):S4–9.

For peer review only - http://bmjopen.bmj.com/site/about/guidelines.xhtml

70 Tabák AG, Herder C, Rathmann W, *et al*. Prediabetes: A high-risk state for developing diabetes. *Lancet* 2012; 379:2279–90.
71 Kato K, Otsuka T, Saiki Y, *et al*. Association between elevated C-reactive protein levels and prediabetes in adults, particularly impaired glucose tolerance. *Can J Diabetes* 2019;43:40–5.
72 Jaiswal A, Tabassum R, Podder A, *et al*. Elevated level of C-reactive protein is associated with risk of prediabetes in Indians. *Atherosclerosis* 2012;222:495–501.
73 Malka R, Nathan DM, Higgins JM. Mechanistic modeling of hemoglobin glycation and red blood cell kinetics enables personalized diabetes monitoring. *Sci Transl Med* 2016;8:359ra130.
74 Okura Y, Urban LH, Mahoney DW, *et al*. Agreement between self-report questionnaires and medical record data was substantial for diabetes, hypertension, myocardial infarction and stroke but not for heart failure. *J Clin Epidemiol* 2004;57:1096–103.
75 Schneider ALC, Pankow JS, Heiss G, *et al*. Validity and reliability of self-reported diabetes in the atherosclerosis risk in communities study. *Am J Epidemiol* 2012;176:738–43.
76 Yuan X, Liu T, Wu L, *et al*. Validity of self-reported diabetes among middle-aged and older Chinese adults: the China health and retirement longitudinal study. *BMJ Open* 2015;5:e006633.
77 van Woudenbergh GJ, Kuijsten A, Tigcheler B, *et al*. Meat consumption and its association with C-reactive protein and incident type 2 diabetes: the Rotterdam Study. *Diabetes Care* 2012;35:1499–505.
78 Løvsletten O, Jacobsen BK, Grimsgaard S, *et al*. Prevalence of general and abdominal obesity in 2015-2016 and 8-year longitudinal weight and waist circumference changes in adult and elderly: the Tromsø Study. *BMJ Open* 2020;10:e038465.
79 Pradhan AD, Manson JE, Rossouw JE, *et al*. Inflammatory biomarkers, hormone replacement therapy, and incident coronary heart disease: prospective analysis from the Women's Health Initiative observational study. *JAMA* 2002;288:980–7.
80 Albert MA, Glynn RJ, Burling J, *et al*. C-reactive protein levels among women of various ethnic groups living in the United States (from the Women's Health Study). *Am J Cardiol* 2004;93:1238–42.
81 Kelley-Hedgepeth A, Lloyd-Jones DM, Colvin A, *et al*. Ethnic differences in C-reactive protein concentrations. *Clin Chem* 2008;54:1027–37

For peer review only

FIGURE LEGENDS

Figure 1 – Conceptual framework.

Figure 2 – Study sample inclusion flow chart of the Tromsø Study 2007 – 2016.

Figure 3 – Age-standardized cumulative diabetes incidence across hs-CRP tertiles during follow-up in the Tromsø Study 2015 – 2016.

For peer review only

For peer review only - http://bmjopen.bmj.com/site/about/guidelines.xhtml

Figure 1

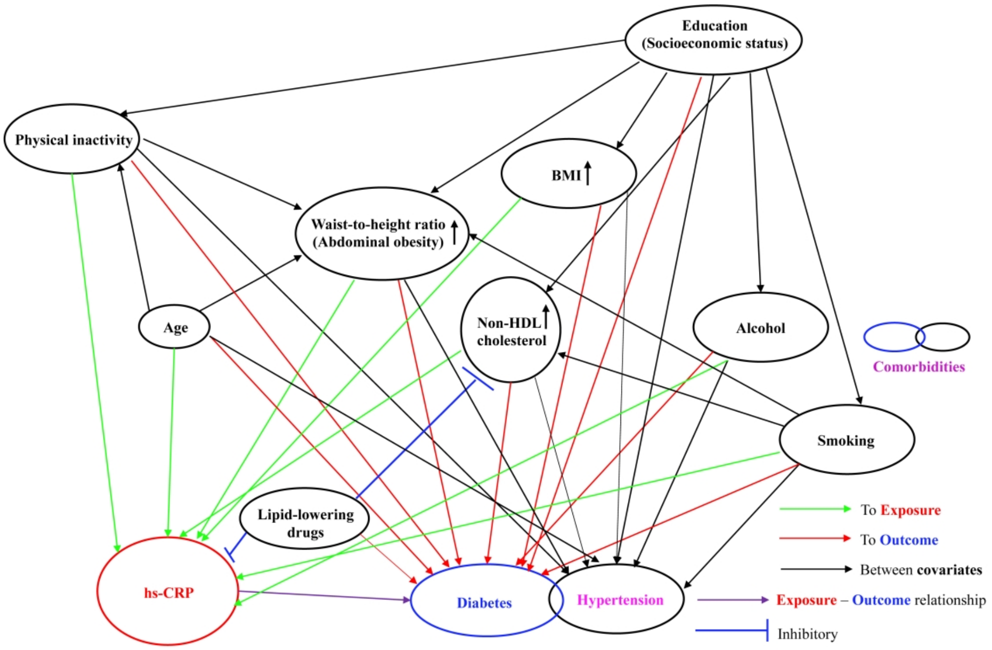

Figure 1. Conceptual framework

280x199mm (300 x 300 DPI)

Figure 2

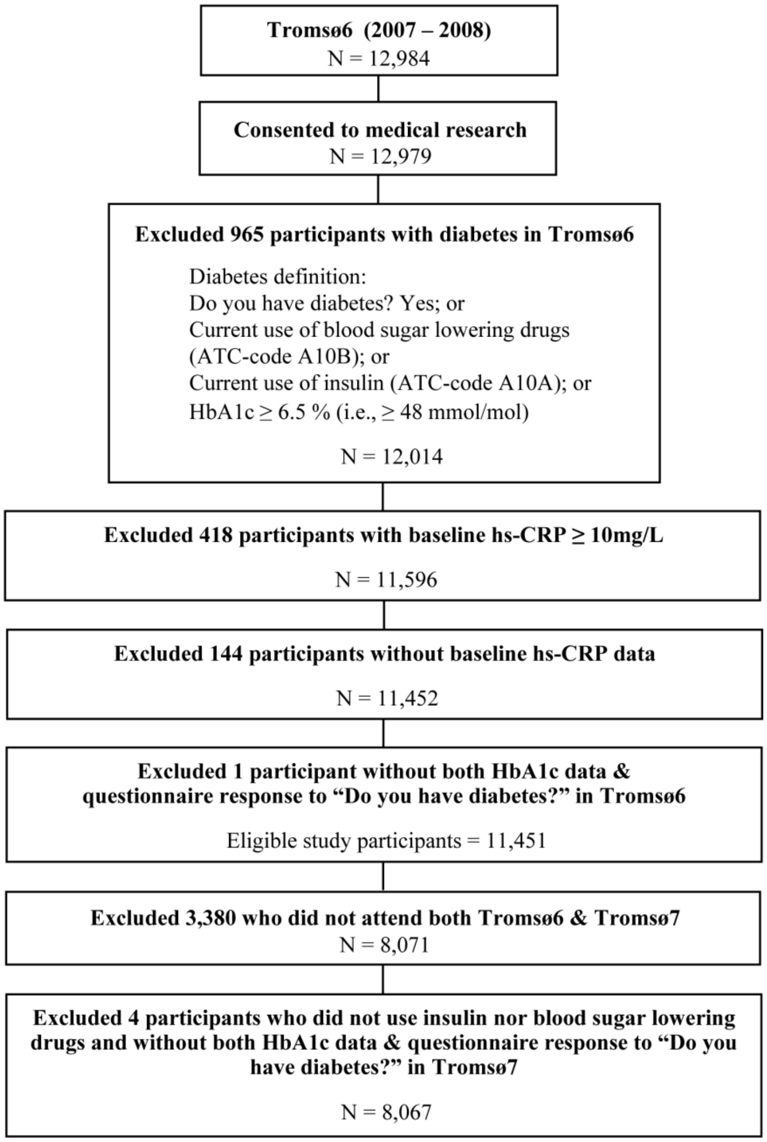

Figure 2. Study sample inclusion flow chart of the Tromsø Study 2007 – 2016

135x206mm (300 x 300 DPI)

Figure 3

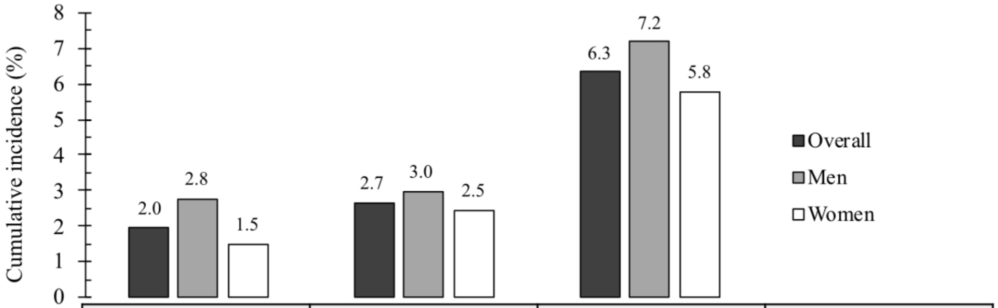

Tertile 1 (0.14 – 0.77)	Tertile 2 (0.78 – 1.69)	Tertile 3 (1.70 – 9.98)	hs-CRP (mg/L)
n=2,716	n=2,673	n=2,678	Overall (n=8,067)
n=1,139	n=1,336	n=1,229	Men (n=3,704)
n=1,577	n=1,337	n=1,449	Women (n=4,363)

Figure 3. Age-standardized cumulative diabetes incidence across hs-CRP tertiles during follow-up in the Tromsø Study 2015 – 2016

187x99mm (300 x 300 DPI)

STROBE Statement—Checklist of items that should be included in reports of ***cohort studies***

	Item No	Recommendation	Page No
**Title and abstract**	1	(*a*) Indicate the study's design with a commonly used term in the title or the abstract	2
		(*b*) Provide in the abstract an informative and balanced summary of what was done and what was found	2
**Introduction**			
Background/rationale	2	Explain the scientific background and rationale for the investigation being reported	4-5
Objectives	3	State specific objectives, including any prespecified hypotheses	5
**Methods**			
Study design	4	Present key elements of study design early in the paper	6
Setting	5	Describe the setting, locations, and relevant dates, including periods of recruitment, exposure, follow-up, and data collection	6-7
Participants	6	(*a*) Give the eligibility criteria, and the sources and methods of selection of participants. Describe methods of follow-up	Figure 1
		(*b*) For matched studies, give matching criteria and number of exposed and unexposed	N/A
Variables	7	Clearly define all outcomes, exposures, predictors, potential confounders, and effect modifiers. Give diagnostic criteria, if applicable	7-9
Data sources/ measurement	8*	For each variable of interest, give sources of data and details of methods of assessment (measurement). Describe comparability of assessment methods if there is more than one group	7-9
Bias	9	Describe any efforts to address potential sources of bias	7-9
Study size	10	Explain how the study size was arrived at	6, Figure 1
Quantitative variables	11	Explain how quantitative variables were handled in the analyses. If applicable, describe which groupings were chosen and why	9
Statistical methods	12	(*a*) Describe all statistical methods, including those used to control for confounding	9-10
		(*b*) Describe any methods used to examine subgroups and interactions	10
		(*c*) Explain how missing data were addressed	10
		(*d*) If applicable, explain how loss to follow-up was addressed	6
		(*e*) Describe any sensitivity analyses	10
**Results**			
Participants	13*	(a) Report numbers of individuals at each stage of study—eg numbers potentially eligible, examined for eligibility, confirmed eligible, included in the study, completing follow-up, and analysed	Page 11/Figure 1
		(b) Give reasons for non-participation at each stage	Figure 1
		(c) Consider use of a flow diagram	Figure 1
Descriptive data	14*	(a) Give characteristics of study participants (eg demographic, clinical, social) and information on exposures and potential confounders	11
		(b) Indicate number of participants with missing data for each variable of interest	Table 1
		(c) Summarise follow-up time (eg, average and total amount)	Fixed follow up time –

For peer review only

			explained page 6
Outcome data	15*	Report numbers of outcome events or summary measures over time	Table 2 & Figure 2

For peer review only

For peer review only - http://bmjopen.bmj.com/site/about/guidelines.xhtml

Main results	16	(*a*) Give unadjusted estimates and, if applicable, confounder-adjusted estimates and their precision (eg, 95% confidence interval). Make clear which confounders were adjusted for and why they were included	Table 2
		(*b*) Report category boundaries when continuous variables were categorized	Table 2
		(*c*) If relevant, consider translating estimates of relative risk into absolute risk for a meaningful time period	n/a
Other analyses	17	Report other analyses done—eg analyses of subgroups and interactions, and sensitivity analyses	12
**Discussion**			
Key results	18	Summarise key results with reference to study objectives	13
Limitations	19	Discuss limitations of the study, taking into account sources of potential bias or imprecision. Discuss both direction and magnitude of any potential bias	16,17
Interpretation	20	Give a cautious overall interpretation of results considering objectives, limitations, multiplicity of analyses, results from similar studies, and other relevant evidence	17
Generalisability	21	Discuss the generalisability (external validity) of the study results	16,17
**Other information**			
Funding	22	Give the source of funding and the role of the funders for the present study and, if applicable, for the original study on which the present article is based	23

*Give information separately for exposed and unexposed groups.

Note: An Explanation and Elaboration article discusses each checklist item and gives methodological background and published examples of transparent reporting. The STROBE checklist is best used in conjunction with this article (freely available on the Web sites of PLoS Medicine at http://www.plosmedicine.org/, Annals of Internal Medicine at http://www.annals.org/, and Epidemiology at http://www.epidem.com/). Information on the STROBE Initiative is available at http://www.strobe-statement.org.

For peer review only
